# MAGE: an efficient algorithm for identifying Maximal Age-indexed GEne sets

**DOI:** 10.1093/bib/bbag613

**Published:** 2026-11-02

**Authors:** Murat Çalış, Volkan Altuntas, Omer Kahveci, Pietro Hiram Guzzi, Pierangelo Veltri, Tamer Kahveci

**Affiliations:** Electronics Technology Programme, Electronics and Automation Department, Technical Sciences Vocational School, Bursa Uludağ University, Görükle Campus, Görükle Mahallesi, Üniversite 1. Cadde, No. 711G, Nilüfer, Bursa 16059, Türkiye; Department of Computer Engineering, Bursa Technical University, Bursa 16310, Türkiye; Department of Computer Science, University of Florida, Gainesville, FL 32611, United States; Department of Medical and Surgical Sciences, University of Catanzaro, Viale Europa, I-88100 Catanzaro, Italy; Department of Computer Engineering, Modelling, Electronic and Systems, University of Calabria, Via P Bucci, I-87036 Rende (CS), Italy; Department of Computer Science, University of Florida, Gainesville, FL 32611, United States

**Keywords:** Age Index, gene expression, aging, integrative genomics

## Abstract

Aging alters transcriptional regulation in a tissue- and time-specific manner, reshaping gene regulatory networks and shifting regulatory influence dynamics. Traditional *in silico* influence maximization methods often rely on static gene expression snapshots and fail to capture gene-to-gene influence variations over time. To address this, we introduce the *Age Index*, a network-based metric that quantifies time-varying gene influence. We define the *Maximal* and *Minimal Age Index Problems* (MAP$^{+}$ and MAP$^{-}$) as the tasks of identifying gene sets with the highest and lowest Age Index, and prove these problems are NP-hard. To solve them, we develop **MAGE** (Maximal Age-Index Algorithm), a stochastic method that iteratively constructs gene sets by prioritizing genes likely to optimize the Age Index. MAGE identifies statistically significant gene sets at the genome scale, with strong literature support, highlighting its potential to uncover age-sensitive regulatory mechanisms.

## Introduction

Aging is a progressive, time-dependent deterioration of physiological function and homeostatic capacity, driven by the accumulation of molecular and cellular damage and ultimately leading to increased susceptibility to disease and death. The study of aging mechanisms is therefore of fundamental importance, as it enables deeper insight into the regulatory processes that govern cellular dynamics and, crucially, the mechanisms underlying disease onset and progression [1].

Alterations in the cellular life cycle are tightly linked to aging, and different tissues—including blood, kidney, and skin—exhibit heterogeneous responses to the aging process. These tissue-specific trajectories arise from changes in gene expression that regulate cellular proliferation, differentiation, and maintenance. In some contexts, such alterations may drive tissues toward dysfunctional states associated with chronic and inflammatory diseases. As organisms age, they are continuously exposed to intrinsic and extrinsic stressors, leading to cumulative changes across behavioral, physiological, morphological, cellular, and molecular levels.

The cellular life cycle is fundamentally regulated by gene expression activity, which controls key biological processes such as protein synthesis and functional specialization. While growth and reproductive phases are well characterized, the relationship between aging and gene expression dynamics—particularly in relation to chronic disease onset—remains poorly defined. For instance, in neurodegenerative disorders such as Alzheimer’s disease [2], it is not possible to predict the temporal window during which gene expression changes begin to exert pathological effects. Consequently, elucidating the relationship between aging processes and transcriptional activity is essential for understanding how disruptions in the cellular life cycle contribute to disease development [2]. This necessitates the identification of robust indicators capable of capturing the complex, time-dependent interactions linking gene transcription, tissue-specific changes, and age-associated pathologies.

Aging is also strongly influenced by genomic instability, defined as the progressive accumulation of DNA damage, mutations, and chromosomal aberrations over time. These alterations compromise DNA integrity, disrupt chromatin organization, and impair transcriptional regulation, resulting in increased transcriptional variability and functional decline [3–6]. A central contributor to genomic instability is telomere shortening: telomeres are repetitive DNA–protein structures that protect chromosome ends from degradation, and their attrition is a well-established hallmark of aging [3, 4]. In parallel, age-associated epigenetic modifications—most notably changes in DNA methylation patterns—alter transcriptional regulation and gene expression, thereby contributing to functional deterioration and the emergence of age-related diseases [7–9]. Collectively, these molecular perturbations modify regulatory mechanisms governing the cellular life cycle, driving the multifactorial decline observed in aging organisms [10–13]. This underscores the importance of modeling the connections between aging, transcriptional deregulation, and systemic functional decline across tissues [10, 11, 14].

The tissue-specific effects of aging have been extensively investigated using large-scale transcriptomic and single-cell RNA sequencing technologies. The Genotype–Tissue Expression (GTEx) project provides a comprehensive resource of tissue-specific gene expression profiles from nearly 1000 individuals aged between 20 and 79 years [15], enabling systematic analysis of age-associated transcriptional changes across human tissues. Age-related transcriptional variation at single-cell resolution has also been explored in murine models [16], while meta-analyses have revealed conserved aging-related gene expression patterns and biological processes across tissues [17]. These studies demonstrate that gene expression profiles evolve with age, modulating a wide range of biological processes, cellular responses, and disease states [17]. Consequently, aging can be linked to both biological function and disease development [18], as well as to tissue-specific regulatory mechanisms [19, 20].

Numerous studies have shown that aging-related transcriptional changes differ markedly between tissues. For example, altered gene expression in the brain, muscle, skin, and vascular tissues has been associated with tissue-specific functional decline [19, 21]. In the nervous system, gene expression changes correlate with age-dependent alterations in neuronal activity [22], whereas in skeletal muscle, reduced expression of genes involved in cell renewal and protein synthesis contributes to sarcopenia [23]. Transcriptomic analyses stratified by age reveal that these changes are not uniform across tissues, highlighting the necessity of jointly modeling age, gene expression, and tissue specificity [24, 25]. Heart and lung tissues display pronounced co-aging patterns, indicative of synchronized transcriptional aging [24, 26], whereas skin exhibits particularly extensive age-associated gene expression changes [27].

To formally capture age-dependent transcriptional dynamics, it is essential to account for the mutual regulatory influences among genes. Such interactions are mediated by gene regulatory networks (GRNs), which encode relationships between transcription factors (TFs) and their target genes and play a central role in cellular function and longevity [1, 28–31]. As organisms age, the topology of these networks evolves, with shifts in node centrality reflecting changes in the regulatory influence of specific genes or TFs [32, 33]. Analyzing directional changes in gene influence within age-dependent GRNs provides a principled framework for understanding how regulatory mechanisms evolve over time and contribute to aging-related phenotypes [34, 35].

**Contributions.** In this article, we introduce a mathematical framework that explicitly links aging to transcriptional dynamics. Our model integrates GRNs with tissue-specific gene expression changes to quantify the influence of gene subsets on target tissues across defined age intervals. We propose an Age-Index formulation that captures the consistency of monotonic increases or decreases in gene-set influence with aging. This is formalized as an optimization problem—the Maximal and Minimal Age Index Problems (MAP$^{+}$ and MAP$^{-}$, respectively). To the best of our knowledge, this is the first study to define and address the MAP. We demonstrate that MAP is NP-hard and present a stochastic solution termed the Maximal Age-indexed GEne sets (MAGE) algorithm. We evaluate MAGE using transcriptional data from 17 382 samples spanning 54 tissues and 978 individuals from the GTEx database, together with human GRNs derived from the TRRUST database [36]. Our results show that MAGE scales to genome-wide analyses and identifies gene sets with high statistical significance for both maximal and minimal age indices. Moreover, independent evidence from the PubMed literature supports the association of the identified gene sets with aging, indicating that our framework effectively captures both ascending and declining regulatory trends over the lifespan. Finally, our findings demonstrate that the proposed model elucidates aging-related interactions among tissues mediated by transcriptional changes. Figure 1 presents an overview of the proposed framework.

**Figure 1:**
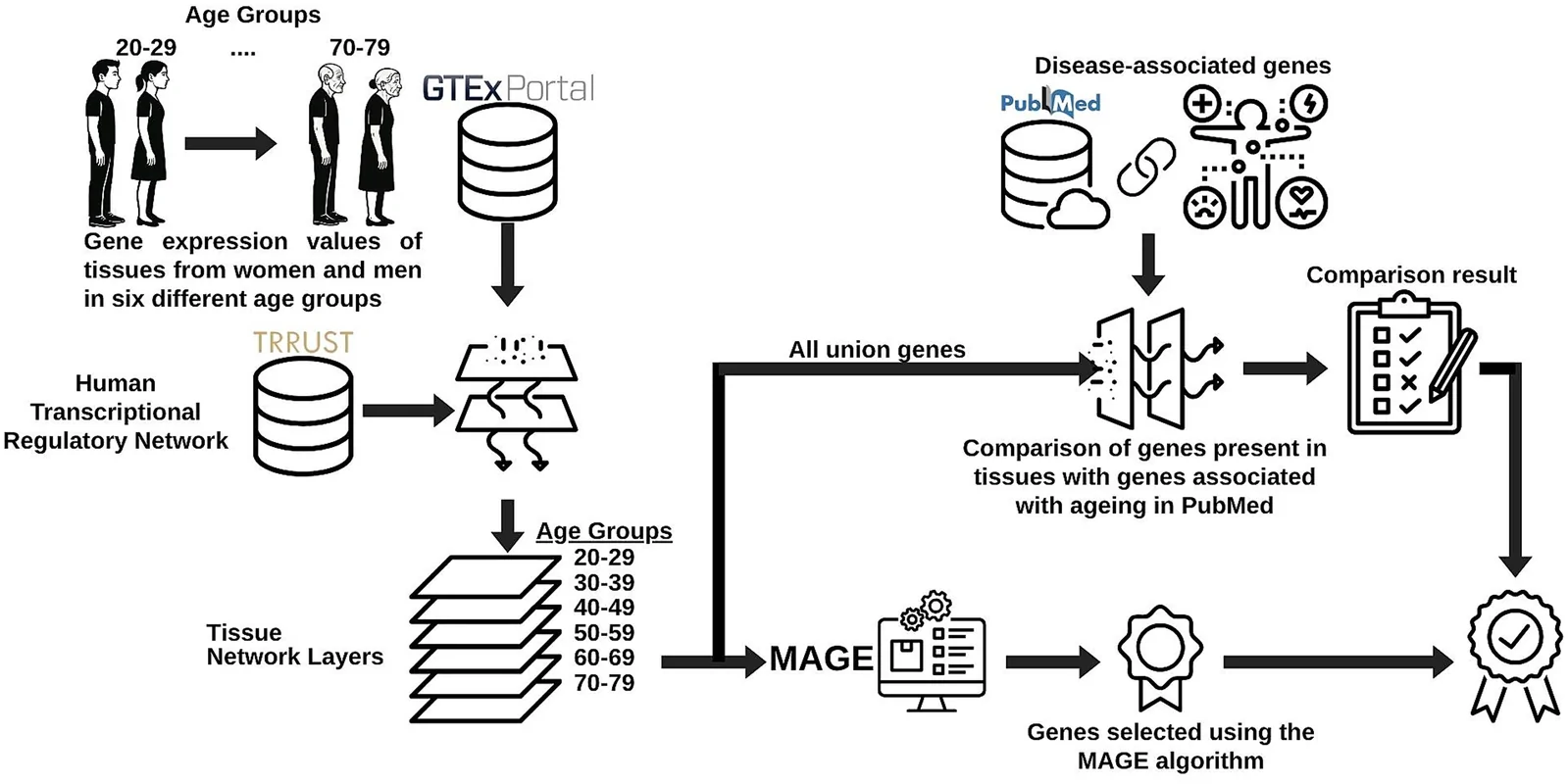
Overview of the MAGE algorithm. Gene expression data from the GTEx database are grouped by tissue and age into six intervals (20–29 to 70–79 years). For each tissue and interval, a tissue-specific GRN is constructed from TRRUST interactions and filtered using expression thresholds. Gene–gene influence is computed as an exponentially decaying function of shortest-path distance, $I(u,v \mid G)=e^{-\lambda d(u,v \mid G)}$, where *d(u,v ∣ G)* denotes the shortest-path distance between genes *u* and *v* in graph *G*, and *λ * is the influence decay parameter. MAGE then stochastically selects gene subsets that maximize or minimize MAGE scores, defined by consistent directional trends in influence across the resulting GRN sequence. This procedure identifies gene clusters whose regulatory impact steadily increases or decreases with age.

## Background

Intracellular processes undergo profound alterations with advancing age [5], motivating sustained investigation into the genetic and epigenetic mechanisms that underpin these changes. Early studies identified correlations between DNA methylation patterns and chronological age [22], enabling the estimation of biological age from methylome dynamics. Given the central role of DNA methylation in regulating gene expression, subsequent research has explored transcriptional changes associated with aging and age-related diseases [27]. For example, Frenk *et al*. [37] identified six gene-expression hallmarks of cellular aging, including the downregulation of mitochondrial proteins and the dysregulation of immune-related genes, thereby delineating key molecular features of aging. Balliu *et al*. [38] reported 1291 genes exhibiting significant age-dependent expression changes alongside attenuated regulatory contro.

More recently, relationships between gene expression and aging have been investigated to shed light on gene activity, genomic instability, and the cellular life cycle across human tissues. Tissue-specific transcriptional shifts have been studied extensively, largely using the GTEx project as a reference. GTEx provides a comprehensive catalog of age-associated gene expression patterns across multiple tissues, highlighting both shared and tissue-specific aging signatures, as well as their overlap with genes implicated in age-related diseases [39]. Using GTEx data, Yamamoto *et al*. [40] uncovered systemic transcriptional alterations, notably a decline in mitochondrial and translation-related gene expression, accompanied by increased expression variability in selected tissues. Expression dynamics in the aging brain, together with comparative analyses across multiple murine tissues [41], have further refined our understanding of tissue-dependent aging processes. Age-related transcriptional changes have also been studied in the context of chronic diseases, with particular emphasis on associations between aging and pathological gene activity [6]. Meta-analyses comparing young and old individuals across species have consistently identified genes with conserved differential expression trajectories, with subsets exhibiting either increased or decreased expression with age [10, 27, 42]. Collectively, these findings suggest that gene expression–age relationships can influence lifespan [37, 43], underscoring their potential causal role in the aging process.

These studies support the hypothesis that aging is, at least in part, genetically regulated. However, a fundamental question remains unresolved: do transcriptional changes act as causal drivers of aging, or are they merely downstream consequences of the aging process [44]? Recent work has sought to address this issue by providing reproducible methodologies and open data resources, thereby clarifying the genetic and regulatory contributions to aging [45]. Within this context, defining age-related indices derived from gene expression dynamics has emerged as a promising strategy to quantify and interpret temporal regulatory shifts.

GRNs have also been used as a framework for studying interactions among genes, cellular states, tissue identity, and aging. GRNs enable the identification of disrupted regulatory mechanisms that may underlie age-associated phenotypes. For instance, aging-related GRN dynamics were used in [46] to characterize transitions between cellular states such as homeostasis, arrest, and senescence. Similarly, GRNs have been used to identify key modulators of chronological lifespan in yeast [29]. Age-related regulatory changes in lung cells during coronavirus infection were investigated in [47] using GRNs.

The predictive accuracy of aging-related gene signatures has also been improved by integrating gene expression profiles with protein–protein interaction data [48]. Boolean network modeling has been applied to dissect regulatory changes underlying early gene expression variation (e.g. in hematopoiesis), including age-associated alterations in hematopoietic stem cells [49]. More broadly, aging trajectories—reproducing observed patterns in mortality and health status across age groups [50, 51]—have been modeled using computational simulations based on health-attribute networks.

Taken together, existing studies highlight the need to relate gene expression, genomic instability, and aging within a formal modeling framework. Aging is a dynamic, multiscale process shaped by gene regulation, environmental exposure, and systemic physiological feedback, making generalization intrinsically complex. Most existing approaches for identifying aging-associated genes rely on static network representations and largely neglect temporal variation in regulatory architecture. As a result, they tend to prioritize genes with high global influence, irrespective of how regulatory roles evolve over time. Aging, however, is inherently directional and progressive, suggesting that influence metrics should explicitly capture monotonic trends in gene activity. To address this gap, we propose an age-index methodology to relate gene expression to aging. The MAGE algorithm operationalizes this idea by identifying gene subsets whose regulatory influence changes consistently with age, thereby offering a more faithful representation of age-dependent network dynamics. In summary, although previous research has elucidated many aspects of age-associated gene expression, existing models frequently overlook temporal dynamics and coordinated regulatory shifts across the lifespan. Most approaches focus on isolated genes or static metrics—such as expression levels or network centrality—without capturing time-dependent trends or system-wide patterns of influence. Consequently, they fail to reflect the intrinsically directional nature of aging as a biological process, motivating the development of mathematical models that explicitly incorporate temporal effects in gene regulation. Existing computational approaches to aging analysis predominantly rely on differential expression analysis, epigenetic aging clocks, or static network-based metrics. Differential expression methods identify genes whose expression correlates with age, but they do not capture coordinated regulatory influence or network-level dynamics. Aging clocks prioritize predictive accuracy, often at the expense of mechanistic interpretability. Network-based studies typically focus on static topological properties—such as centrality or connectivity—without modeling how regulatory influence evolves across age intervals. In contrast, MAGE formulates aging as a time-dependent network influence optimization problem. By integrating GRN structure with age-stratified expression data, MAGE identifies gene sets whose collective regulatory influence changes consistently with age. This enables the discovery of coordinated, system-level aging mechanisms that are not accessible through static or gene-centric approaches.

## Methods

We define the concept of *Age Index*, a network-based metric used to quantify time-varying gene influence and its relation with aging. We develop a novel stochastic algorithm to find the gene set that is collectively associated with aging. We focus on identifying gene sets with the highest and lowest Age Index, by using **MAGE** (Maximal Age-Index Algorithm), a method that iteratively constructs gene sets by prioritizing genes likely to optimize the Age Index. In the following text, preliminaries and problem definition are presented. Examples of data preparation used to create gene network sequence for each tissue are also reported.

### Preliminaries and problem definition

We use network models to represent the relations among genes. Networks are used to map gene-to-gene interaction and to model the time related (aging) evolution. A GRN is modeled as a directed graph, in which vertices and edges correspond to genes and their regulatory interactions, respectively. The sets of vertices and edges are denoted by * V * and * E *, and the resulting graph is represented by the tuple * G = (V, E) *. Each directed edge encodes either an activation or a repression relationship between a pair of genes.

Genes modulate each other’s transcriptional activity through these regulatory interactions. When two genes are connected within the underlying GRN by a chain of regulatory interactions, they may exert indirect transcriptional influence on another one. This influence is assumed to decrease as the length of the interaction chain increases. To capture this behavior, the influence is modeled as an exponentially decaying function governed by a decay parameter * λ *.

Formally, given a graph * G = (V, E) *, the distance between two nodes * u, v ∈ V * is defined as the minimum number of edges in the shortest path connecting them, and is denoted by * d(u, v ∣ G) *. Using this definition, the notion of regulatory influence between genes can be formally introduced.

Definition 1 (influence of gene on gene). Assume that we are given a graph *G = (V, E)* corresponding to a GRN. Consider two nodes *u, v ∈ V*. We denote the influence of *u* on *v* in *V* as *I(u, v|G)* and define it as

\begin{align*} &I(u, v|G) = e^{-\lambda d(u,v | G)}\end{align*}

The larger the value of the decay parameter *λ * is, the faster the influence drops between genes that are far away on the GRN.

Definition 2 (influence of gene set on gene). Assume that we are given a graph *G = (V, E)* corresponding to a GRN. Consider a non-empty subset of genes $V^{\prime} \subseteq V$ and a gene *v ∈ V*. We denote the influence of the set of genes $V^{\prime}$ on the target node *v* as $I(V^{\prime},v | G)$ and define it as

\begin{align*} &I(V^{\prime}, v | G) = \max_{u \in S} \{e^{-\lambda d(u,v | G)}\}\end{align*}

In other words, the influence of a set of genes on a target gene is equal to the influence of the closest gene in that set to the target gene. Next, we define the influence of a gene set on the entire network composed of all the genes.

Definition 3 (influence of gene set on cell). Assume that we are given a graph *G = (V, E)* corresponding to a GRN. Consider a non-empty subset of genes $V^{\prime} \subseteq V$ and a gene *v ∈ V*. We denote the influence of the set of genes $V^{\prime}$ on *G* as $I(V^{\prime},G)$ and define it as

\begin{align*} &I(V^{\prime}, G) = \sum_{v \in V} I(V^{\prime}, v | G)\end{align*}

GRNs are not static network topologies. With aging, the wiring of the interactions in GRNs evolves as the activity levels of genes change, leading to an ordered sequence (or simply ”sequence”) of network topologies. As a result, the influence of a set of genes on the cell also changes with aging. Let us denote the topologies of the GRNs of a particular tissue observed at two different ages $a_{1}$ and $a_{2}$ ($a_{1} < a_{2}$) with $G_{a_{1}}=(V, E_{a_{1}})$ and $G_{a_{2}}=(V, E_{a_{2}})$. We define a trajectory indicator function, *ψ ()* over a subset of genes $V^{\prime} \subseteq V$, and the two GRNs at two different ages $a_{1}$ and $a_{2}$ to indicate whether the influence of the genes in $V^{\prime}$ on the cell has increased by at least a minimum observable amount *ε *, stayed steady, or decreased by at least *ε * as the age increased from $a_{1}$ to $a_{2}$ as


\begin{align*}& \psi(V^{\prime}, G_{a_{1}}, G_{a_{2}}) = \begin{cases} \ \ 1 & \textrm{if}\ I(V^{\prime},G_{a_{1}})> I(V^{\prime},G_{a_{2}}) + \epsilon\\ \ -1 & \textrm{if}\ I(V^{\prime},G_{a_{1}}) < I(V^{\prime},G_{a_{2}}) - \epsilon\\ \ \ 0 & \textrm{else} \end{cases}. \end{align*}

We use *ε = 0.005* (i.e. 0.5% of the maximum observed transcription value for each gene) as the default minimum observable change in our experiments. Given two positive integers *x,y* with *x>y*, let us denote **x* choose *y*”* with $C^{x}_{y}$. Using the indicator function, we define the Age Index of a set of genes next.

Definition 4 (Age Index). Assume that we are given a sequence of *t* GRNs observed at different ages $a_{1}$, $a_{2}$, …, $a_{t}$ with $\leq i < t, a_{i} < a_{i+1}$. Let us denote the topology of the GRN observed at age $a_{i}$ in this sequence with $G_{a_{i}} = (V, E_{a_{i}})$. Consider a subset of genes $V^{\prime} \subseteq V$. We denote the Age Index of $V^{\prime}$ on the sequence of GRNs $\mathcal{G} =$  $[G_{a_{1}}$, $G_{a_{2}}$, …, $G_{a_{t}}]$ with $AI(V^{\prime}, \mathcal{G})$, and compute it as

\begin{align*} & AI(V^{\prime},\mathcal{G}) =\frac{1}{C^t_2} \sum_{i<j} \psi(V^{\prime}, G_{a_i}, G_{a_j})\end{align*}

The Age Index of a set of genes takes a value in the *[-1, 1]* interval. The sign of the Age Index shows the direction of the trajectory of the influence of those genes with aging: a positive/negative sign indicates overall increasing/decreasing influence with aging. The magnitude of the Age Index shows the consistency of the trajectory. For instance, an Age Index with a large positive (negative) value indicates mostly increasing (decreasing) influence of the given gene set on the cell with aging. Notice that, regardless of the sign, large magnitude values of the Age Index indicate sets of genes whose collective influence on the cell (almost) monotonically changes with aging, thus they are associated with aging with a high likelihood. On the other hand, values close to zero imply that the influence of the given gene set fluctuates with aging and thus they are not associated with aging.

Example 1. Figure 2 shows real scenarios that we observe in the GTEx and TRRUST databases. The figure shows four alternative trajectories of influences of a subset of genes on four tissues. The top left panel shows a set of three genes, {HR, SOX2, FOXA1}. The influence of this set of genes on the stomach increases consistently with aging, although the increase is somewhat slow from the [40,49] to [50,59] age interval. Thus, this gene set has high positive Age Index. The top right shows the gene set {HR, APC, HMGA1}, whose influence on muscle skeletal tissue is consistently declining with aging. As a result, this gene set has a large magnitude negative Age Index. The bottom left set of genes, i.e. {FOXA3, NKX2-5, GATA1}, has fluctuating influence on spleen with aging. Therefore, the Age Index of this gene set is close to zero. The influence of the bottom right gene set {FOXF1, BATF, BHLHE41} on brain cerebellar hemisphere remains steady with aging. The Age Index of this gene set is exactly zero. Notice that the genes in the top two figures (with large magnitude positive or negative Age Index values) have a large number of publication evidence items associating them with aging in the literature. However, the genes in the two bottom figures have little or no publication evidence. This exemplifies the biological significance of the Age Index measure defined here.

**Figure 2:**
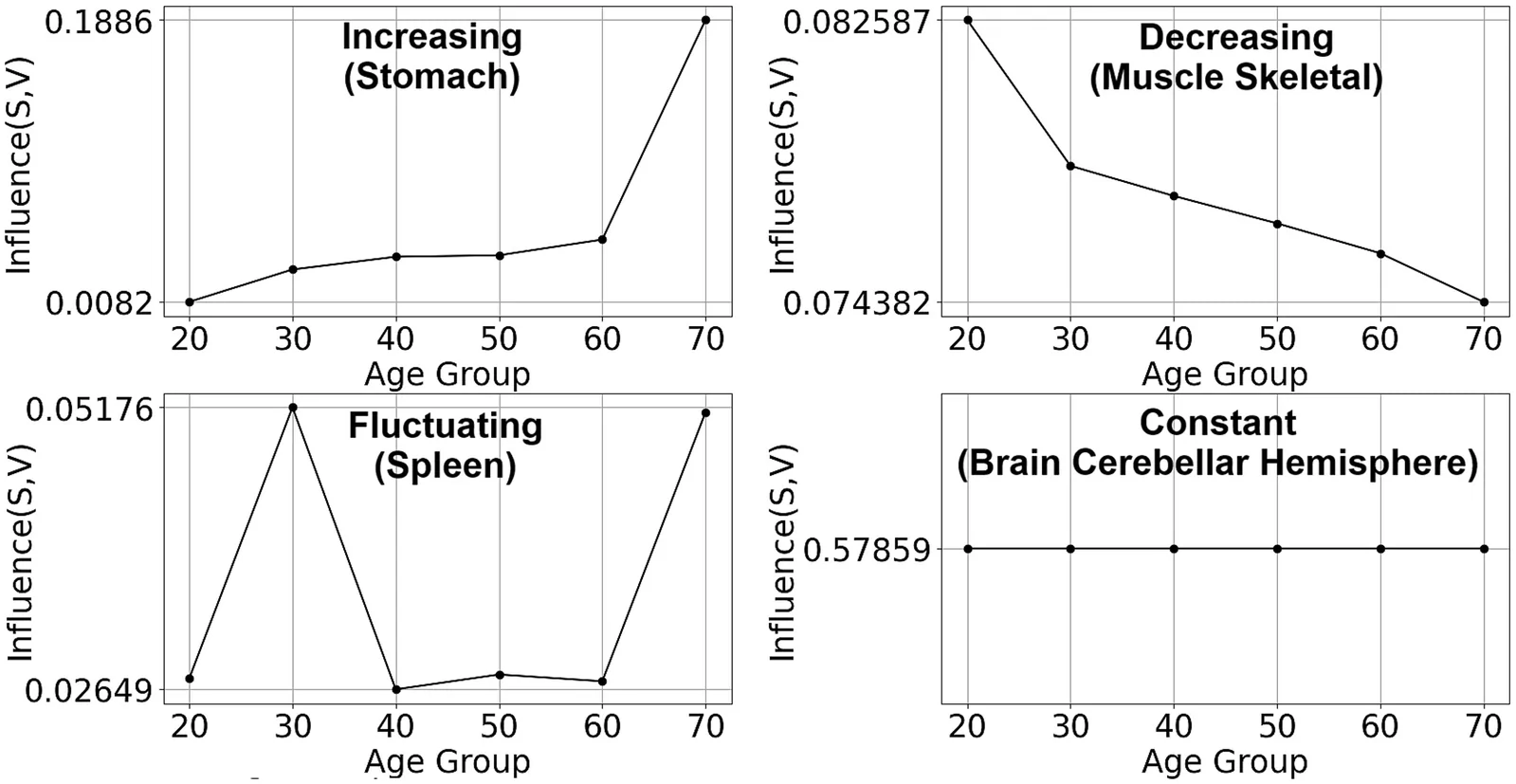
Four influence pattern examples on four tissues. The *x*-axis is the starting year of 10-year age intervals. For instance, 20 means the age interval [20–29].The *y*-axis is the influence of the given gene set on the given tissue for that age interval. In our notation below, in the set notation, *A:n*, *A* indicates gene name and *n* indicates the number of publications in PubMed, which include having gene *A* and the keyword ”aging” together. The figure illustrates four temporal influence patterns of gene sets from four tissues: (i) Top left = Increasing influence on stomach – {HR: 508, SOX2: 32, FOXA1: 4}, (ii) Top right = Decreasing influence on skeletal muscle – {HR: 508, APC: 86, HMGA1: 2}, (iii) Bottom left = Fluctuating influence on the spleen – {FOXA3: 2, NKX2-5: 2, GATA1: 4}, and (iv) Bottom right = Steady (constant) influence on brain cerebellar hemisphere – {FOXF1:0, BATF:0, BHLHE41:0}.

**Problem definition (Maximal Age Index Problem (MAP$^{+}$):**  *A*ssume that we are given a sequence of *t* GRNs $\mathcal{G} =$  $[G_{a_{1}}$, $G_{a_{2}}$, …, $G_{a_{t}}]$, observed at ages $a_{1}$, $a_{2}$, …, $a_{t}$ with $1 \leq i < t, a_{i} < a_{i+1}$, and $G_{a_{i}} = (V, E_{a_{i}})$. The MAP$^{+}$ problem seeks to find the subset of genes $V^{*} \subseteq V$, such that


\begin{align*} \forall V^{\prime} \subseteq V, AI(V^{\prime},\mathcal{G}) \leq AI(V^{*},\mathcal{G}) \end{align*}

Theorem 1.1. MAP$^{+}$ is NP-Hard.

We provide the proof of Theorem 1.1 in Supplementary Materials Section SM. 1. The dual of the MAP$^{+}$ problem is the MAP$^{-}$, which seeks the subset of *k* genes with the minimum possible Age Index. Both problems belong to the same complexity class. Furthermore, we prove that the MAP$^{+}$ problem has five properties: lack of monotonicity, submodularity, smoothness/continuity, bounded marginal gains, and cardinality constraints. Collectively, these five properties make it impossible to derive approximation bounds to the MAP$^{+}$ problem in polynomial time.

### Data

We consider the GTEx database [52] and the curated transcriptional regulatory interactions provided by the TRRUST database [53]. GRN sequence data are derived from both complementary resources, GTEx and the TRRUST database. The GTEx project provides tissue- and cell-specific gene expression profiles across 54 human tissues obtained from 948 deceased individuals, yielding a total of 17 382 samples. Donors span an age range from 21 to 79 years. The TRRUST database comprises 800 human TFs, 8444 TF–target regulatory interactions, and 2862 unique genes, curated from 11 237 PubMed articles describing experimentally validated transcriptional regulatory relationships.

Interactions from TRRUST are used to define a baseline human GRN * G = (V, E) *. The GTEx transcriptional dataset is subsequently employed to derive age- and tissue-specific GRNs. For each tissue, samples are stratified into nonoverlapping 10-year age intervals, namely $[20\mathrm{--}29]$, $[30\mathrm{--}39]$, *… *, and $[70\mathrm{--}79]$, resulting in six age groups. These intervals are indexed by their lower bounds and denoted by $ a_{20}, a_{30}, \dots , a_{70} $, respectively. To reduce the impact of extreme expression values, the top 1% of transcription measurements are removed for each gene within each tissue–age group combination. A representative transcriptional profile is then constructed by computing the mean expression value across all remaining samples for each tissue and age interval. Tissues lacking samples in any age group are excluded. Following this filtering procedure, 48 tissues remain with complete transcriptional coverage across all six age intervals.

For a given tissue $ T_{i} $ and age interval $ a_{j} $, the corresponding age- and tissue-specific GRN is defined as


\begin{align*} & G^{T_{i}}_{a_{j}} = (V, E^{T_{i}}_{a_{j}}), \end{align*}

where the mean transcription level of gene * v ∈ V * is denoted by $ \mu (v, T_{i}, a_{j}) $. Given a predefined interaction threshold * γ *, the edge set $ E^{T_{i}}_{a_{j}} \subseteq E $ is constructed such that, for all * u, v ∈ V *, an edge $ (u, v) \in E^{T_{i}}_{a_{j}} $ if and only if (i) * (u, v) ∈ E *, indicating a documented regulatory interaction; and (ii) $ \mu (v, T_{i}, a_{j}) \geq \gamma $, indicating sufficient transcriptional activity of the target gene.

This procedure yields, for each tissue $ T_{i} $, a temporally ordered sequence of six age-specific GRNs,


\begin{align*} & \left[ G^{T_{i}}_{a_{20}}, G^{T_{i}}_{a_{30}}, \dots, G^{T_{i}}_{a_{70}} \right], \end{align*}

which collectively capture age-dependent regulatory dynamics at the tissue level.

### MAGE algorithm

The MAGE algorithm solves both the MAP$^{+}$ and the MAP$^{-}$ by negating the Age Index value. MAGE is based on a stochastic heuristic procedure that incrementally constructs a solution set of genes, starting from the empty set and iteratively adding genes until the desired cardinality is reached. At each iteration, a gene is selected at random according to its likelihood of improving the objective function. Pseudocode for the MAGE algorithm is reported in Algorithm 1.

MAGE takes four input parameters: (i) a sequence of age-specific GRNs, $\mathcal{G} = [G_{a_{1}}, G_{a_{2}}, \dots , G_{a_{t}}]$, (ii) an influence decay parameter * λ *, which controls the rate at which the regulatory influence of a source gene on a target gene decreases with increasing network distance, (iii) the number of genes * k * to be selected, and (iv) a randomization parameter $ \beta \in [0, \infty ] $, which governs the level of stochasticity in the gene selection process. Specifically, * β = 0 * corresponds to deterministic gene selection, whereas $ \beta = \infty $ results in completely random selection.



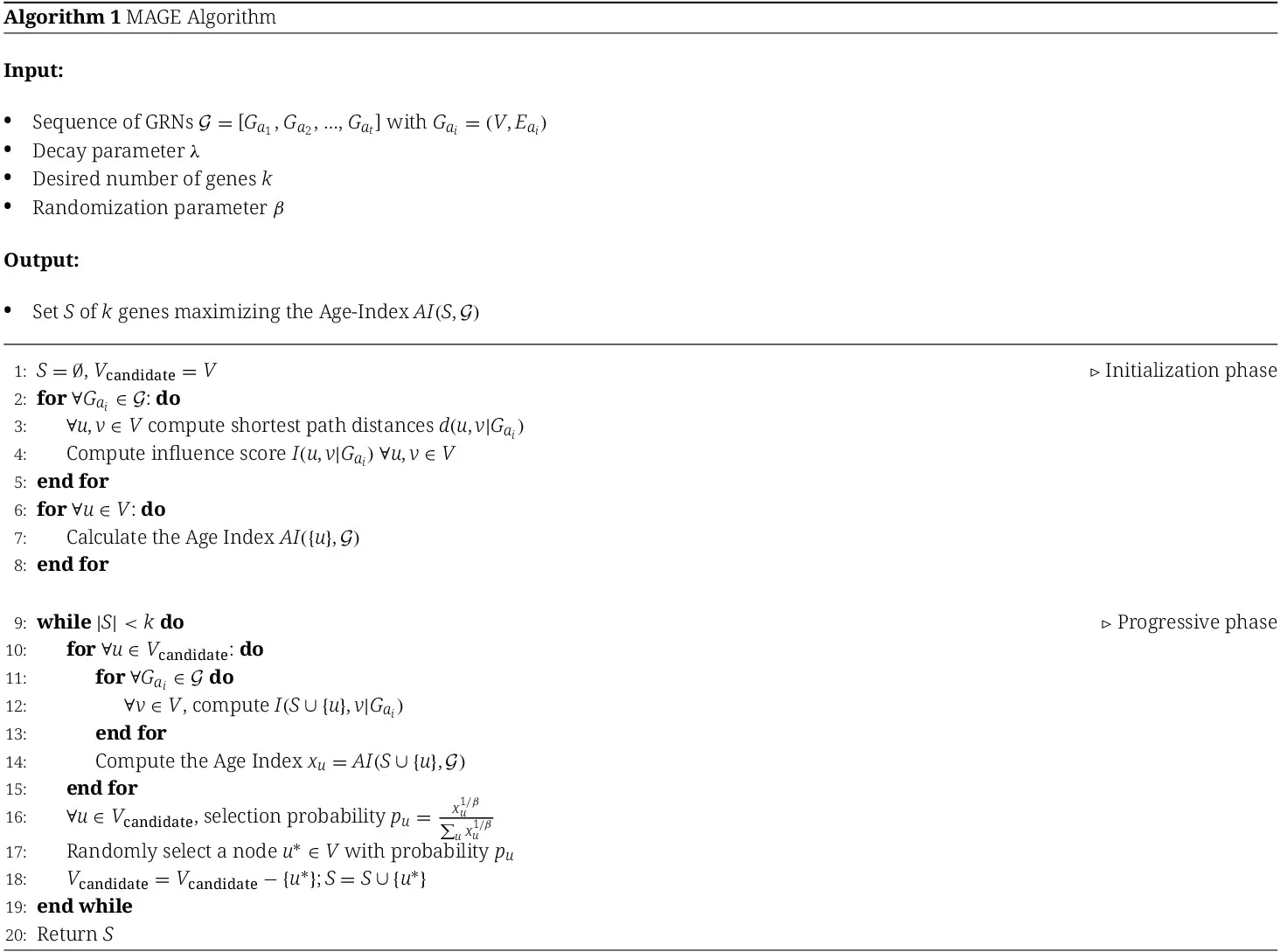



**Phase 1: Initializations.** (lines 1 to 8) MAGE starts by initializing the solution set *S* to the empty set, and the candidate gene set to the set of all genes (line 1). It then computes and stores the shortest path distance for all gene pairs in each of the networks in the sequence. We use Johnson’s all-to-all shortest path algorithm [54] due to its low expected time complexity for this purpose (line 3). Using these values, MAGE computes the Age Index for all the genes (lines 4 to 8).

**Phase 2: Progressive solution construction.** (lines 9–19) In this phase, the algorithm repeatedly grows the solution set by adding one gene at a time at each iteration. For each gene in the candidate gene set, MAGE computes the influence of the solution set, after inserting only that particular gene into the current solution set for each GRN in the sequence (lines 11–13). Notice that computing the influence of a set of genes *S* on another gene *v ∈ V* requires knowing the shortest distance between *S* and *v*. When we grow *S* by inserting a gene *u* into *S*, the shortest distance between $S \cup \{u\}$ and *v* is simply the minimum of the two values: (i) distance between *S* and *v*, (ii) distance between *u* and *v*. Since both of these values are stored at the end of the initialization phase, this requires a simple selection between the two numbers in *O(1)* time rather than the costly recomputation of the distances between $S \cup \{u\}$ and *v* from scratch.

Using the influence values computed, MAGE computes the Age Index value for each possible $S \cup \{u\}$, denoted by variable $x_{u}$ (line 14). Next, for each candidate gene *u*, it translates the Age Index $x_{u}$ to a selection probability $p_{u}$, proportional to $x_{u}^{1/\beta }$ (line 16). Notice that the sum of all probabilities $p_{u}$ over all candidate genes is equal to 1. Finally, MAGE adds one gene randomly among the candidate genes with selection probability $p_{u}$, adds it to the solution set and removes it from the candidate gene set (lines 17 and 18). MAGE repeats the progressive construction until the solution set contains the desired number of genes.

**Time complexity of MAGE.** The all-to-all shortest path algorithm for each $G_{a_{i}} = (V, E_{a_{i}})$ (line 3 of the MAGE Algorithm) takes $O(|V|^{2}\log (|V|+|V||E_{a_{i}}|))$ time. Computing the influence score over all node pairs takes $O(|V|^{2})$ time since each node pair takes *O(1)* time. As a result, initializing each network $G_{a_{i}}$ in the sequence costs $O(|V|^{2}\log (|V|+|V||E_{a_{i}}|)$ time, leading to a total initialization time of


\begin{align*} &\sum_{i=1} ^t O(|V|^2\log(|V|+|V||E_{a_i}|))\end{align*}

Through algebraic manipulation, this is equal to


\begin{align*} &O\left(t|V|^2\log\left(|V|+|V|\sum_{i=1}^t|E_{a_i}|\right)\right)\end{align*}

Since GRNs are sparse, approximating $O(|E_{a_{i}}|) = O(|V|)$ reduces the complexity to


\begin{align*} &O(t|V|^2\log(|V|))\end{align*}

In the progressive phase, at each iteration, for every candidate gene and target gene pair we need to update the influence (lines 11–13). These updates dominate the cost of this phase. Each update costs constant time. The number of updates per GRN is $O(|V|^{2})$. Since there are *t* GRNs, the total cost is $O(t|V|^{2})$. Since we have a total of *k* iterations, the total cost of this phase is $O(tk|V|^{2})$. Thus the total time complexity of MAGE is


\begin{align*} &O(t|V|^2 (\log(|V| + k))\end{align*}

**Stochasticity of the MAGE algorithm.** The MAGE algorithm can be applied with different values of parameter *β *. As the value of *β * approaches *∞ *, *1/β * approaches to 0. As a result, for all genes *u*, $x_{u}^{1/\beta }$ approaches to 1. Thus, all the candidate genes have the same selection probability of $p_{u} = 1/|V_{\mathrm{candidate}}|$. In this extreme case, MAGE acts like an entirely random selection algorithm disregarding the node influence values. As we reduce the value of *β *, MAGE starts favoring the genes that increase the Age Index most at each step. At the other extreme, as the value of *β * approaches to zero, *1/β * approaches *∞ *. As a result, the selection probability $p_{u}$ of the node *u* with the largest increase in the Age Index of $S \cup \{u\}$ approaches to one, and those of all other genes approach to zero. Thus, MAGE acts as a deterministic algorithm that greedily adds the most profitable gene to the solution set at each iteration. If there are multiple genes with the same largest increase in Age Index, MAGE randomly picks one of them as they all have the same probabilities. In general, if *β * = 0 we have a deterministic instance of MAGE, while for *β> 0* MAGE runs in a stochastic version.

### Gene regulatory network construction and sensitivity considerations

MAGE operates on inferred GRNs, which are constructed using correlation-based and regression-driven inference methods commonly adopted in large-scale transcriptomic studies. These approaches assume that consistent co-variation and conditional dependence between gene expression profiles reflect potential regulatory interactions, while acknowledging that inferred GRNs represent statistical rather than causal relationships.

The Age Index formulation is primarily driven by relative changes in regulatory influence across age intervals rather than absolute edge weights. Here, we quantify the “influence” to represent topological and statistical association pathways within an inferred network context. As a result, the method is expected to be moderately robust to variations in network inference strategies, provided that the overall topology and dominant regulatory relationships are preserved. Alternative GRN inference methods, such as mutual information-based or Bayesian approaches, may yield differences in edge composition; however, MAGE focuses on identifying gene sets whose aggregated influence exhibits consistent age-dependent trends, reducing sensitivity to individual edge-level fluctuations.

Regarding edge-weight uncertainty, stochastic variation or noise in individual regulatory strengths is partially mitigated by the aggregation of influence across multiple genes and network paths. Similarly, network sparsity or density primarily affects the scale of influence values rather than their relative ordering across age groups, which underlies the optimization objective of MAGE. Nonetheless, we acknowledge that extreme variations in GRN construction may impact quantitative results, and systematic evaluation across alternative network inference frameworks constitutes an important direction for future work.

## Experimental results

We tested MAGE under different parameter configurations and also aiming to demonstrate the importance of the Age Index in uncovering biologically meaningful results.

We used a GRN obtained from the TRRUST database (version 2) [36], considering 800 human TFs, 8444 TF–target interactions, and 2862 unique genes. Moreover, we used the GTEx dataset to map tissue and age to a specific GRN topology. We performed experiments using different values of *λ *, *ε *, and *β *. For example, with *λ = 0.3*, MAGE enabled the inclusion of an interaction in the GRN of a given tissue if the mean transcription value of the underlying TF gene was at least 30.

For GRNs with a sufficient number of tissues, we conducted a preprocessing and filtering phase using different *λ * values. Tissues mapped to GRNs with few samples were filtered out. We considered almost 50 tissues (48), building one GRN for each age interval ([20–29], [30–39], …, [70–79]).

We applied the MAGE algorithm to all tissues for both MAP$^{+}$ and MAP$^{-}$ to identify gene sets exhibiting consistently increasing or decreasing regulatory influence with aging. For the baseline analyses presented in Figs 3 and 4, MAGE was executed 10 independent times for each tissue and optimization objective. The resulting Age Index values were summarized using the arithmetic mean and sample standard deviation across the 10 runs.

**Figure 3:**
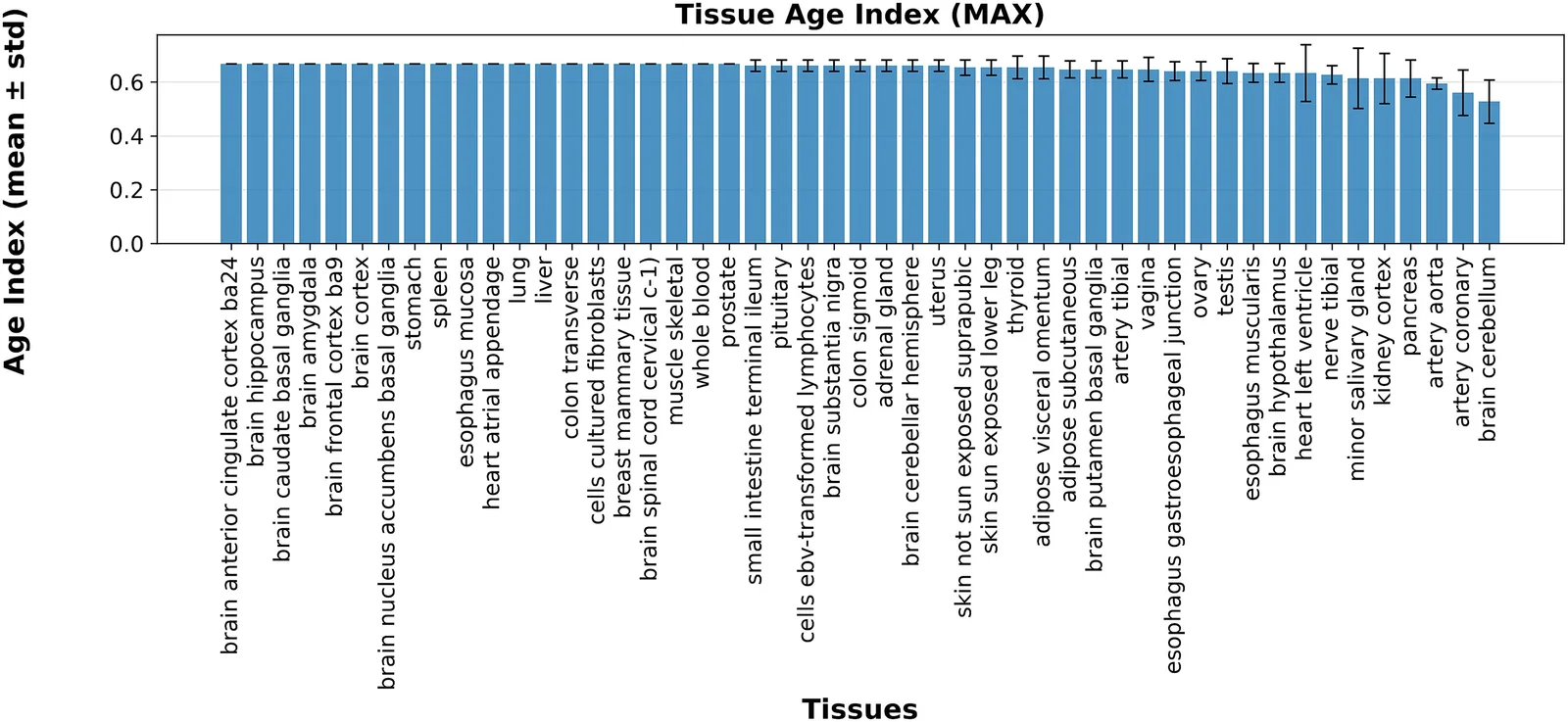
Maximum Age Indexes found per tissue (*λ =0.3*, *β> 0*, *ε =0.005*). Bar plot of mean maximum Age Index values computed for all tissues using the MAGE framework. Gene expression data from GTEx and regulatory interactions from TRRUST were integrated to construct age- and tissue-specific GRN sequences. For each tissue, MAGE was executed 10 independent times under the selected parameter configuration; the bars represent the mean and error bars indicate *± * one sample standard deviation across these runs. The maximization setting (MAP$^{+}$) highlights tissues in which the aggregated regulatory influence of the selected gene sets consistently increases with age.

**Figure 4:**
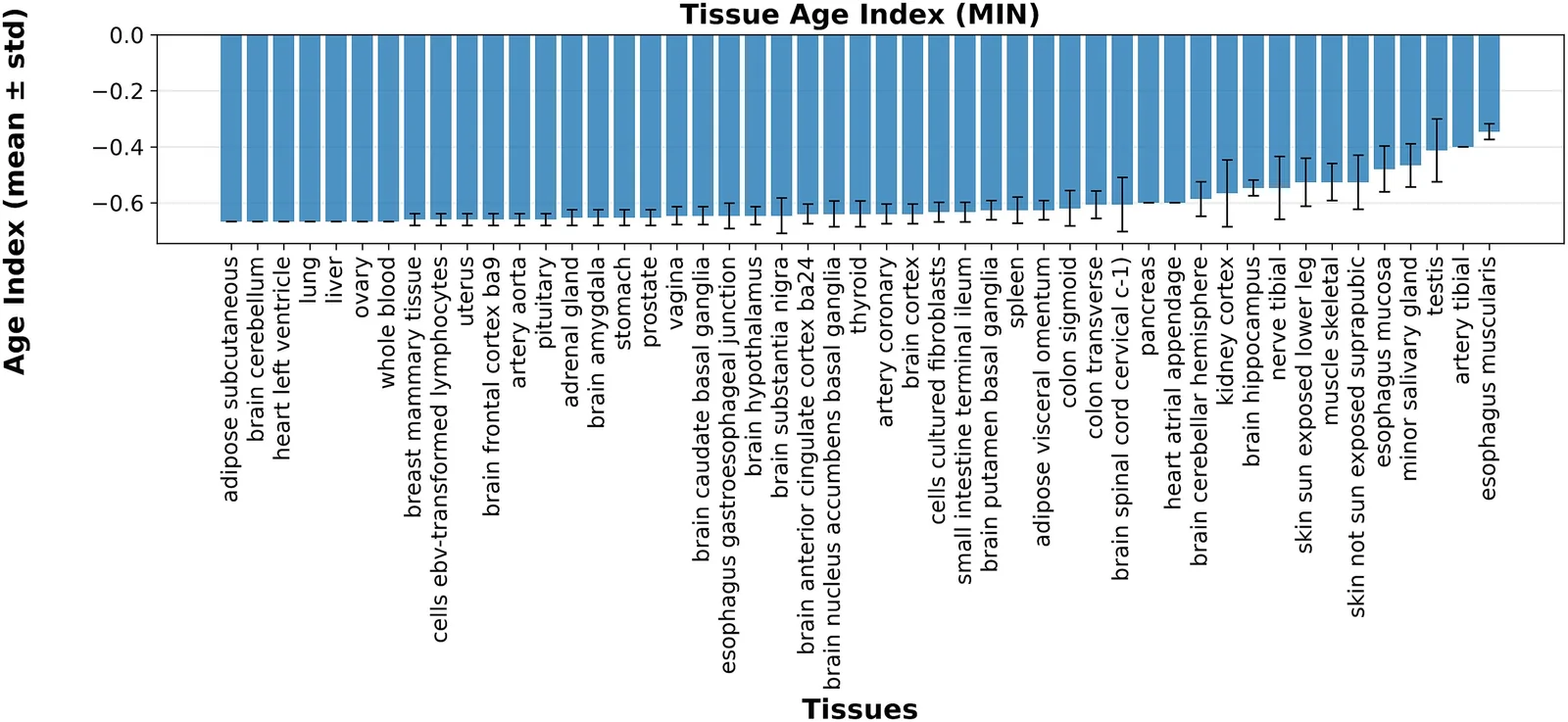
Minimum Age Indexes found per tissue (*λ =0.3*, *β> 0*, *ε =0.005*). Bar plot of mean minimum Age Index values computed across all tissues using the MAGE framework. Age-dependent GRNs were derived by combining GTEx expression profiles with TRRUST regulatory interactions. For each tissue, the algorithm was run 10 times independently; the bars represent the mean and error bars indicate *± * one sample standard deviation across these runs.

Figures 3 and 4 present the mean maximum and minimum Age Index values, respectively, across the analyzed tissues. The bars represent the arithmetic mean, and error bars denote *± * one sample standard deviation across 10 independent stochastic runs.

To evaluate the robustness of the Age Index under different stochastic runs and network perturbations, we performed additional experiments across multiple repetitions, noise levels, and perturbation strategies. The complete perturbation analyses are provided in the Supplementary Materials (see Supplementary Figures SM. 37–SM. 66).

To evaluate stochastic robustness, we analyzed the Age Index on the liver tissue under increasing structural perturbation levels ($p \in \{1,2,4,5,10\}\%$), considering *rewire*, *insert*, and *delete* operations alongside the unperturbed baseline network. For each configuration, the metric was computed over 10 independent stochastic runs and summarized as mean *± * 1 standard deviation. As shown in Fig. 5, the MAX Age Index remains highly stable across perturbation levels, with only minor variability around the baseline value. Similarly, the MIN Age Index (Fig. 6) exhibits limited deviation under increasing noise, and the dispersion across runs remains bounded. These findings indicate that the proposed Age Index is robust to moderate structural perturbations and is not driven by incidental stochastic fluctuations.

**Figure 5:**
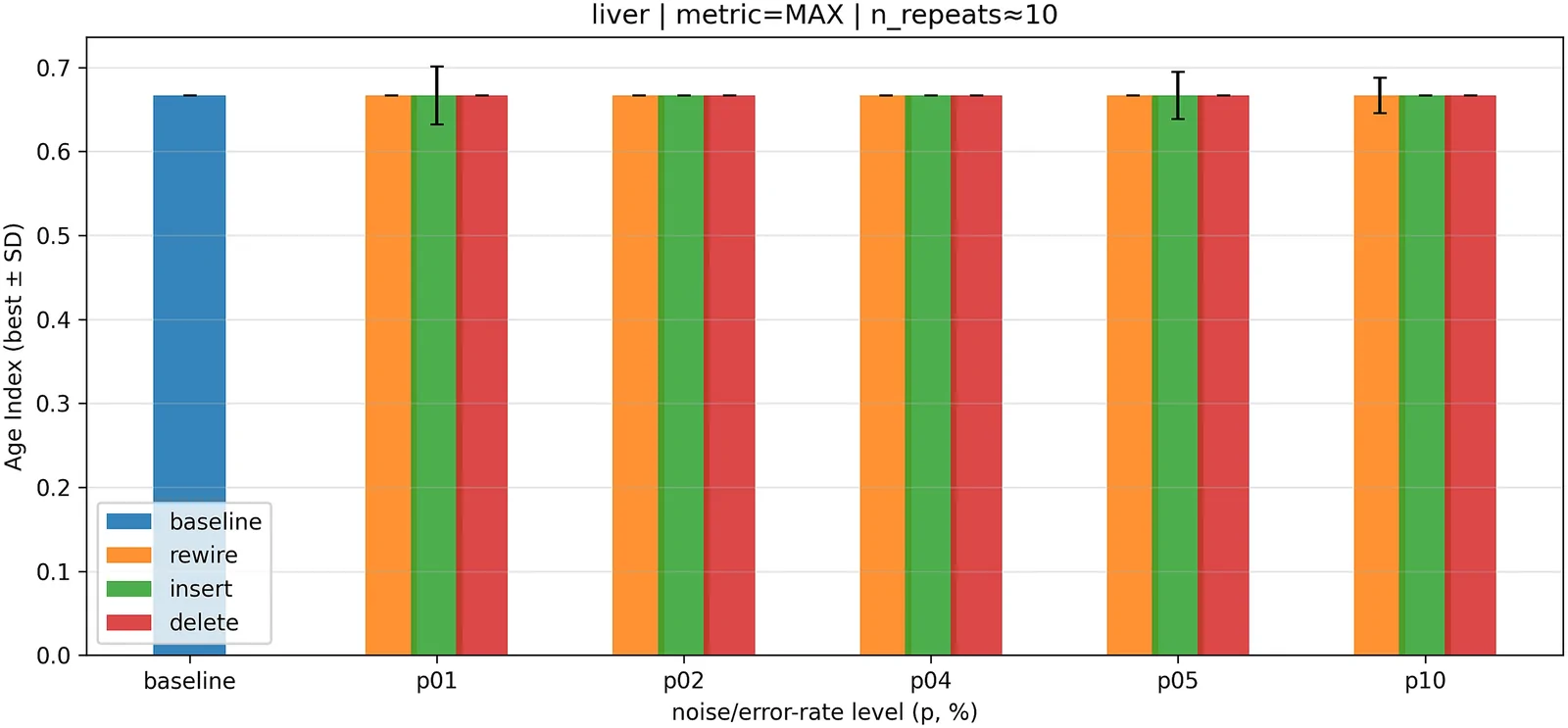
Stability analysis of the Age Index (MAX) for the most stable tissue across increasing perturbation levels ($p \in \{0,1,2,4,5,10\}\%$; *n=10* runs per level). Error bars denote *± 1* standard deviation, demonstrating limited stochastic sensitivity under network perturbation.

**Figure 6:**
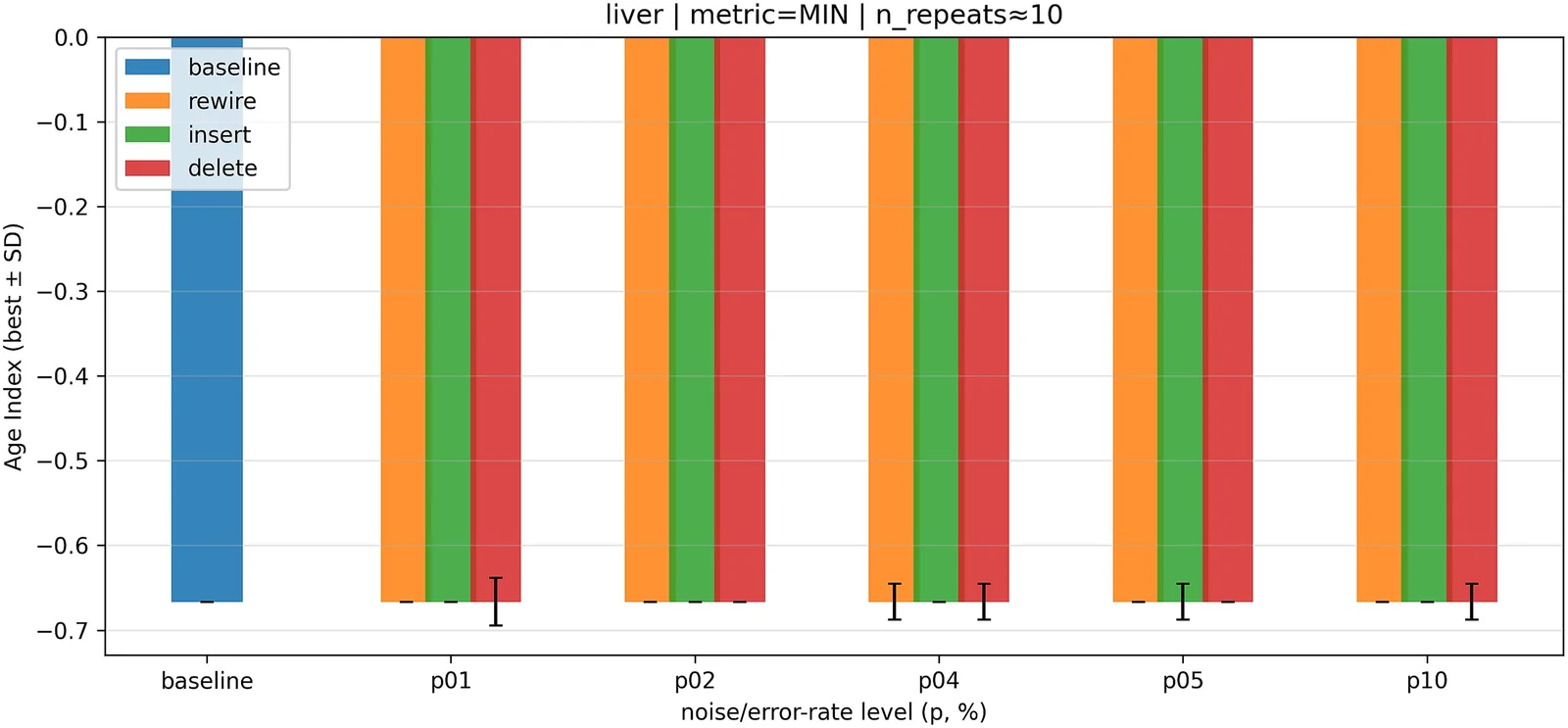
Stability analysis of the Age Index (MIN) for the most stable tissue across increasing perturbation levels ($p \in \{0,1,2,4,5,10\}\%$; *n=10* runs per level). The error bars denote *± 1* standard deviation, demonstrating limited stochastic sensitivity under network perturbation.

In Figs 7 and 8, the heatmap visualizations illustrate how *Age Index* values vary depending on the selected parameter combinations. The analyses consider $\lambda \in \{0.5, 1.0, 2.0\}$, $\epsilon \in \{0.003, 0.005, 0.008\}$, and *β = 0* (deterministic) or *β> 0* (stochastic) settings, and comparatively present the distribution and sensitivity of Age Index values across tissues under maximum (*max*) and minimum (*min*) conditions. The results indicate that the chosen parameters effectively capture the impact of parameter variations on the Age Index, confirming that the selected parameter ranges are appropriate for the analysis. Detailed results for each individual parameter configuration can be found in the Supplementary Material ( SM. 36- SM. 61).

**Figure 7:**
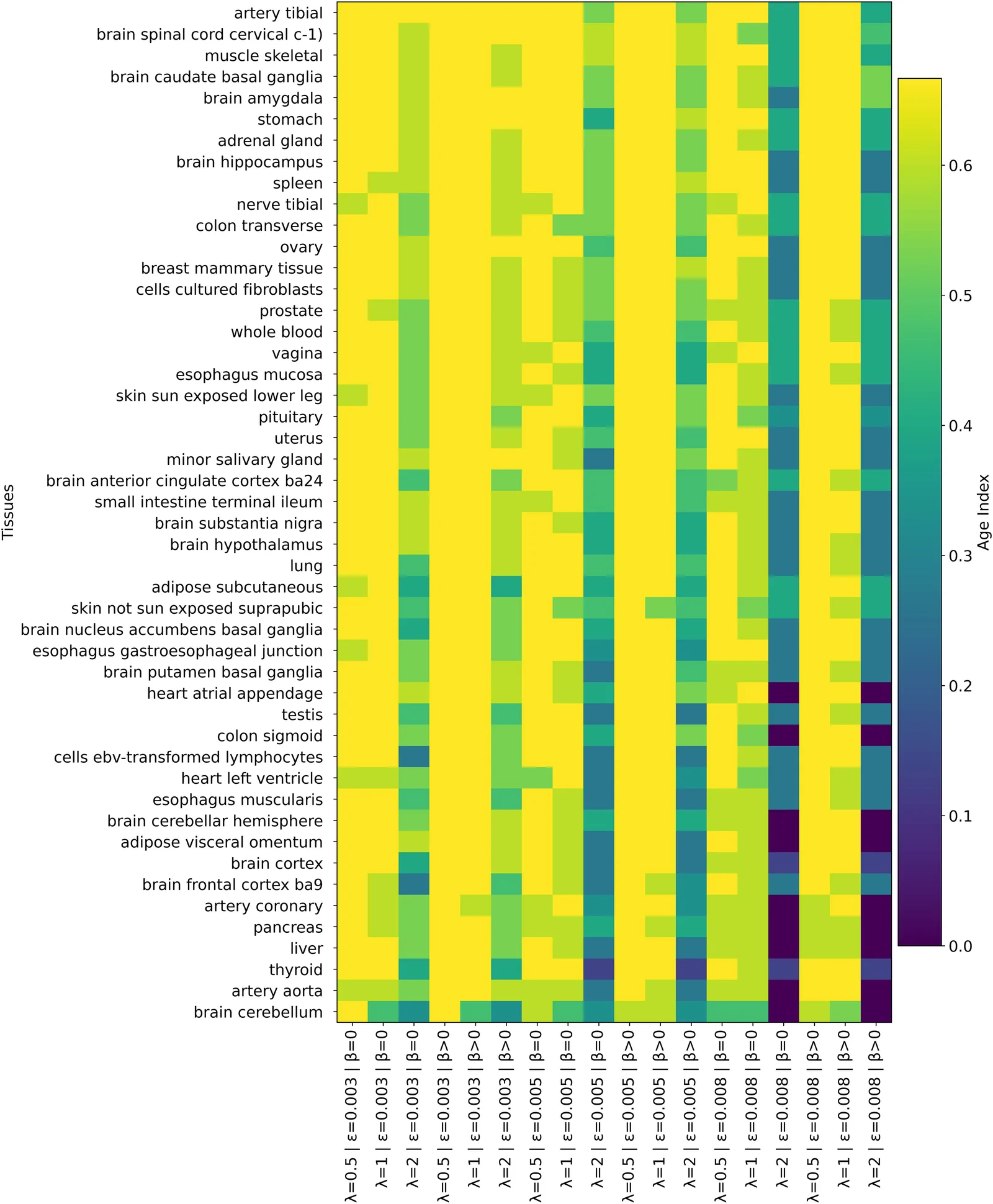
Tissue-specific maximum age index variations across *λ *, *ε *, and *β * configurations. Heatmap visualization of maximum Age Index values obtained for all tissues across different parameter combinations using the MAGE framework. Gene expression data from GTEx and transcriptional regulatory interactions from TRRUST were jointly used to construct tissue-specific GRN sequences. For each tissue and parameter configuration, MAGE was executed five times, and the best-performing Age Index value was retained. The heatmap provides a global view of how increasing age-associated regulatory influence patterns vary across tissues and parameter settings, enabling assessment of robustness and sensitivity.

**Figure 8:**
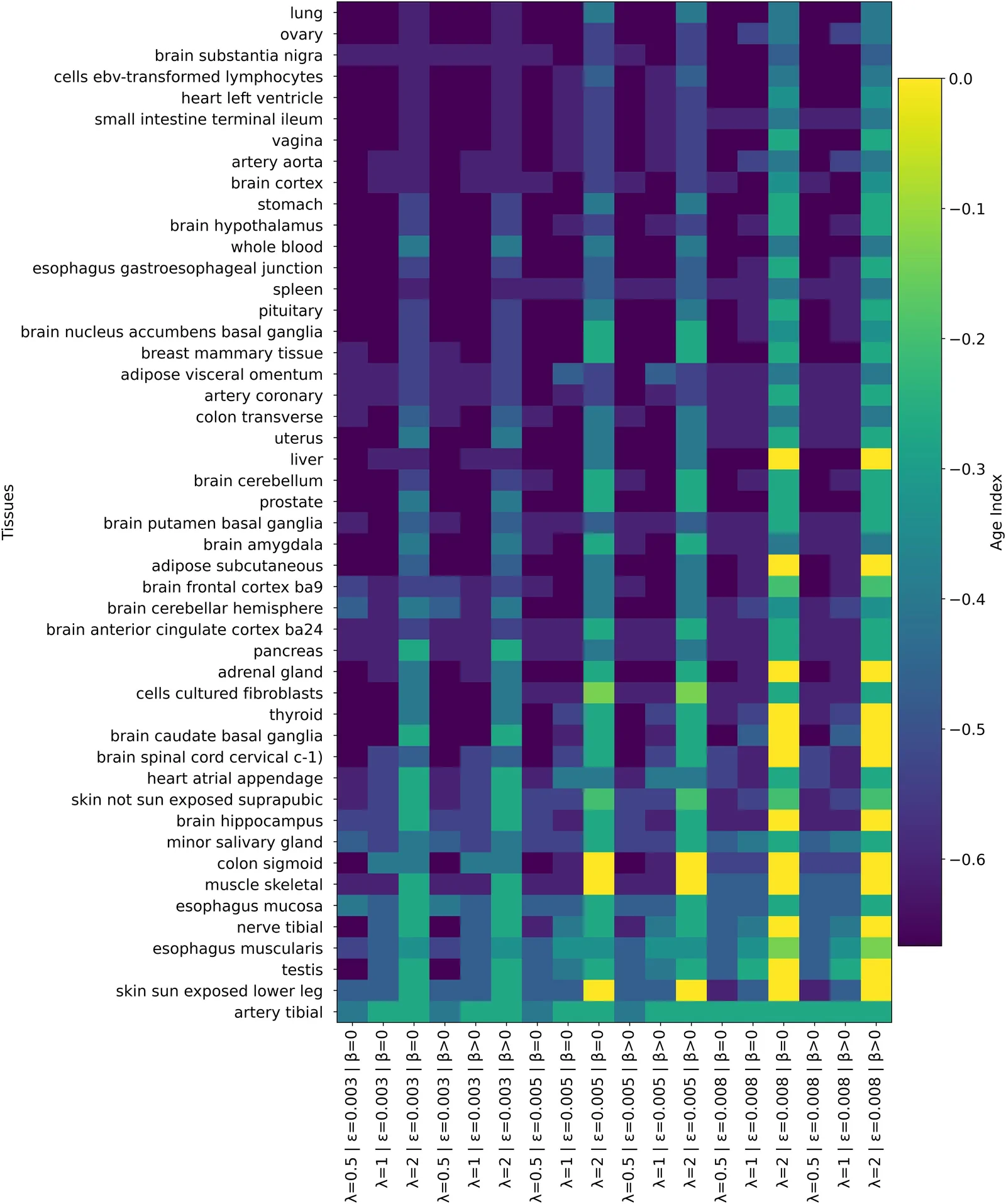
Tissue-specific minimum age index variations across *λ *, *ε *, and *β * configurations. Heatmap visualization of minimum Age Index values computed for all tissues across different parameter combinations using the MAGE framework. The analysis integrates GTEx-based gene expression profiles with TRRUST-derived regulatory interactions to model age-dependent GRNs. For each tissue and parameter setting, five independent runs of MAGE were performed, and the best-performing result was selected. This representation captures how consistently decreasing age-associated regulatory influence varies across tissues and parameter configurations, highlighting tissue-specific sensitivity patterns.

In the current study, the primary experiments presented in the main manuscript were conducted using the parameter configuration *λ = 0.3* and *ε = 0.005* under stochastic conditions. In addition to this baseline setup, supplementary experiments were performed to systematically examine the sensitivity of the method to variations in the underlying parameters. Accordingly, all combinations of $\lambda \in \{0.5, 1.0, 2.0\}$, $\epsilon \in \{0.003, 0.005, 0.008\}$, and both deterministic (*β = 0*) and stochastic (*β> 0*) settings were evaluated. Furthermore, the analysis was carried out separately for both the MAP$^{+}$ (increasing age-related influence) and MAP$^{-}$ (decreasing age-related influence) formulations of the age-index computation. The purpose of these extended experiments is to provide a comprehensive understanding of how network density, threshold levels, stochasticity, and the directionality of age-dependent influence (MAP$^{+}$ versus MAP$^{-}$) shape the resulting age-index patterns, thereby validating the robustness and consistency of the proposed approach across diverse parameter settings.

Figure 9 shows that when *ɛ * is held constant, increasing *λ * results in a broader spread of Age Index values across tissues, indicating greater variability in the magnitude of increasing influence patterns. Similarly, the stochastic setting of MAGE (*β> 0*) produces a higher concentration of tissues with larger Age Index values, reflecting its tendency to identify gene sets with stronger upward age-related trends. When *λ * is fixed and *ɛ * is varied downward across rows, the distribution of Age Index values changes noticeably, as higher *ɛ * thresholds filter out weaker trends and retain only strongly increasing trajectories. Overall, the stochastic structure of MAGE remains stable, consistently yielding robust maximization (MAP$^{+}$) results across parameter combinations.

**Figure 9:**
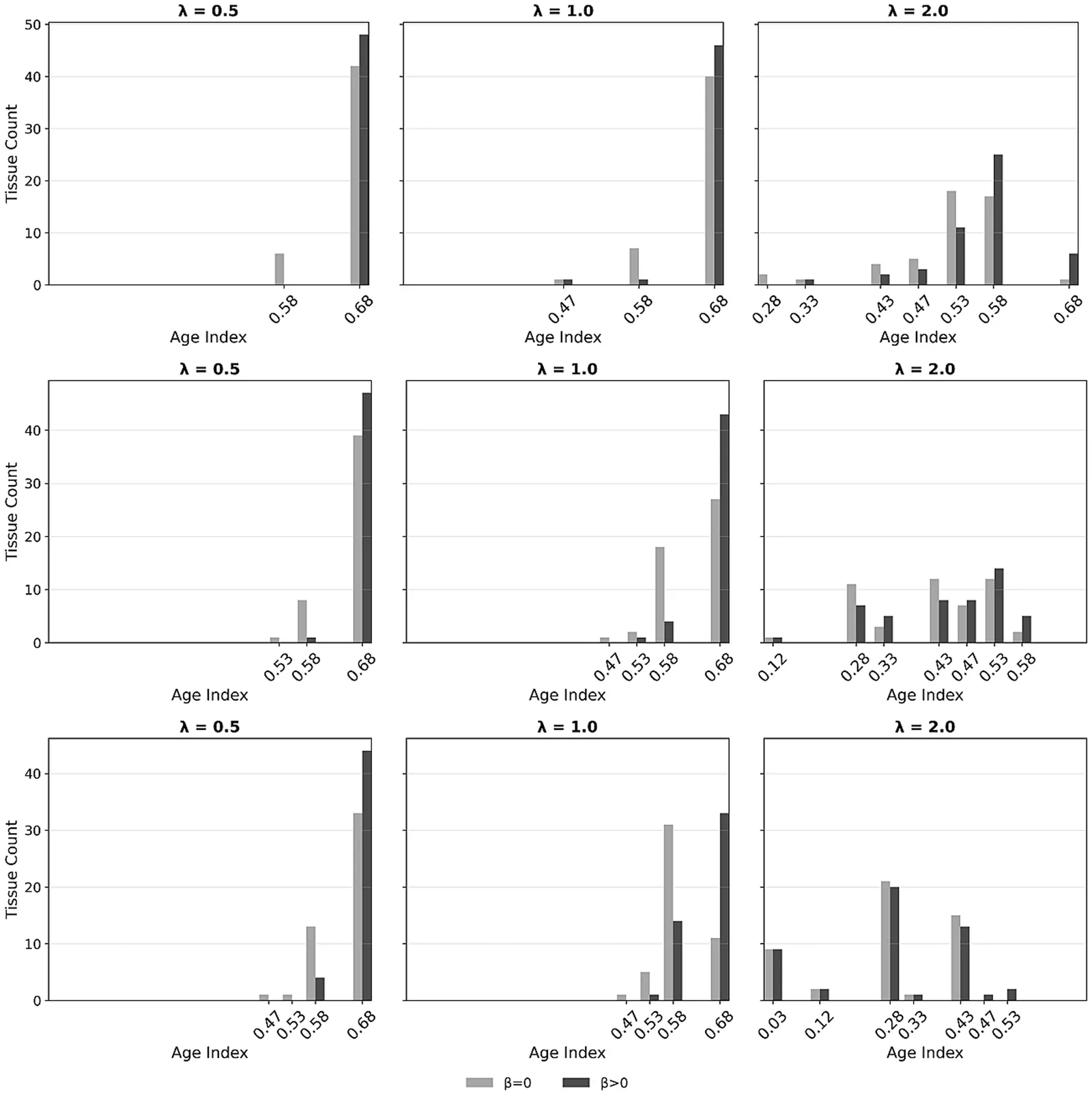
The figure shows MAP$^{+}$ (MAGE maximization) results across different *ɛ * and *λ * settings. The rows correspond to *ɛ * values (0.003, 0.005, 0.008 from top to bottom), and columns correspond to *λ * values (0.5, 1.0, 2.0 from left to right). Each graphic compares deterministic and stochastic selection: gray bars represent *β = 0* and black bars represent *β> 0*. These graphics demonstrate how increasing *ɛ * filters out minor fluctuations in influence trajectories and retains only consistent age-related trends defined by the Age Index, while variation across *λ * reflects the influence decay parameter’s role in controlling how rapidly regulatory effects diminish in the network.

Figure 10 shows that when *ɛ * is held constant, increasing *λ * leads to a wider spread of Age Index values in the minimization setting, indicating greater variability in declining influence patterns across tissues. The deterministic (*β = 0*) and stochastic (*β> 0*) versions of MAGE produce highly similar results, demonstrating the stability of the algorithm under both selection modes. When *λ * is fixed and *ɛ * increases downward across rows, only a small change is observed in the clustering of tissue Age Index values, suggesting that *ɛ * has a more limited filtering effect in the minimization case compared with maximization.

**Figure 10:**
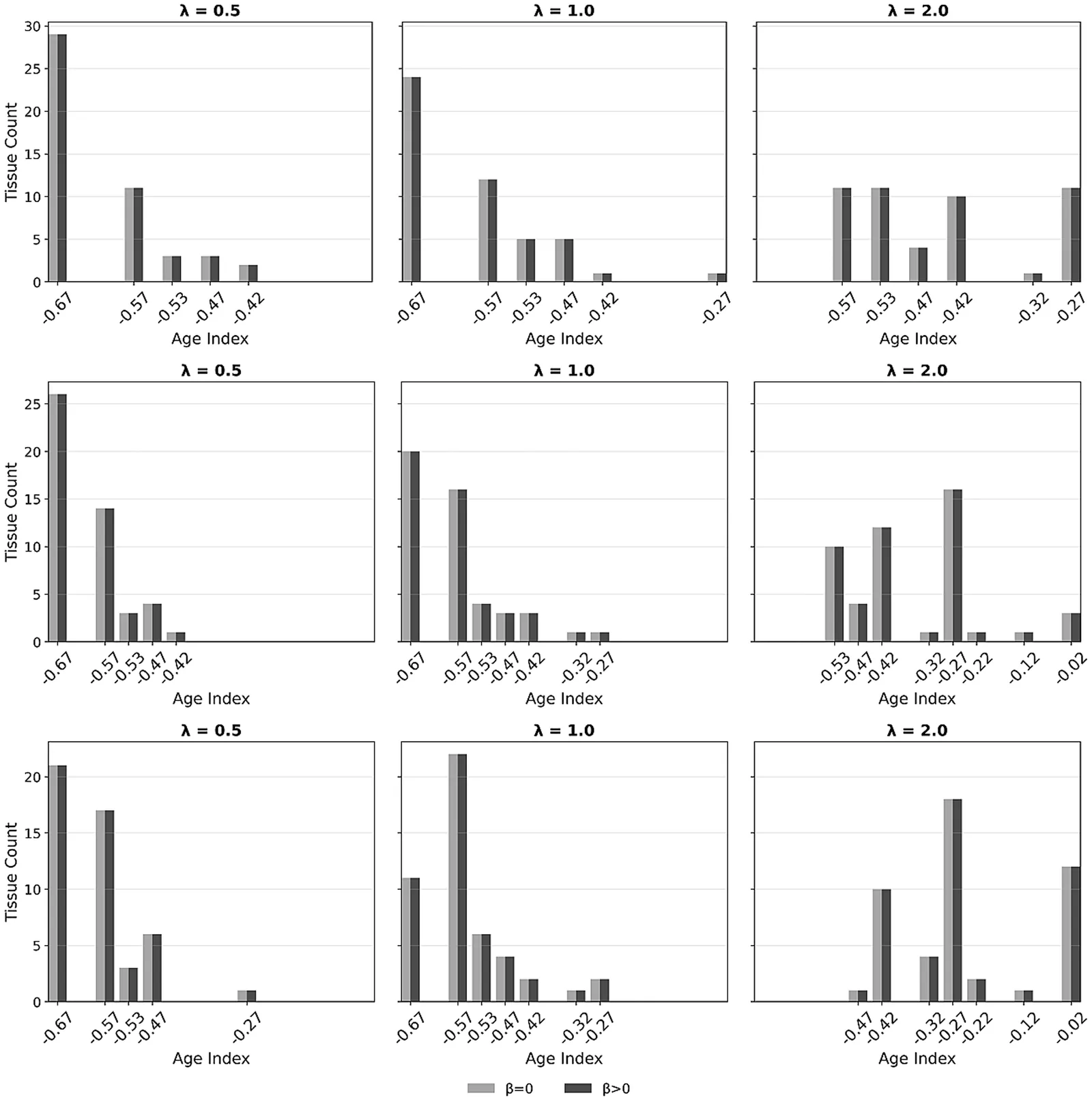
The figure shows MAP$^{-}$ (MAGE minimization) results across different *ɛ * and *λ * settings. The rows correspond to *ɛ * values (0.003, 0.005, 0.008 from top to bottom), and the columns correspond to *λ * values (0.5, 1.0, 2.0 from left to right). Each graphic compares deterministic and stochastic selection: the gray bars represent *β = 0* and the black bars represent *β> 0*. These graphics illustrate how increasing *ɛ * suppresses minor fluctuations and retains only strong and consistent declining influence patterns captured by the Age Index, while variation across *λ * reflects the influence decay parameter’s effect on how rapidly regulatory impact diminishes across the network.

Figures 11 and 12 demonstrate the influence of the *λ * parameter on the Age Index. The results show that smaller *λ * values produce denser networks, which increases motif influence and consequently yields higher Age Index values. In particular, the plane corresponding to *λ = 0.5* exhibits the highest age-index values across most tissues, while the transition from *λ = 0.5* to *λ = 1.0* leads to only minimal changes for the majority of tissues. This indicates that the Age Index remains highly stable within the interval *0.5*–*1.0*. In contrast, for *λ = 2.0*, the age-index values decrease substantially, reflecting the reduced network density and the resulting loss of structural influence. This behavior confirms that the age-index metric is sensitive to network sparsity in a manner consistent with biological expectations. Furthermore, the visualizations reveal that while some tissues remain largely unaffected by changes in *λ *, others exhibit pronounced shifts, particularly when moving from *λ = 0.5* to *λ = 2.0*, highlighting tissue-specific sensitivity. The fact that both deterministic (*β = 0*) and stochastic (*β> 0*) formulations preserve the same global trend further supports the robustness and consistency of the method. Overall, these findings demonstrate that *λ * strongly shapes age-index behavior, with lower *λ * values producing more biologically meaningful and informative results.

**Figure 11:**
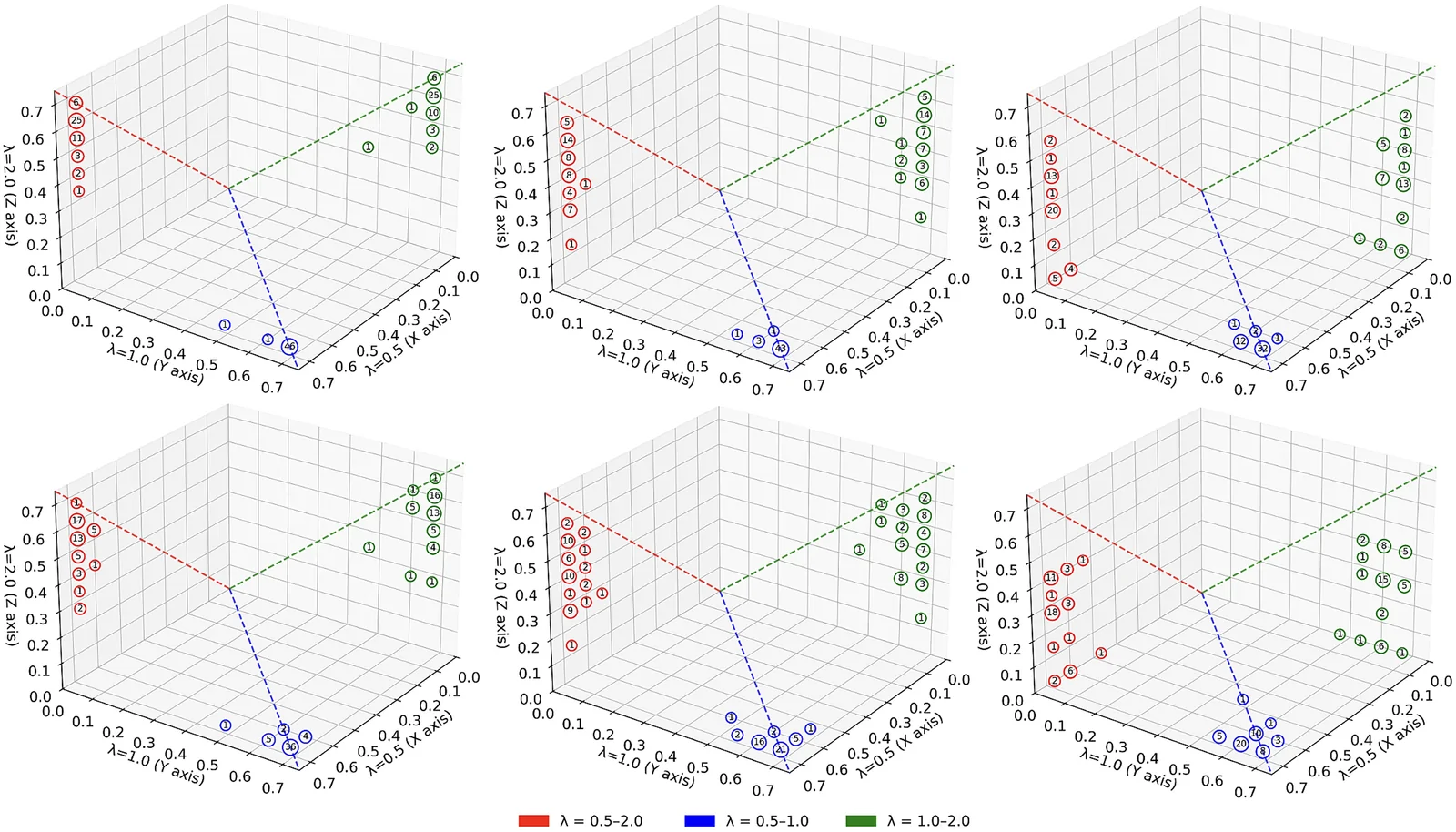
These graphics present the MAP+ results of MAGE using a 3D visualization across different *ɛ * and *β * settings. Along the horizontal direction, *ɛ * increases from left to right (0.003, 0.005, 0.008). The top row displays the stochastic configuration (*β> 0*), while the bottom row shows the deterministic configuration (*β = 0*). Each 3D graphic illustrates, for pairs of *λ * values, the number of tissues that share the same Age Index value, plotted at the points where the corresponding *λ * axes intersect.

**Figure 12:**
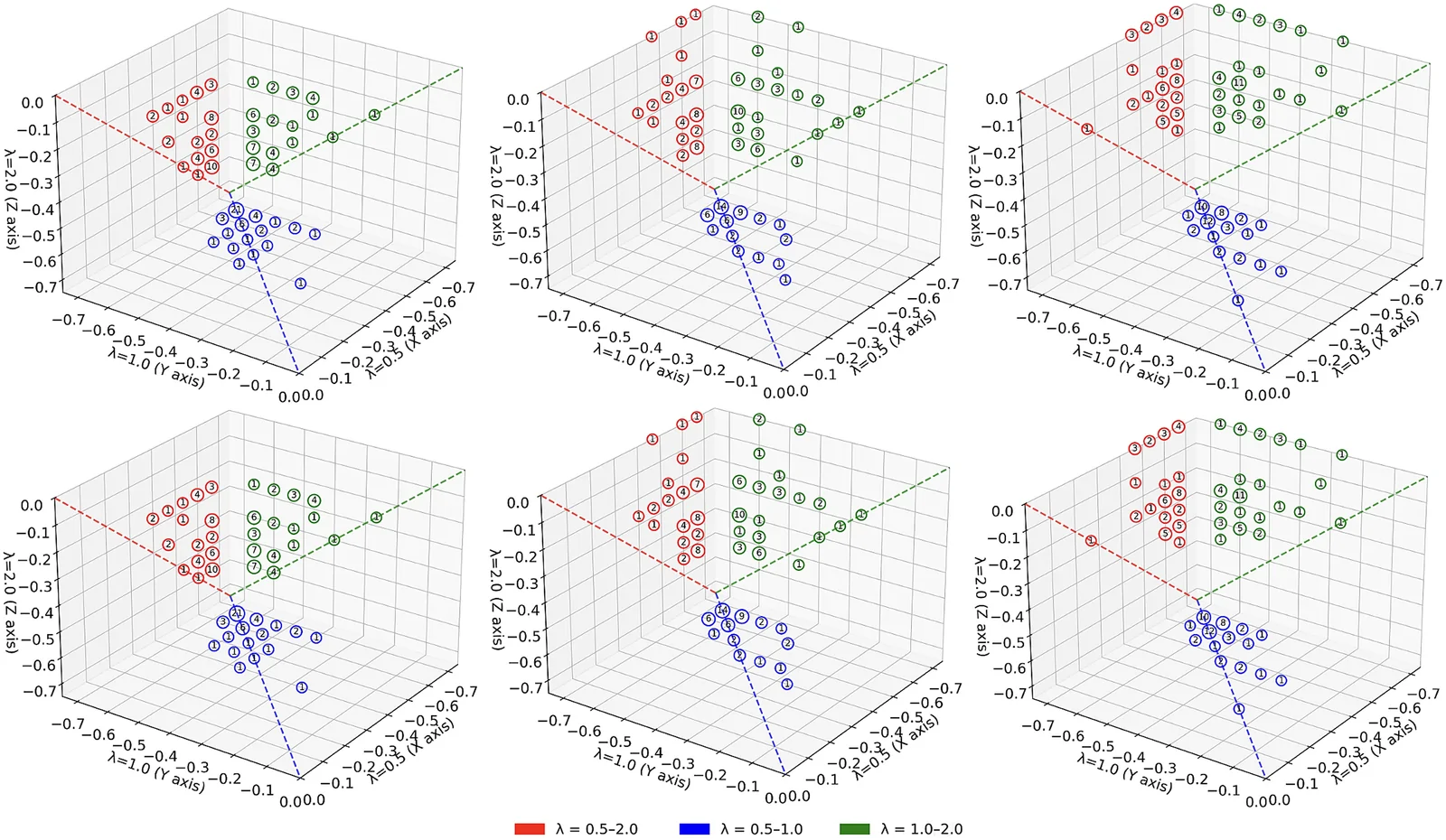
These graphics present the MAP- results of MAGE using a 3D visualization across different *ɛ * and *β * settings. Along the horizontal direction, *ɛ * increases from left to right (0.003, 0.005, 0.008). The top row displays the stochastic configuration (*β> 0*), while the bottom row shows the deterministic configuration (*β = 0*). Each 3D graphic illustrates, for pairs of *λ * values, the number of tissues that share the same Age Index value, plotted at the points where the corresponding *λ * axes intersect.

In summary, the minimum and maximum Age Index results provide a complementary perspective on the transcriptional landscape from the perspective of aging across different tissues. The MAGE algorithm’s effectiveness in isolating both consistently increasing downregulated, and upregulated gene sets demonstrates its discriminative power for the MAP+/- problems.

**Evaluation of the MAGE statistical significance.** We test whether if the MAGE algorithm can find gene sets with large magnitude Age Index values. We aim to answer whether MAGE can actually find a good solution to the NP-hard MAP+ and MAP- problems at the human genome scale. MAGE is indeed a stochastic heuristic method. The NP-hardness of the MAP+/- problems makes it impossible to verify the optimality of the results. The quality of the MAGE results is evaluated using statistical significance measures. To do that, the Z-scores of MAGE results are evaluated as follows. Given an instance of a MAP$^{+}$ problem over the set of genes *V*, let us denote the gene set found by MAGE and the Age Index value of this gene set with *S ⊆ V* and *α *, respectively. We randomly generate *n = * 100 random solutions by randomly choosing a subset of *|S|* genes from *V* (i.e. the same number of genes as MAGE identified). Let us denote the Age Index of the *i*th random set with $\alpha _{i}$. We denote the mean and the standard deviation of these *n* samples with *μ * and *σ *, respectively. We compute the Z-score of MAGE as $\frac{\alpha - \mu }{\sigma }$. We compute the Z-score of MAGE for the MAP- problem as $-\frac{\alpha - \mu }{\sigma }$ since this is the minimization version of the same problem. We consider that the gene set found by MAGE is statistically significant if the Z score is greater than 2, with significance increasing with the Z score.

We run MAGE on all 48 tissues and measure the Z score of MAGE for each tissue for both MAP+ and MAP-. Figure 13 (left) presents the results for MAP+. We observe that MAGE identifies statistically significant gene sets (i.e. Z score is greater than 2) for all tissues. Notice that, unlike the Age Index values, which only yield two outcomes (see Fig. 3), the Z scores of the 48 tissues span a wide range of values from around 3 to >17. This indicates that not all tissues have the same distribution of Age Index values to all the genes. MAGE yields the highest significance for thyroid, indicating extremely strong enrichment of aging-related gene expression signatures. For 12 tissues, MAGE yields Z-score values of 6 or more, and for 35 tissues the Z-score of MAGE is at least 4, which indicates a high statistical significance. These two Z-scores correspond to *P*-values of around $10^{-9}$ and $3\times 10^{-5}$.

**Figure 13:**
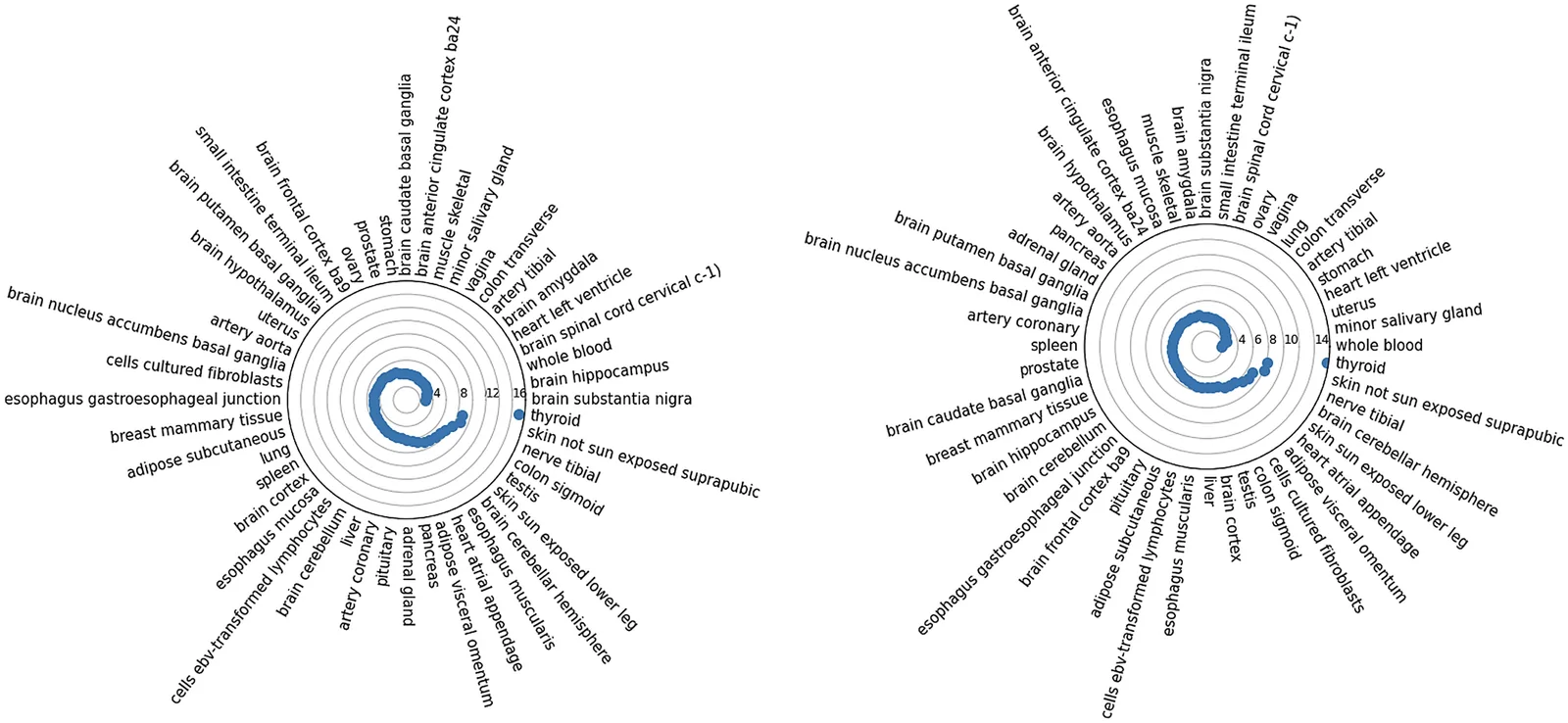
Left: Z score of the gene set identified by the MAGE algorithm for MAP+ (i.e. increasing influence with age) for 48 tissues sorted from the highest to lowest significance in clockwise direction. Right: The same result for the gene set found by MAGE for MAP- (i.e. diminishing influence with age).

Figure 13 (right) shows the Z-scores of MAGE for MAP$^{-}$ (declining influence with aging). Our results show that MAGE finds statistically significant gene sets for all the 48 tissues. Coupled with the results in Fig. 13 (left), this implies that, for each tissue, there are groups of genes that have consistent ascending influence while some other gene groups have declining influence with aging. Similar to MAP$^{+}$, MAGE results in an exceptionally high statistical significance with Z-score *> 14*. For six tissues, MAGE yields Z-score values of 6 or more, and for 31 tissues the Z score of MAGE is at least 4, which indicates a high statistical significance.

In summary, MAGE consistently returns statistically significant solutions to both MAP+ and MAP-. These prove the existence of ascending and declining influences of groups of genes in all tissues, yet these gene sets may be identical across different tissues. MAGE consistently produces the highest Z-scores for thyroid, skin, and nerve tibial tissues for both MAP$^{+}$ and MAP$^{-}$. If our MAGE algorithm had no discriminatory power, Z-scores would cluster around zero. Instead, the presence of large Z-score values with tissue specificity constitutes strong evidence that our algorithm selects gene clusters with nonrandom, biologically significant expression profiles. Our results underscore the tissue-specific nature of aging as the Z-scores vary even when the Age Index values are the same across different tissues.

### Qualitative evaluation

The biological significance of the experiments has been calculated to demonstrate the usefulness of the MAGE algorithm.

**Analysis of most significant aging tissues.** In Figs 3 and 4, the tight clustering of positive and negative Age Index values across tissues demonstrates the algorithm’s capacity for consistent and robust feature selection in identifying aging-associated gene networks. The upper-bound clustering of maximum values supports the reproducible detection of commonly upregulated genes related to aging in heterogeneous tissue types, consistent with prior findings that highlight shared transcriptional signatures of aging across multiple organs [24]. Similarly, the narrow distribution of minimum Age Index values indicates a conserved pattern of downregulation associated with aging suppression mechanisms such as mitochondrial decline, proteostasis loss, and reduced cell cycle activity [55]. These results support the existence of universal transcriptional programs that are reliably captured by the algorithm despite inter-tissue variability. Minor deviations or outliers likely reflect tissue-specific aging trajectories or differences in transcriptional responsiveness, but the overall convergence of both up- and down-regulated signatures underscores the biological relevance and generalizability of the identified gene clusters.

Figure 13 shows that the most distinct separation for both up-regulated and down-regulated genes occurs in the thyroid, skin not exposed to the sun, and nerve tibial tissues. This is consistent with the evidence in the literature; thyroid stimulating hormone levels tend to increase with aging [56], while triiodothyronine (T3) and peripheral conversion of thyroxine (T4) into T3 tend to decline with aging [57]. These age-related endocrine changes are likely to induce broad transcriptional shifts in thyroid-related metabolic and regulatory genes, explaining the detection of both up- and down-regulated genes in this tissue. Skin undergoes intrinsic aging independent of photoaging, which is characterized by a decline in collagen production, reduced epidermal turnover, and changes in the composition of the dermal extracellular matrix [58]. These changes involve complex molecular pathways including oxidative stress responses, inflammation, and cellular senescence [59], leading to both increased and decreased expression of numerous genes. Thus, the identification of significant gene regulation in both directions aligns with the multifactorial biology of intrinsic skin aging. Aging is known to affect peripheral nerves through mechanisms such as axonal degeneration, demyelination, impaired nerve regeneration, and altered neuromuscular junctions [60]. Moreover, age-related decline in mitochondrial function and oxidative phosphorylation in neurons contributes to transcriptional changes [5]. These processes cause both upregulation of stress and repair pathways and downregulation of genes involved in nerve conduction and growth, making the identification of bidirectional gene regulation in tibial nerve tissue biologically plausible.

**Tissue interaction network: aging perspective.** We leverage the gene sets identified to construct a tissue interaction network via the genes with high Age Index values. To do that, we map each tissue to the anatomical system it belongs to. Table SM. 1 lists the mapping of the 48 tissues to 10 anatomical systems. We construct the tissue interaction network as follows. We create a node for each anatomical system. We say that a gene is validated through PubMed if there exists a publication in the literature in PubMed with the keyword ”aging” and the gene name coexists. We also create a node for each gene that MAGE identifies as a solution to MAP$^{+}$ or MAP$^{-}$ for each tissue and that gene is validated through PubMed. We connect the node for each such gene with the node for an anatomical system with an undirected edge if that gene is selected by MAGE for a tissue belonging to that anatomical system. The network in Fig. 14 shows the resulting anatomical associations at the system-level. A network constructed solely from PubMed literature captures reported gene-anatomical system associations but lacks tissue-specific regulatory context and age-dependent influence dynamics. In contrast, a network derived exclusively from MAGE reflects data-driven regulatory influence patterns inferred from GRNs but remains independent of prior biological knowledge. The proposed gene–anatomical system network integrates MAGE-identified gene sets with PubMed-supported evidence, combining data-driven aging signals with literature-based validation. This integration enables the interpretation of age-dependent regulatory patterns while avoiding reliance on either literature co-occurrence alone or inferred regulatory influence in isolation. Each gene included in the visualization has previously been reported in aging studies and exhibits regulatory or expression relevance in one or more types of tissues grouped into broader physiological systems [61]. By mapping these genes to systems such as the nervous, immune, endocrine, and reproductive systems, the graph captures both system-specific and cross-system regulatory signatures. This representation highlights the pleiotropic nature of key aging markers and provides insight into how aging-associated genetic programs may coordinate across distinct biological domains. The graph serves as an interpretive framework to explore the shared transcriptional mechanisms underlying age-related processes in human tissues. Several genes, notably FOXA3 and FOXL2, demonstrate pleiotropic roles by influencing multiple physiological systems, such as the nervous, endocrine, and digestive systems, highlighting their potential importance in the aging process. FOXA3, for instance, is involved in metabolic regulation and energy homeostasis, while FOXL2 plays a critical role in endocrine and reproductive function [62]. These genes may act as intersystemic regulators, modulating aging-related phenotypes such as neuroendocrine signaling, metabolic balance, and immune system interactions. Their activities reflect the complex cross-talk among physiological systems that underlies age-related diseases, including neurodegeneration, metabolic syndrome, and hormonal dysregulation.

**Figure 14:**
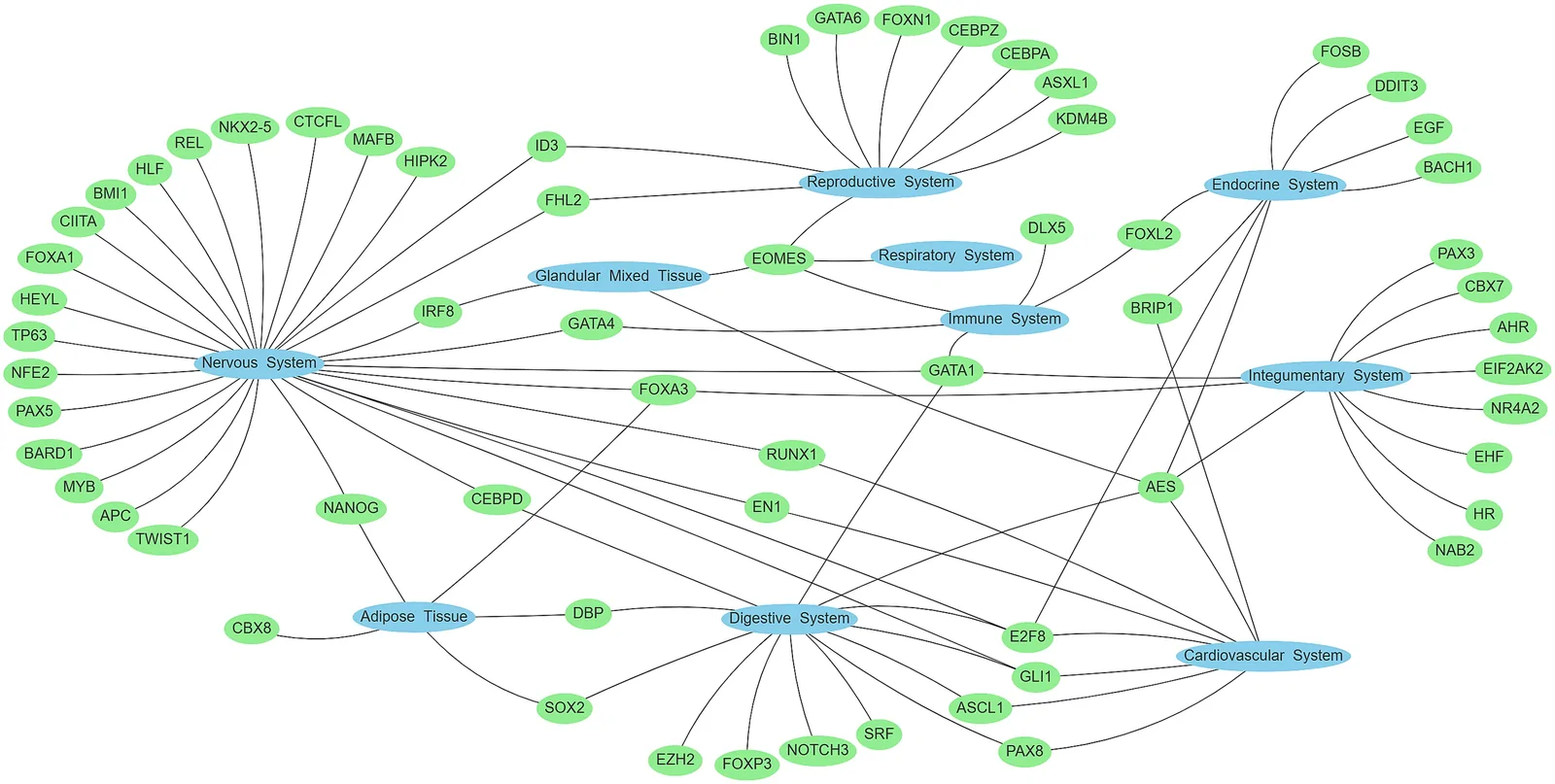
Gen-Anatomical System (related tissues grouped together) Bipartite Network (System-Based Placement and Free Node Positioning). This visualization shows a bipartite network structure representing the distribution of genes associated with aging according to different anatomical systems. The blue nodes in the graph represent anatomical systems, while the green nodes represent genes selected by the MAGE algorithm and identified in the PubMed literature as being associated with age.

Clarification of tissue-specific gene sets. In this analysis, gene sets are identified in a tissue-specific manner. For each tissue, MAGE selects the top-ranked gene sets under both increasing (MAP$^{+}$) and decreasing (MAP$^{-}$) age-index regimes. These tissue-specific gene sets are then used as nodes in the tissue interaction network, allowing comparison of aging-related regulatory patterns across tissues rather than aggregating genes from all tissues into a single global set.

**Analysis of tissue aging similarity.** Next, we answer the question: how similar are tissues to each other in terms of the aging-associated genes. To do that, for each tissue pair, we compute the Jaccard coefficient of the gene sets MAGE identifies for them. The Jaccard coefficient was used to quantify the overlap between gene sets identified by MAGE across different tissues. Specifically, it measures the similarity of MAP$^{+}$ and MAP$^{-}$ gene sets between tissue pairs, allowing us to assess the extent to which aging-related regulatory programs are shared or tissue-specific. We observed generally low to moderate Jaccard similarity across tissues, indicating that while certain aging-associated genes recur across multiple tissues, the majority of regulatory influence patterns remain tissue-specific. This observation supports the motivation for a tissue-aware aging analysis rather than a single global gene set. Figure 15 shows the distribution of the Jaccard coefficients for each tissue with the rest of the tissues. We observe that most tissues exhibit high MAGE scores, predominantly above 0.90, suggesting widespread and robust age-related transcriptional regulation. Tissues such as *prostate*, *thyroid*, and *brain cortex* display narrow and dense distributions, indicating a consistent and homogeneous age effect across samples. Conversely, tissues like *brain substantia nigra*, *skin not sun exposed suprapubic*, and *esophagus gastroesophageal junction* exhibit broader violin shapes, reflecting greater inter-individual variability in age-associated gene expression. A few tissues, including *uterus*, *vagina*, and *stomach*, show slightly lower medians, suggesting potentially reduced or variable age influence in those contexts. These patterns provide insight into the tissue-specific nature of transcriptional aging and support the robustness of MAGE as a comparative metric.

**Figure 15:**
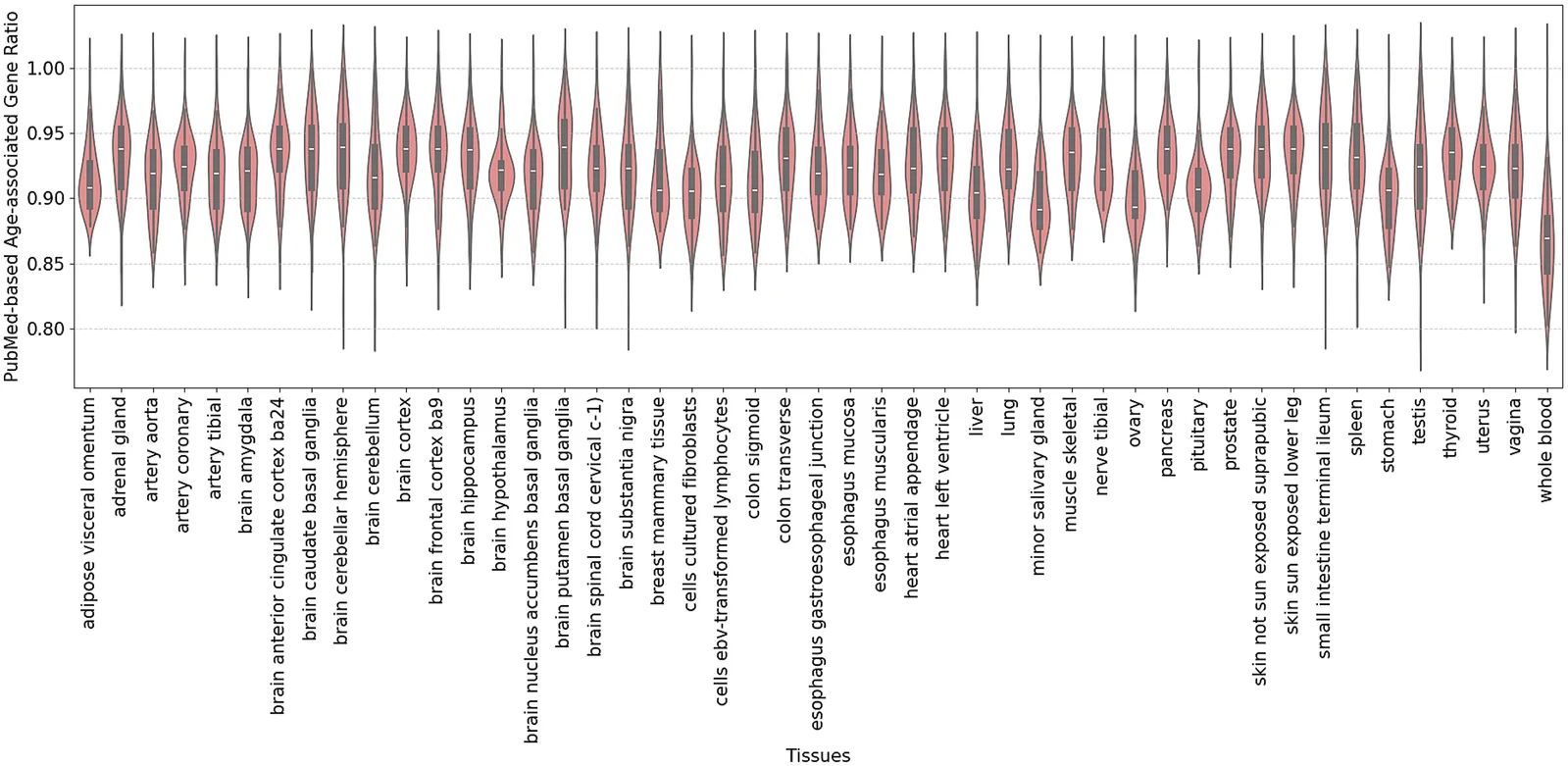
MAGE score distributions across tissues based on PubMed-verified aging-related genes. The violin plots indicate the variability and density of age-associated regulatory scores for each tissue.

**Gene enrichment analysis.**  Figure 16 shows the results of the Gene Ontology Biological Process (GO:BP) enrichment analysis applied to 33 aging-related genes selected by the *MAGE* algorithm and validated via PubMed (Only from clusters with Age-Associated genes *> 1* and Age-Not-Associated genes *= 0*). The analysis was performed using g:Profiler with a significance threshold of $P_{\textrm{adj}}<.05$ (g:SCS correction), and redundant hierarchical GO terms were excluded for clarity. The most significant enrichment was observed for *regulation of transcription by RNA polymerase II* ($P_{\textrm{adj}} = 2.38 \times 10^{-21}$), suggesting a central role for transcriptional control in aging. Other enriched terms, including *cellular response to chemical stimulus* and *mononuclear cell differentiation*, point to the involvement of stress response and immune processes. These results are visualized in Fig. 16 and summarized in Table 1. The findings align with existing literature. For instance, RNA Pol II elongation speed has been shown to increase with age, altering transcriptional regulation in human and animal tissues [63, 64]. Additionally, the roles of environmental stress and immune system dynamics in aging are well documented [65, 66]. Overall, these results indicate that MAGE effectively captures genes with functional relevance to key biological pathways of aging.

**Table 1:** Top enriched GO:BP terms among MAGE-selected genes.

Term ID	Term name	$P_{\textrm{adj}}$ (query_1)
GO:0006357	regulation of transcription by RNA polymerase II	$2.384 \times 10^{-21}$
GO:0070887	cellular response to chemical stimulus	$6.343 \times 10^{-5}$
GO:1903131	mononuclear cell differentiation	$2.567 \times 10^{-2}$

**Figure 16:**
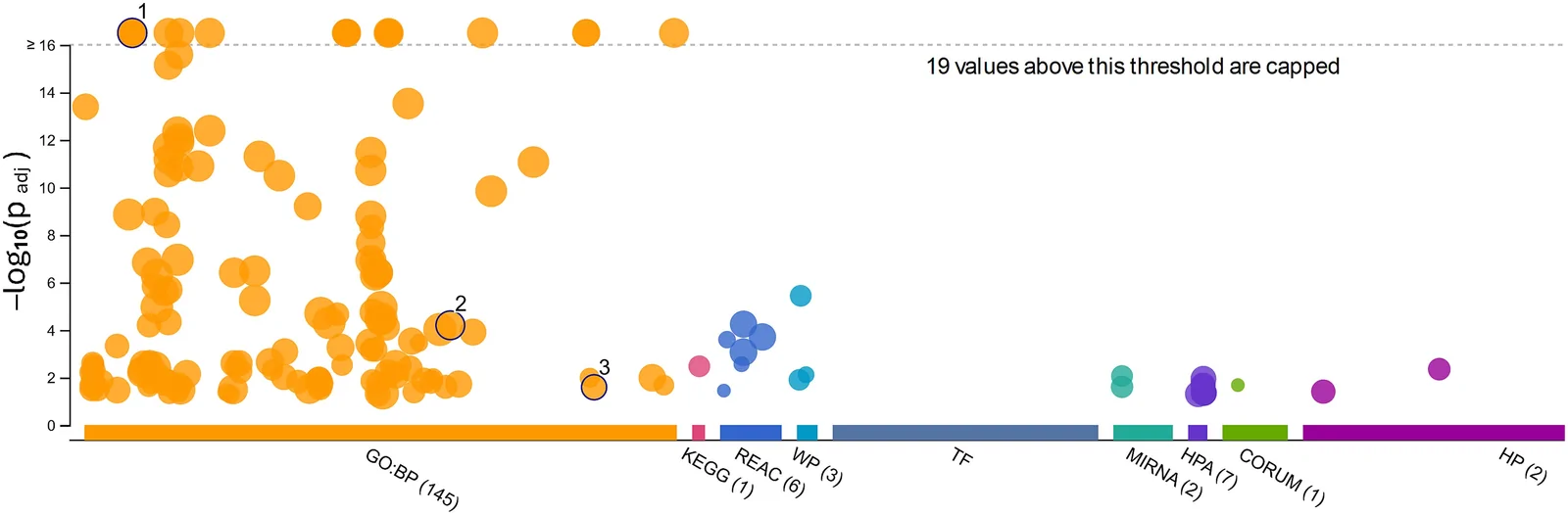
GO:BP enrichment results visualized for 33 aging-related genes selected by MAGE. Bubble size reflects the number of genes associated with each term, while color indicates statistical significance ($-\log _{10} \, p$).

The MAGE algorithm demonstrates strong biological significance through its consistent identification of aging-related gene clusters across a wide range of human tissues. Moreover, the biological significance of the MAGE algorithm is supported by multiple layers of evidence spanning tissues, gene functions, and physiological systems. The consistent clustering of both maximum and minimum Age Index values highlights the algorithm’s ability to robustly identify universally up- and down-regulated gene networks associated with aging. These gene expression patterns align with well-established aging mechanisms such as mitochondrial decline, proteostasis disruption, and cell cycle attenuation. Tissues like thyroid, skin (not sun exposed), and tibial nerve showed the most distinct gene regulation profiles, in agreement with known biological processes including endocrine dysregulation, intrinsic skin aging, and neurodegeneration. A system-level bipartite analysis further confirmed that selected genes are functionally involved in major physiological systems, such as the nervous, endocrine, immune, and reproductive systems, often playing pleiotropic roles. GO enrichment analysis validated that these genes are significantly involved in core aging processes like transcriptional regulation by RNA polymerase II, immune modulation, and stress response pathways [67]. The high and consistent MAGE scores across most tissues, combined with biologically interpretable inter-tissue variability, support MAGE as a reliable and biologically meaningful tool for uncovering conserved and tissue-specific transcriptional programs of aging.

### Comparison with existing aging analysis approaches

Existing computational studies of aging primarily fall into three categories: age-correlated differential expression analyses, biological aging clocks, and static network-based approaches. Differential expression methods identify genes whose expression levels correlate with age but treat genes independently and do not account for regulatory interactions or coordinated influence patterns. Aging clocks aim to predict chronological or biological age with high accuracy, often at the expense of mechanistic interpretability. Network-based aging studies typically analyze static topological properties, such as hub genes or pathway enrichment, without explicitly modeling how regulatory influence evolves across age intervals.

MAGE addresses a complementary objective by formulating aging as a time-dependent network influence optimization problem. Rather than focusing on individual gene–age correlations or age prediction accuracy, MAGE identifies gene sets whose aggregated regulatory influence consistently increases or decreases with age. This distinction makes direct benchmarking against predictive aging clocks or gene-centric differential expression methods nontrivial, as these approaches optimize fundamentally different objectives. Nevertheless, MAGE can be viewed as complementary to existing methods by providing mechanistic, network-level insight into age-associated regulatory dynamics that are not captured by static or predictive frameworks.

Although MAGE is conceptually distinct from traditional aging gene identification frameworks, it remains important to examine whether the genes selected by MAGE exhibit external consistency with previously reported aging-associated gene sets. Such a comparison does not aim to establish methodological equivalence, but rather to assess biological concordance across independently derived aging signatures. By evaluating the overlap between MAGE-derived gene sets and genes identified through expression-based and cohort-level aging studies, we provide an additional layer of validation supporting the biological plausibility of the proposed network-based influence formulation.

To evaluate external consistency, we compared the MAGE-selected unique gene sets with the 509 aging-related genes reported by Yang *et al*. [68] in the *Postgraduate Medical Journal* (PMJ) and the 130 genes with rank *R≥ 6* reported by Coler-Reilly *et al*. [69] in *Aging Cell* (ACEL). For this comparison, gene symbols from the final tissue-specific MAGE solutions under the baseline parameter configuration were pooled and deduplicated separately for the MAGE MAX and MAGE MIN sets. All intersections were subsequently calculated at the unique gene-symbol level.

As summarized in Table 2, MAGE MAX and MAGE MIN shared five and two genes with the PMJ gene set, respectively. Their corresponding overlaps with the ACEL gene set were one and four genes. Across the total unique MAGE set, seven genes overlapped with PMJ and four genes overlapped with ACEL. *USF2* was the only gene shared by MAGE and both external studies. The gene-level pairwise and three-way overlaps are reported in Supplementary Table SM. 3.

**Table 2:** Overlap of MAGE-selected unique gene sets with the 509 aging-related genes reported by Yang *et al*. [68] in the PMJ and the 130 genes with rank *R≥ 6* reported by Coler-Reilly *et al*. [69] in ACEL. The three-way overlap denotes unique genes shared by the corresponding MAGE set and both external gene sets.

MAGE gene set	Unique genes, *n*	PMJ/Yang *et al*. (*n=509*)	ACEL/Coler-Reilly *et al*. (*n=130*)	Three-way overlap
MAGE MAX	118	5	1	0
MAGE MIN	117	2	4	1
MAGE Total Unique	206	7	4	1

## Conclusion

In this study, we introduced MAGE, a novel algorithm designed to uncover gene subsets whose regulatory influence consistently changes with age across various human tissues. Aging is a complex, multisystem biological process, and current methods often lack sensitivity to time-dependent gene interactions. Our approach addresses this gap by defining a novel metric, called the Age Index, which captures directional influence trends in age-dependent GRNs. The MAGE algorithm, a stochastic optimization method, efficiently identifies gene sets with maximal or minimal Age Index scores, providing insights into ascending or declining transcriptional changes linked to aging.

We emphasize that the “regulatory influence” quantified by the Age Index and MAGE should be interpreted as a topological and statistical association measure within an inferred GRN context, rather than as verified direct biophysical regulation or causal mechanistic control. The inferred influence dynamics quantify how the relative contribution of genes or gene sets varies across age intervals based on shortest-path relationships in the constructed regulatory networks. Therefore, the reported influence trajectories indicate age-dependent network association patterns, not experimentally validated causal gene regulation. We emphasize that the regulatory influence captured by the Age Index and MAGE reflects statistical association patterns inferred from GRNs rather than direct causal regulatory changes. The inferred influence dynamics quantify how the relative contribution of genes or gene sets varies across age intervals within the network context. Consequently, the results should be interpreted as indicative of age-dependent regulatory influence patterns, not as evidence of causal gene regulation or direct mechanistic control.

### Biological scope and confounding factors

A key limitation of the proposed framework is that it operates exclusively on transcriptomic data and inferred GRNs, without explicitly incorporating epigenomic or proteomic layers. Aging is a multilayered biological process involving chromatin accessibility, DNA methylation, histone modifications, and posttranslational regulation, which are not directly captured by transcript-level analysis. As a result, the regulatory influence patterns (representing topological and statistical association pathways within an inferred network) identified by MAGE should be interpreted as transcriptome-driven signals rather than comprehensive representations of molecular aging mechanisms.

In addition, the analysis is performed at the tissue level using bulk expression profiles, which may be confounded by age-related changes in cell-type composition. Shifts in the relative abundance of constituent cell populations across age groups can influence observed expression patterns and inferred regulatory influence dynamics. While MAGE focuses on network-level aggregation and relative influence trends, such compositional effects may contribute to the variability observed in certain tissues. Integrating cell-type–resolved or single-cell data, as well as multi-omics layers, represents an important direction for future extensions of the framework.

As the present study focuses on tissue-level regulatory influence dynamics, explicit cell-type deconvolution and cell-type marker enrichment analyses were beyond its primary scope; therefore, we do not interpret the highest- or lowest-scoring tissue-specific gene sets as direct evidence of cell-type abundance changes. However, age-associated shifts in cellular composition may partly contribute to the observed tissue-specific Age Index patterns, particularly in highly heterogeneous tissues such as whole blood, immune-related tissues, and complex brain regions. Accordingly, the highest and lowest MAGE-scoring tissue-specific gene sets should be interpreted as network-level aging-associated signals that may include both regulatory changes and cell-composition effects. Future work integrating single-cell or spatial transcriptomic profiles will allow us to evaluate whether MAGE-selected gene sets overlap with known cell-type marker fluctuations and to distinguish regulatory aging signals from compositional confounding.

We demonstrated the robustness and accuracy of MAGE through comprehensive statistical and biological validations. Statistical evaluation using Z-score analyzes confirmed that the selected gene clusters are not random but biologically relevant, and showed significant overexpression or repression patterns with aging. Biological validation further reinforced the relevance of the method, identifying known aging-associated genes and their connections to physiological systems such as the nervous, endocrine, and immune systems. Our algorithm proved consistent across multiple tissues, highlighting both shared and tissue-specific aging signatures.

Clinically, the identified genes are linked to systems commonly affected by aging, such as the nervous, endocrine, and immune systems, and may serve as potential biomarkers. Its innovation lies in the Age Index metric, which models time-dependent changes in gene influence, setting MAGE apart from conventional time-independent methods. Compared with existing approaches, MAGE incorporates temporal dynamics and regulatory network structure to capture consistent transcriptional trends with age. In this manuscript, we also introduce the MAP for the first time; to the best of our knowledge, no previous study has addressed this problem. Limitations include reliance on specific datasets, sensitivity to parameter choices, and the absence of other biological layers such as epigenetics.

MAGE offers a powerful, scalable solution for detecting regulatory gene sets associated with aging. It lays the groundwork for future studies into age-related diseases and provides a basis for identifying potential biomarkers and therapeutic targets, advancing our understanding of the molecular mechanisms underpinning human aging.

**Key Points**

- Aging is characterized by progressive, tissue-specific transcriptional changes driven by genomic instability and regulatory network rewiring.
- Gene expression dynamics with age are heterogeneous across tissues, underscoring the need for integrative, age-aware modeling frameworks.
- Gene regulatory networks provide a systems-level representation of how transcriptional influence among genes evolves during aging.
- Contributions are two-fold: (i) Age Index, a network-based metric that quantifies time-varying gene influence; (ii) an algorithm, called MAGE (Maximal Age Index Algorithm), to compute age indexes and to identify statistically significant gene sets with consistent age-dependent patterns.

## Supplementary Material

MAGE_Age_Index_BiB_SM_bbag613

## Data Availability

MAGE code and datasets are available at https://github.com/OmerKahveci03/Gene-Normalization-and-Mapping. A versioned code release has been archived on Zenodo and is available at DOI: https://doi.org/10.5281/zenodo.20673366.
