# Supplementary material for: MAGE: an efficient algorithm for identifying Maximal Age-indexed GEne sets
Source: Brief Bioinform. 2026 Nov 2;27(6):bbag613. doi: 10.1093/bib/bbag613 (PMC13725925; doi:10.1093/bib/bbag613)
Supplement: Supplementary file 1 [file mage_age_index_bib_sm_bbag613.pdf]

Supplementary materials

NP hardness of MAP+

We prove the NP-hardness of the MAP+ problem by reducing from the **Maximum Acyclic Subgraph (MAS)** problem, which is known to be NP-hard. We start by defining the MAS problem.
Maximum Acyclic Subgraph (MAS) problem
INPUT: A directed graph $H = (W, F)$.
GOAL: Find an ordering of the vertices $w_1, w_2, \ldots, w_t$ (a permutation of W) that maximizes the number of edges oriented forward in the ordering, i.e., edges $(w_i, w_j) \in F$ such that $i < j$.
Reduction: Given an instance $H = (W, F)$ of MAS, where $|W| = t$, construct an instance of our problem as follows:

- Define the common vertex set $V = \{x, y_1, y_2, \ldots, y_t\}$.
- For each $1 \leq i \leq t$, construct a graph G_i with edges:

$$E_i = \{(x, y_j) \mid (w_i, w_j) \in F\}$$

  That is, from x, we add edges to y_j in G_i if w_i has an edge to w_j in the MAS instance.
- Fix $V' = \{x\}$. Then, for each i, reach(V', G_i) consists of:

$$\text{reach}(\{x\}, G_i) = \{x\} \cup \{y_j \mid (w_i, w_j) \in F\}$$

  i.e., the out-neighbors of w_i in H.

Why this reduction works. The function $\psi(G_i, G_j, V')$ of MAP+ compares the reachability sets of G_i and G_j:

- $\psi(V', G_i, G_j) = 1$ if the out-neighbors of w_i are a subset of those of w_j
- $\psi(V', G_i, G_j) = -1$ if the reverse holds
- $\psi(V', G_i, G_j) = 0$ otherwise

Maximizing the total score of $f(G_i, G_j, V')$ over all $1 \leq i < j \leq t$ encourages a vertex ordering where the out-neighborhoods grow monotonically, which also maximizing the number of forward edges in the MAS ordering.
In summary. If we could solve this instance of MAP+ in polynomial time, we could solve MAS, which is NP-hard. Therefore, our problem is NP-hard as well.

□

Theoretical properties of the MAP$^+$

In this section, we analyze structural properties of the Maximal Age Index Problem. Without losing generality, we focus on the MAP$^+$ variant of the problem. All the findings of the section also applies to the MAP$^-$ variant as well.

Let $\mathcal{G} = [G_{a_1}, G_{a_2}, \ldots, G_{a_t}]$ be a fixed sequence of age-specific GRNs, and define

$$f(S) := AI(S, \mathcal{G})$$

for any subset of genes $S \subseteq V$. We prove five properties of the MAP$^+$ objective, namely (i) non-monotonicity, (ii) non-submodularity, (iii) lack of Lipschitz continuity, (iv) unbounded marginal effects, and (v) absence of cardinality constraints.

Proposition 1 (NON-MONOTONICITY) *The MAP$^+$ objective function $f(S)$ is not monotone.*

Proof A set function f is monotone if $f(S) \leq f(T)$ for all $S \subseteq T$. Consider a GRN sequence consisting of two ages, $\mathcal{G} = [G_{a_1}, G_{a_2}]$, and let $\epsilon > 0$ be the minimum observable change.

Let $u, v \in V$ be two genes such that

$$I(\{u\}, G_{a_1}) > I(\{u\}, G_{a_2}) + \epsilon, \quad I(\{v\}, G_{a_1}) < I(\{v\}, G_{a_2}) - \epsilon.$$

Then $f(\{u\}) = 1$ and $f(\{v\}) = -1$.

Let $S = \{u\}$ and $T = \{u, v\}$. Since influence is defined via a maximum over set elements,

$$I(T, G_{a_i}) = \max\{I(\{u\}, G_{a_i}), I(\{v\}, G_{a_i})\}, \quad i = 1, 2.$$

It is possible that

$$|I(T, G_{a_1}) - I(T, G_{a_2})| \leq \epsilon,$$

in which case $f(T) = 0 < f(S)$. Hence f is not monotone. □

Proposition 2 (NON-SUBMODULARITY) *The MAP$^+$ objective function $f(S)$ is not submodular.*

Proof Submodularity requires diminishing returns: for all $S \subseteq T \subseteq V$ and $u \notin T$,

$$f(S \cup \{u\}) - f(S) \geq f(T \cup \{u\}) - f(T).$$

Consider $\mathcal{G} = [G_{a_1}, G_{a_2}]$ and genes $u, v, w \in V$. Suppose that adding w to $\{u\}$ does not change the Age Index, but adding w to $\{u, v\}$ causes the influence difference to exceed the threshold ϵ, yielding

$$f(\{u\}) = f(\{u, w\}) = 0, \quad f(\{u, v\}) = 0, \quad f(\{u, v, w\}) = 1.$$

Then

$$f(\{u, w\}) - f(\{u\}) = 0 < 1 = f(\{u, v, w\}) - f(\{u, v\}),$$

violating the diminishing returns property. Therefore, f is not submodular. □

Proposition 3 (Lack of smoothness or continuity) *The MAP$^+$ objective function is neither smooth nor continuous.*

Proof The Age Index is computed using the indicator function

$$\psi(S, G_{a_i}, G_{a_j}) = \begin{cases} 1 & \text{if } I(S, G_{a_i}) > I(S, G_{a_j}) + \epsilon, \\ -1 & \text{if } I(S, G_{a_i}) < I(S, G_{a_j}) - \epsilon, \\ 0 & \text{otherwise.} \end{cases}$$

This definition introduces jump discontinuities at thresholds $\pm\epsilon$. An arbitrarily small change in influence values can cause ψ to change abruptly from 0 to ± 1, leading to a discontinuous change in $f(S)$. Thus, f is neither continuous nor smooth. □

Proposition 4 (Lack of Lipschitz continuity) *The MAP$^+$ objective function admits bounded absolute differences but is not Lipschitz-continuous with respect to changes in influence values.*

Proof By definition, the age index satisfies $f(S) \in [-1, 1]$ for all $S \subseteq V$. Consequently, for any $u \notin S$,

$$|f(S \cup \{u\}) - f(S)| \leq 2.$$

Thus, the objective admits a global absolute bound on marginal differences.

However, the age index is computed using thresholded indicator functions. An arbitrarily small perturbation in influence values (such as adding a gene whose influence changes $I(S, G_{a_i}) - I(S, G_{a_j})$ by an arbitrarily small amount) can cause this difference to cross the threshold ϵ, resulting in a discontinuous change in $\psi(S, G_{a_i}, G_{a_j})$ from 0 to ± 1. Such a change may propagate across multiple age pairs, leading to a jump of $f(S)$ by a constant amount independent of the magnitude of the perturbation.

Therefore, while absolute differences are bounded, no Lipschitz constant exists that relates changes in influence values to changes in the objective function. This lack of continuity precludes smooth approximation guarantees. □

Proposition 5 (Absence of cardinality constraint) *The MAP$^+$ problem does not impose a cardinality constraint on the solution set.*

Proof By definition, MAP$^+$ seeks to maximize $f(S)$ over all subsets $S \subseteq V$:

$$\max_{S \subseteq V} f(S).$$

No restriction is placed on $|S|$. The optimal solution may consist of a single gene, a small subset, or the entire vertex set. Any cardinality constraint (e.g., selecting exactly k genes) is therefore algorithmic rather than intrinsic to the problem formulation. □

Implications of the five properties above. The absence of monotonicity, submodularity, smoothness, Lipschitz continuity, and inherent cardinality constraints implies that MAP$^+$ does not fall into standard classes of combinatorial optimization problems for which approximation guarantees are available. Thus, heuristic and stochastic methods such as MAGE are appropriate for exploring high-quality solutions in practice.

List of tissues

Table SM. 1 lists the 48 tissues studied in our experiments and the anatomical groups they belong to.

Table SM. 1. Anatomical classification of human tissues used in the MAGE analysis.

Anatomical Group	Tissues
Nervous System	brain amygdala<br>brain anterior cingulate cortex ba24<br>brain caudate basal ganglia<br>brain cerebellar hemisphere<br>brain cerebellum<br>brain cortex<br>brain frontal cortex ba9<br>brain hippocampus<br>brain hypothalamus<br>brain nucleus accumbens basal ganglia<br>brain putamen basal ganglia<br>brain spinal cord cervical c-1<br>brain substantia nigra<br>nerve tibial
Cardiovascular System	artery aorta<br>artery coronary<br>heart atrial appendage<br>heart left ventricle<br>whole blood
Digestive System	colon transverse<br>esophagus mucosa<br>esophagus muscularis<br>liver<br>small intestine terminal ileum<br>stomach<br>minor salivary gland
Respiratory System	lung
Endocrine System	adrenal gland<br>thyroid<br>pituitary
Immune System	spleen<br>cells ebv-transformed lymphocytes
Integumentary System	skin sun exposed lower leg<br>skin not sun exposed suprapubic<br>muscle skeletal<br>cells cultured fibroblasts
Glandular/Mixed Tissue	breast mammary tissue<br>minor salivary gland
Reproductive System	ovary<br>prostate<br>testis<br>uterus<br>vagina
Adipose Tissue	adipose subcutaneous<br>adipose visceral omentum

Table SM. 2. Top enriched GO:BP terms among MAGE-selected genes.

Term ID	Term Name	Related Genes
GO:0006357	regulation of transcription by RNA polymerase II	AHR, ARNT, ATF6, BMI1, BRIP1, CBX7, CEBPD, CEBPZ, CIITA, DLX5, E2F8, EOMES, ETS2, EZH2, GATA1, GATA4, GATA6, GLI1, HIPK2, HMGA1, ID3, JUND, MYB, NANOG, NOTCH3, NR1I2, PAX3, SRF, PAX5, TP63
GO:0070887	cellular response to chemical stimulus	AHR, APC, ARNT, BRIP1, CIITA, EZH2, GATA1, GATA4, GATA6, HIPK2, ID3, JUND, MYB, NR1I2, SRF, TP63
GO:1903131	mononuclear cell differentiation	BMI1, EOMES, EZH2, GATA1, MYB, SRF, PAX5

Table SM. 3. Gene-level overlaps between the unique MAGE-selected gene sets and the aging-related gene sets reported by Yang et al. in the *Postgraduate Medical Journal* (PMJ; $n = 509$) and by Coler-Reilly et al. in *Aging Cell* (ACEL; $n = 130$, $R \geq 6$). Gene symbols selected across tissues were pooled and deduplicated before calculating the intersections.

MAGE set	External comparison	Overlap, n	Shared gene symbols
MAGE MAX	PMJ/Yang et al.	5	*JUNB, MCM5, MKL1, NFKB2, NFYC*
MAGE MIN	PMJ/Yang et al.	2	*CEBPA, USF2*
MAGE Total Unique	PMJ/Yang et al.	7	*CEBPA, JUNB, MCM5, MKL1, NFKB2, NFYC, USF2*
MAGE MAX	ACEL/Coler-Reilly et al.	1	*ID2*
MAGE MIN	ACEL/Coler-Reilly et al.	4	*DNMT3A, ID2, RBL2, USF2*
MAGE Total Unique	ACEL/Coler-Reilly et al.	4	*DNMT3A, ID2, RBL2, USF2*
MAGE MAX	PMJ ∩ ACEL	0	–
MAGE MIN	PMJ ∩ ACEL	1	*USF2*
MAGE Total Unique	PMJ ∩ ACEL	1	*USF2*

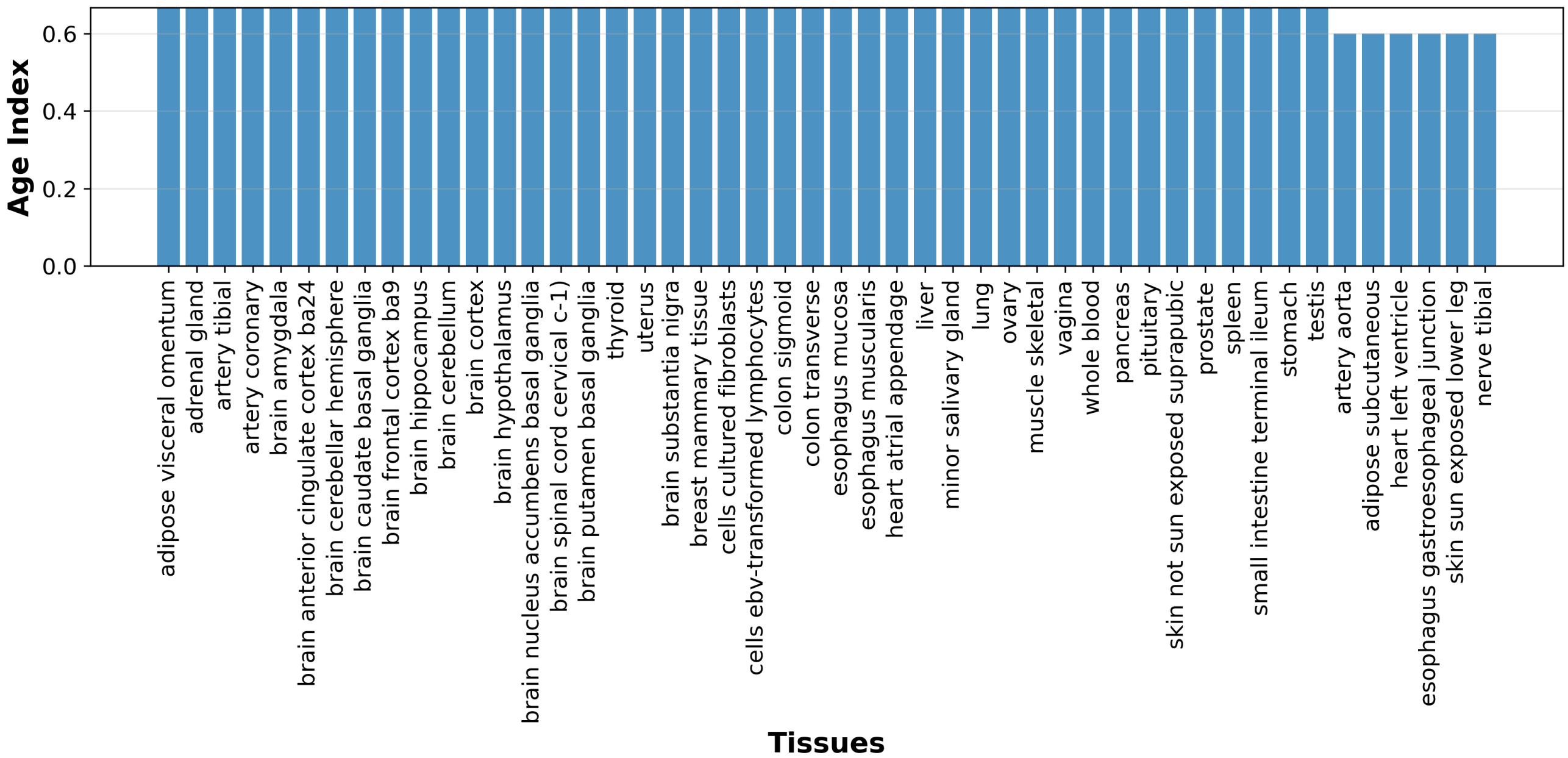

Fig. SM. 1. Maximum Age Index per Tissue for $\lambda = 0.5$, $\epsilon = 0.003$, and $\beta = 0$.

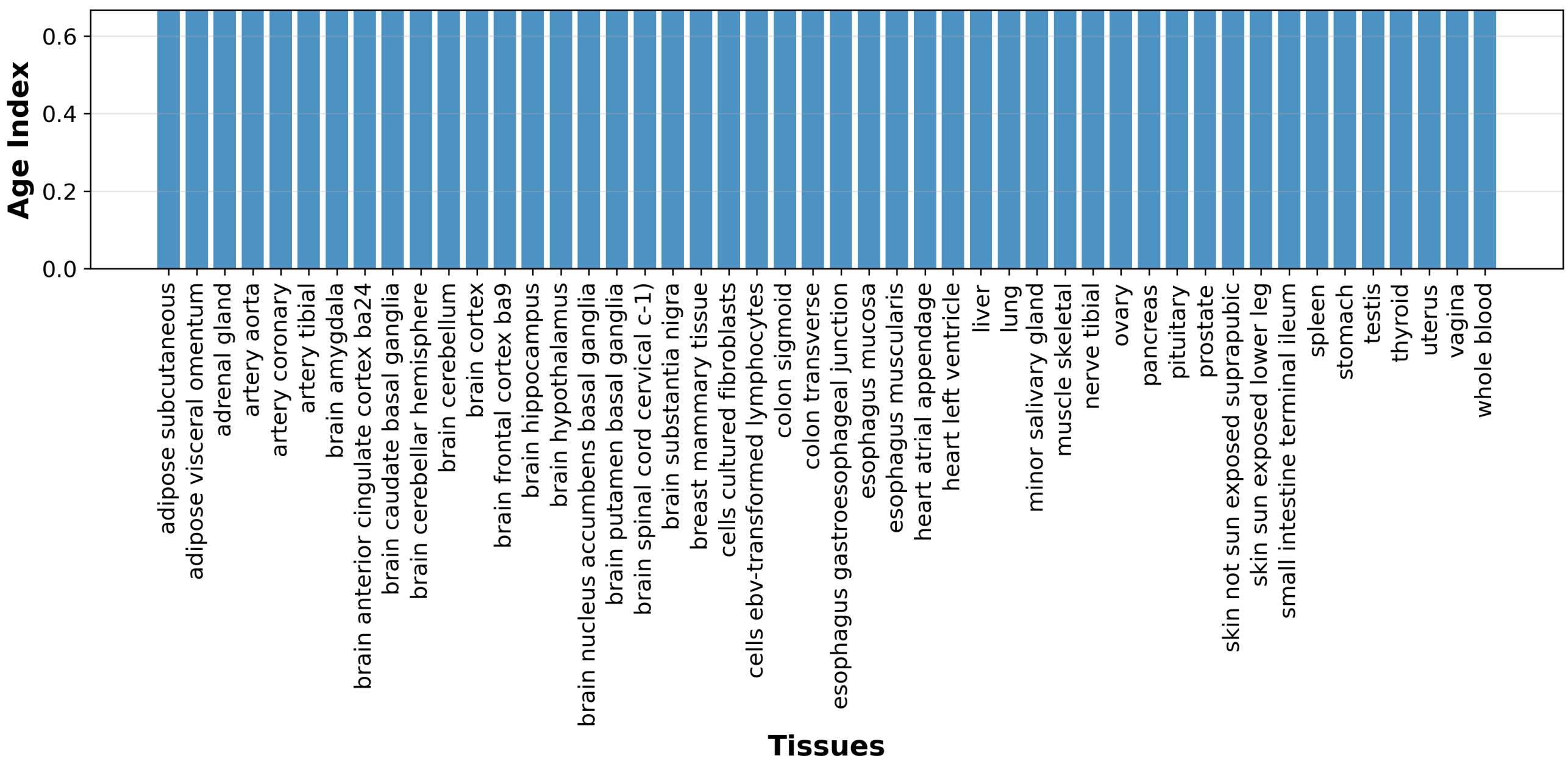

Fig. SM. 2. Maximum Age Index per Tissue for $\lambda = 0.5$, $\epsilon = 0.003$, and $\beta > 0$.

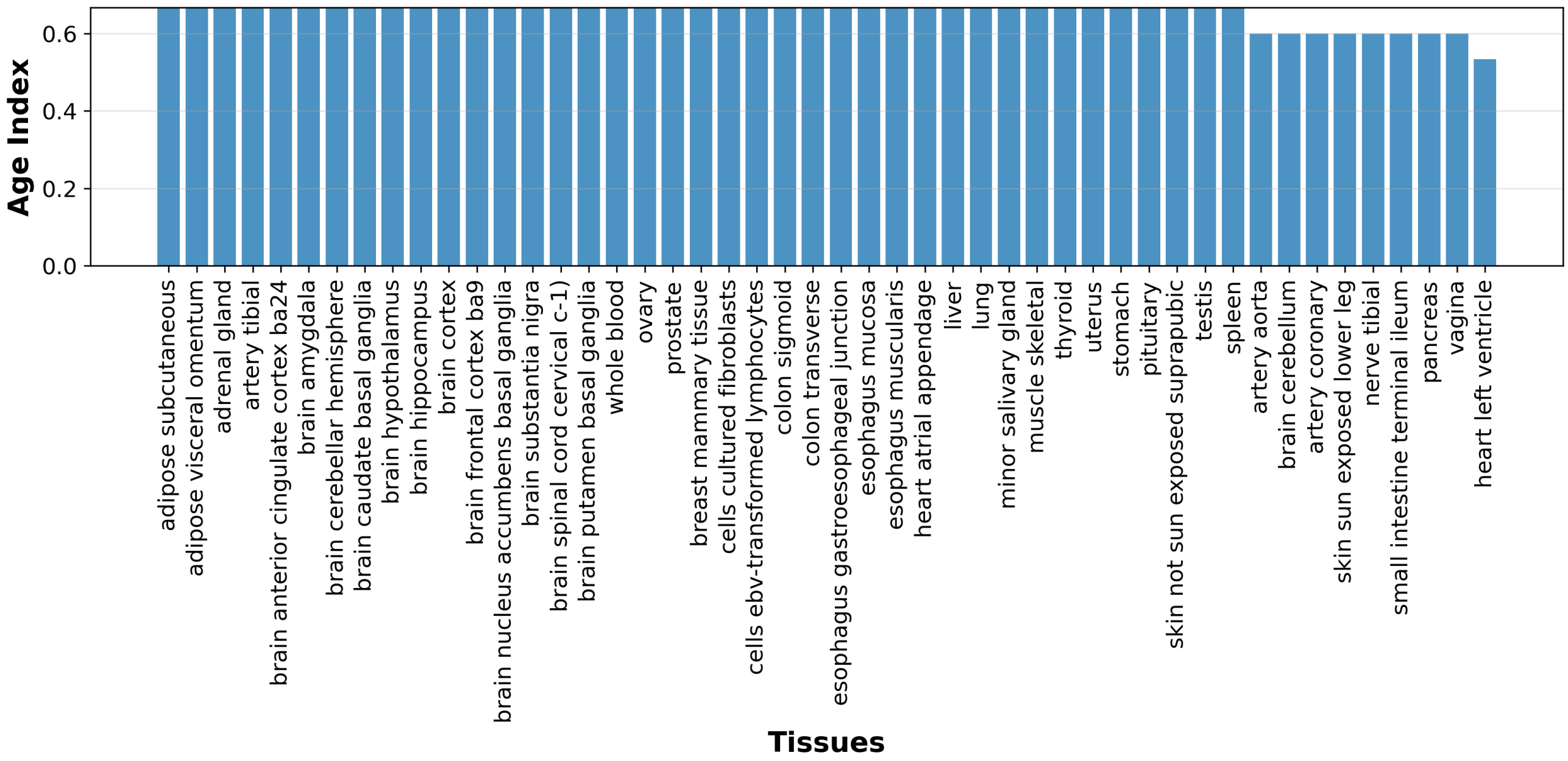

Fig. SM. 3. Maximum Age Index per Tissue for $\lambda = 0.5$, $\epsilon = 0.005$, and $\beta = 0$.

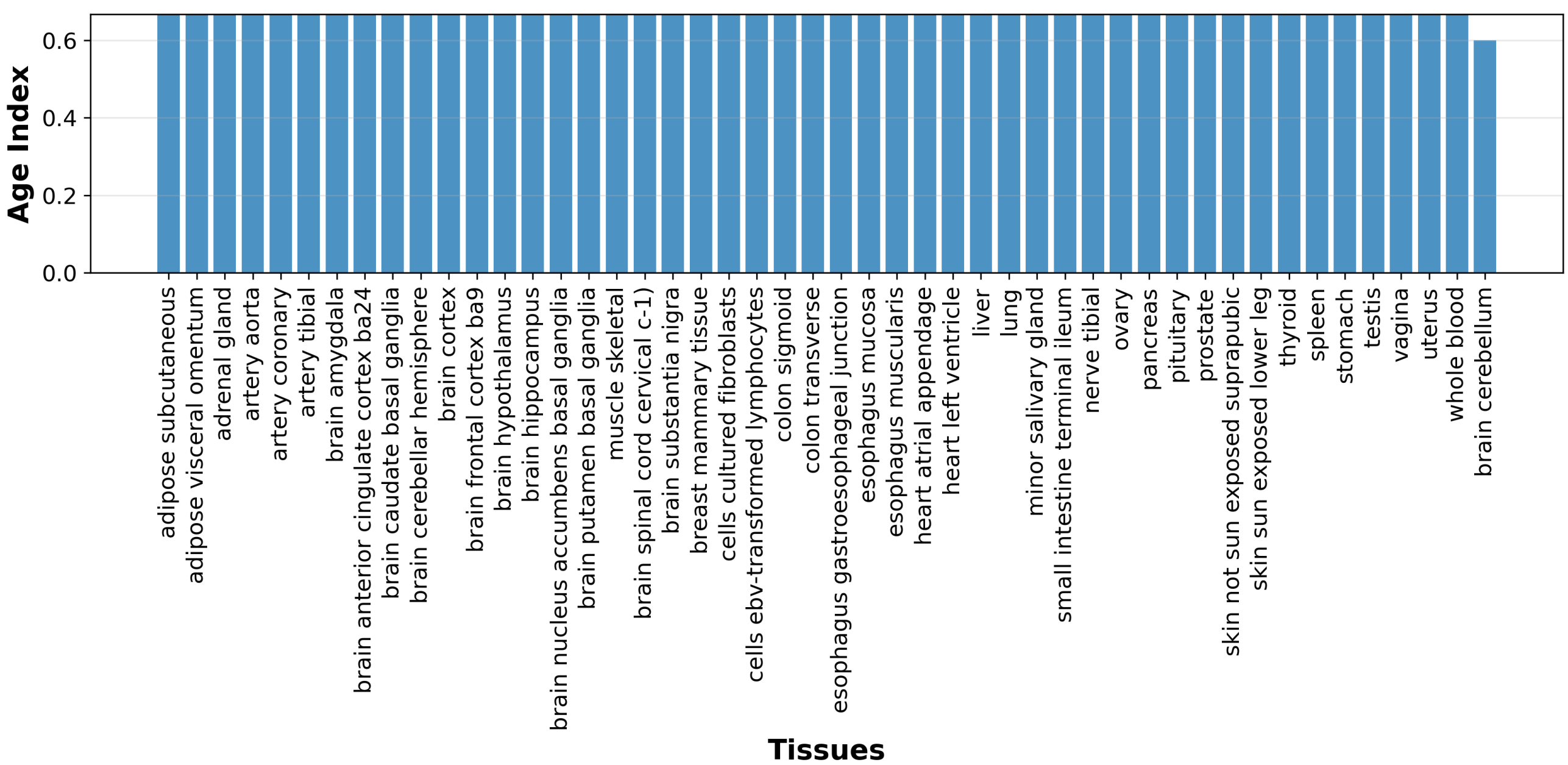

Fig. SM. 4. Maximum Age Index per Tissue for $\lambda = 0.5$, $\epsilon = 0.005$, and $\beta > 0$.

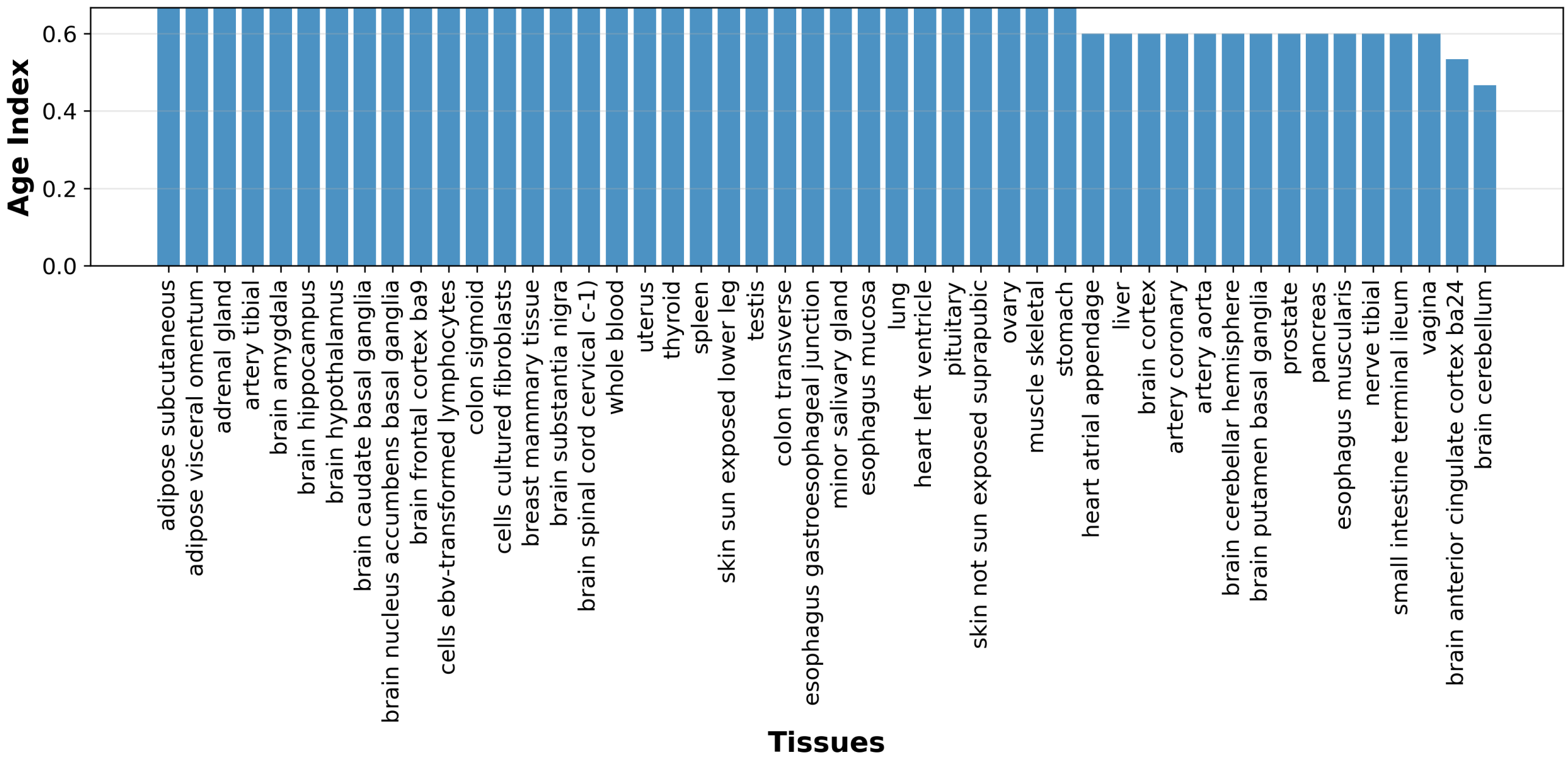

Fig. SM. 5. Maximum Age Index per Tissue for $\lambda = 0.5$, $\epsilon = 0.008$, and $\beta = 0$.

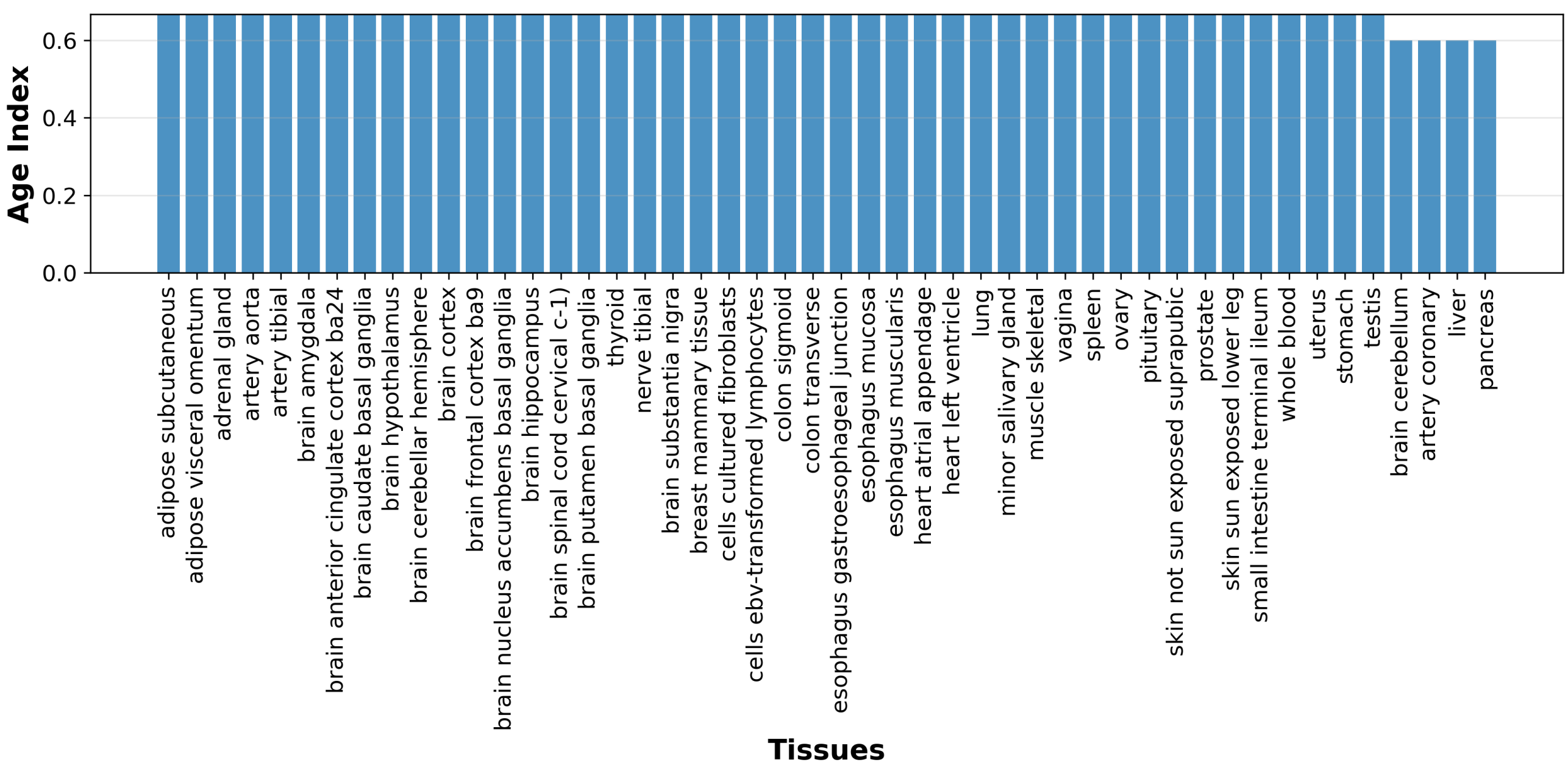

Fig. SM. 6. Maximum Age Index per Tissue for $\lambda = 0.5$, $\epsilon = 0.008$, and $\beta > 0$.

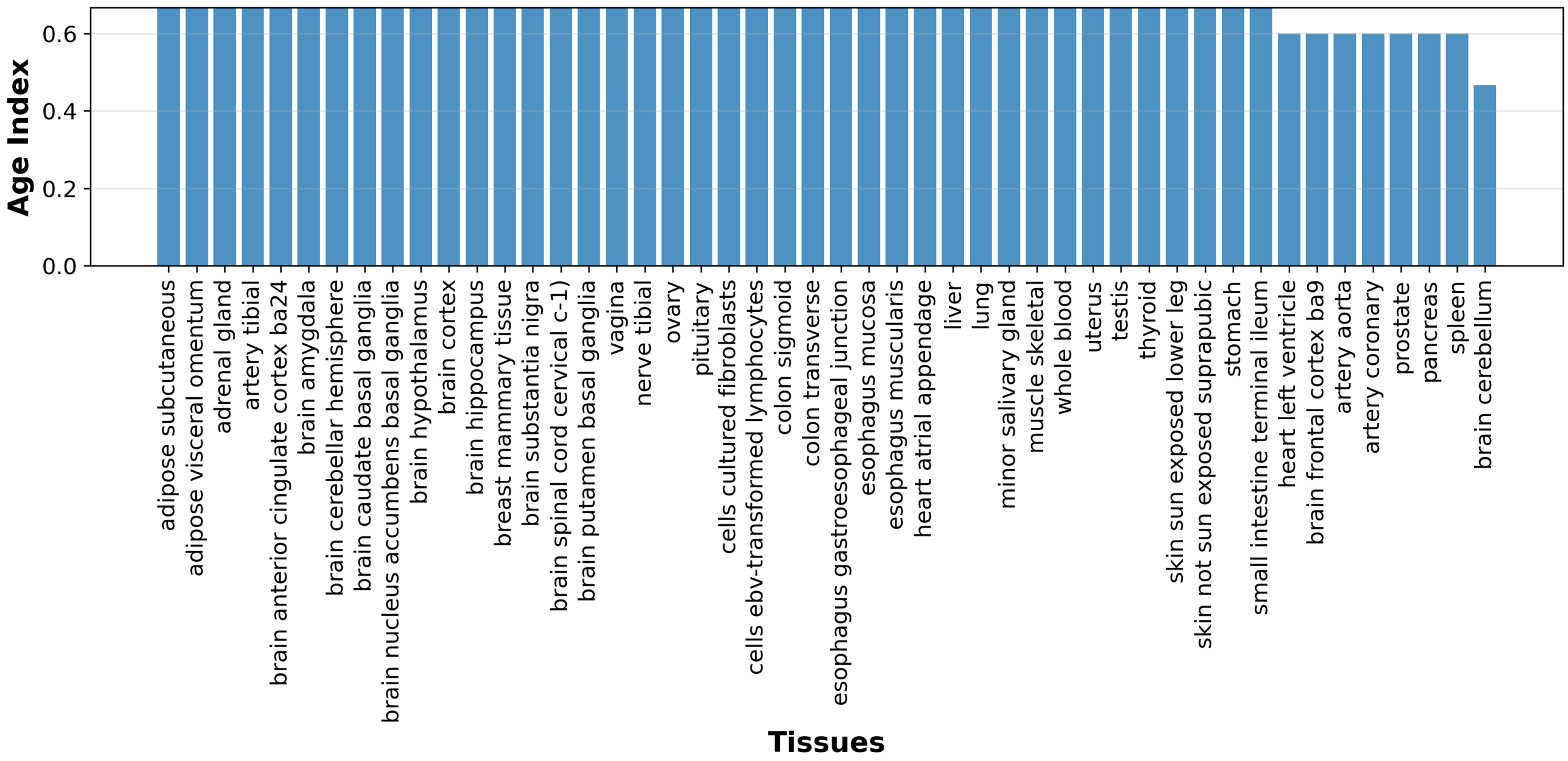

Fig. SM. 7. Maximum Age Index per Tissue for $\lambda = 1.0$, $\epsilon = 0.003$, and $\beta = 0$.

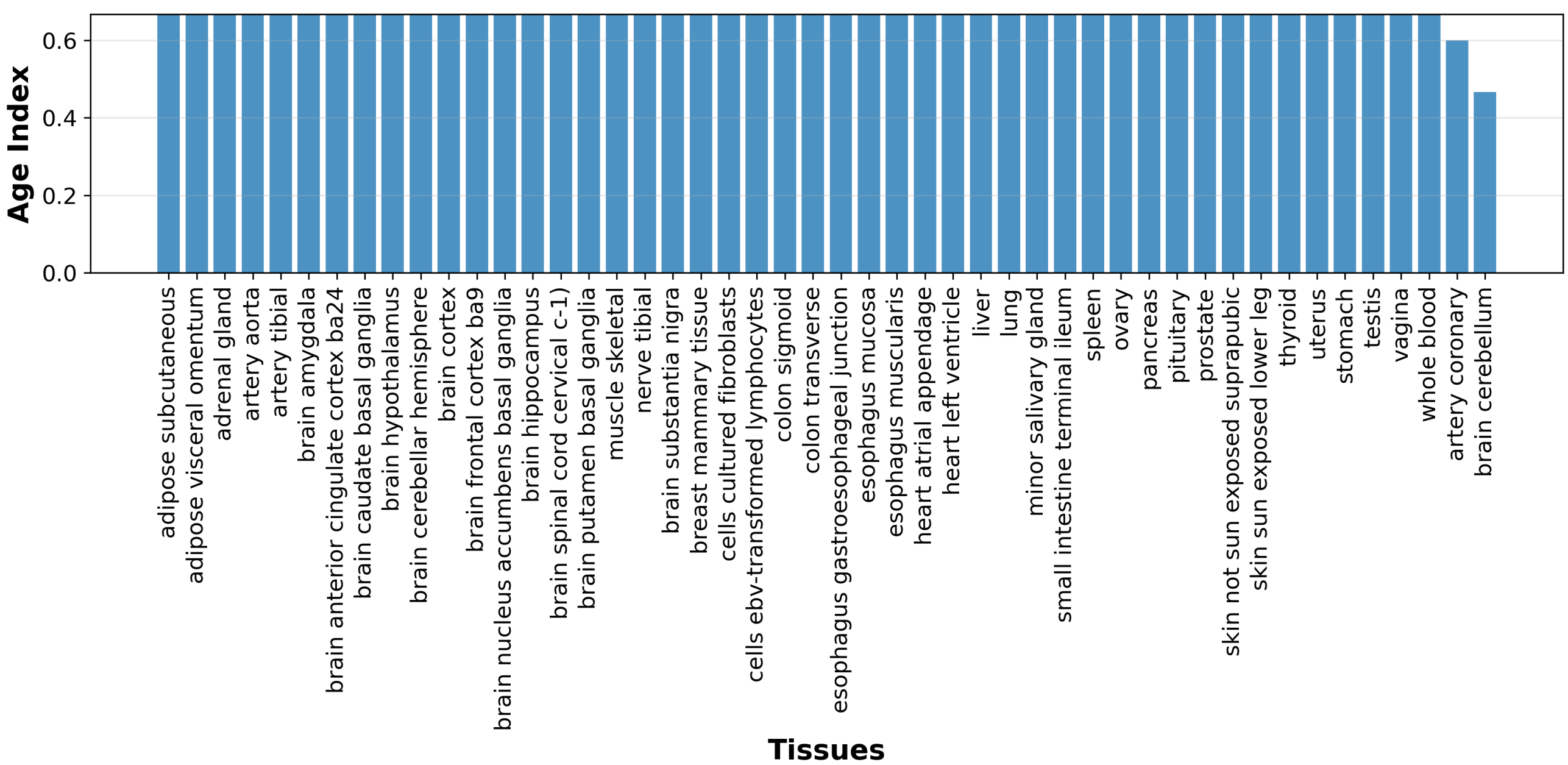

Fig. SM. 8. Maximum Age Index per Tissue for $\lambda = 1.0$, $\epsilon = 0.003$, and $\beta > 0$.

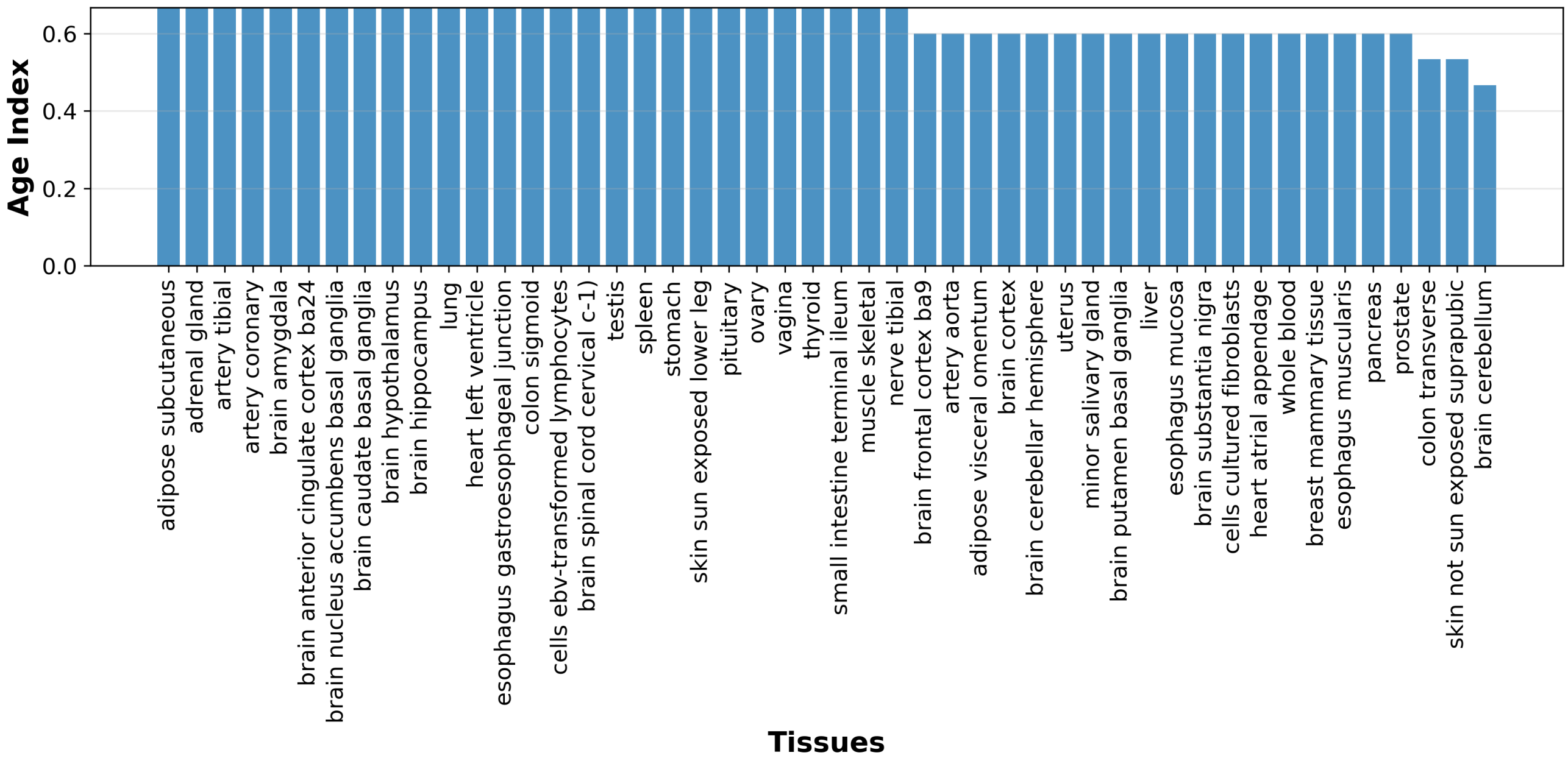

Fig. SM. 9. Maximum Age Index per Tissue for $\lambda = 1.0$, $\epsilon = 0.005$, and $\beta = 0$.

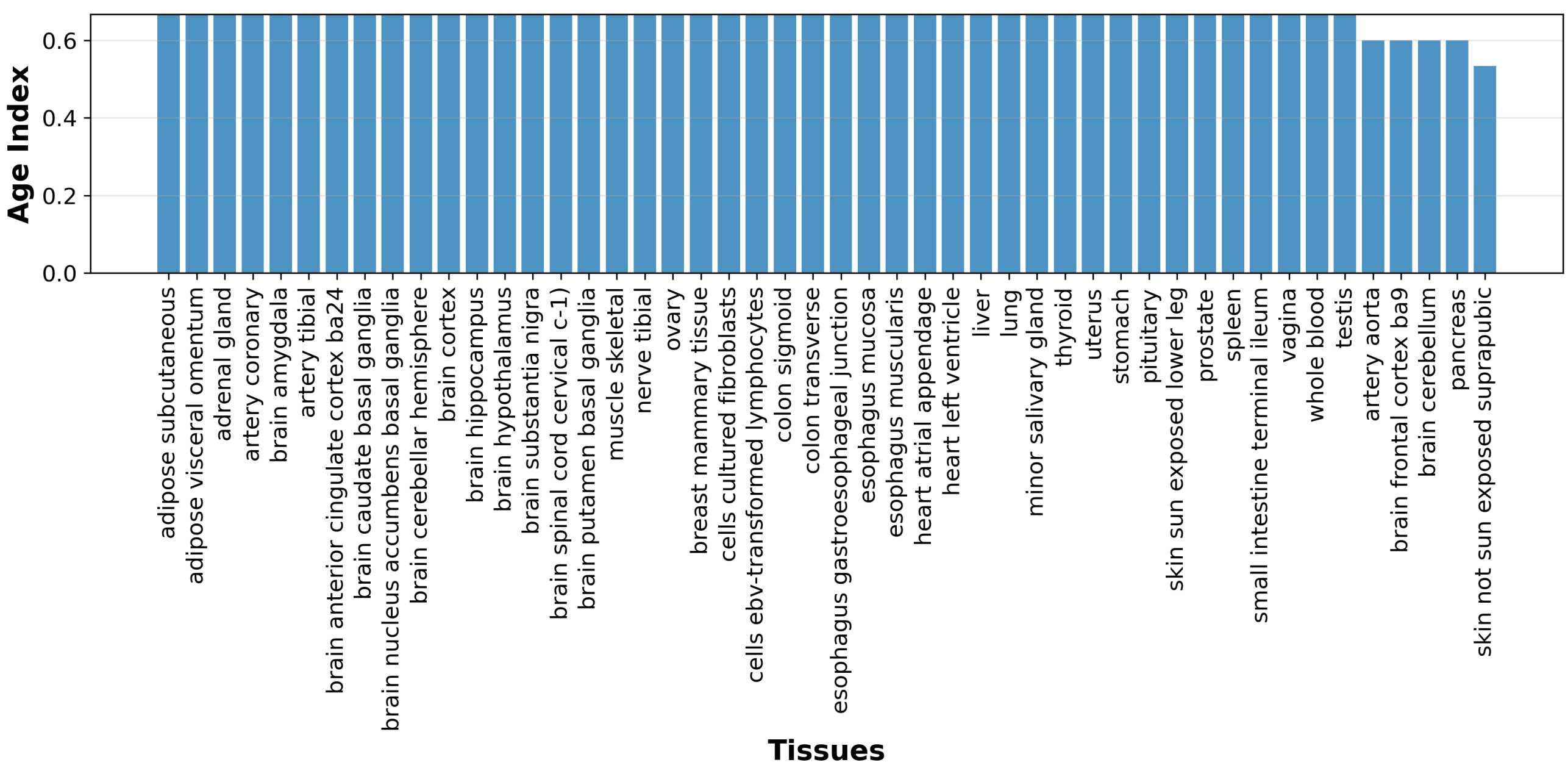

Fig. SM. 10. Maximum Age Index per Tissue for $\lambda = 1.0$, $\epsilon = 0.005$, and $\beta > 0$.

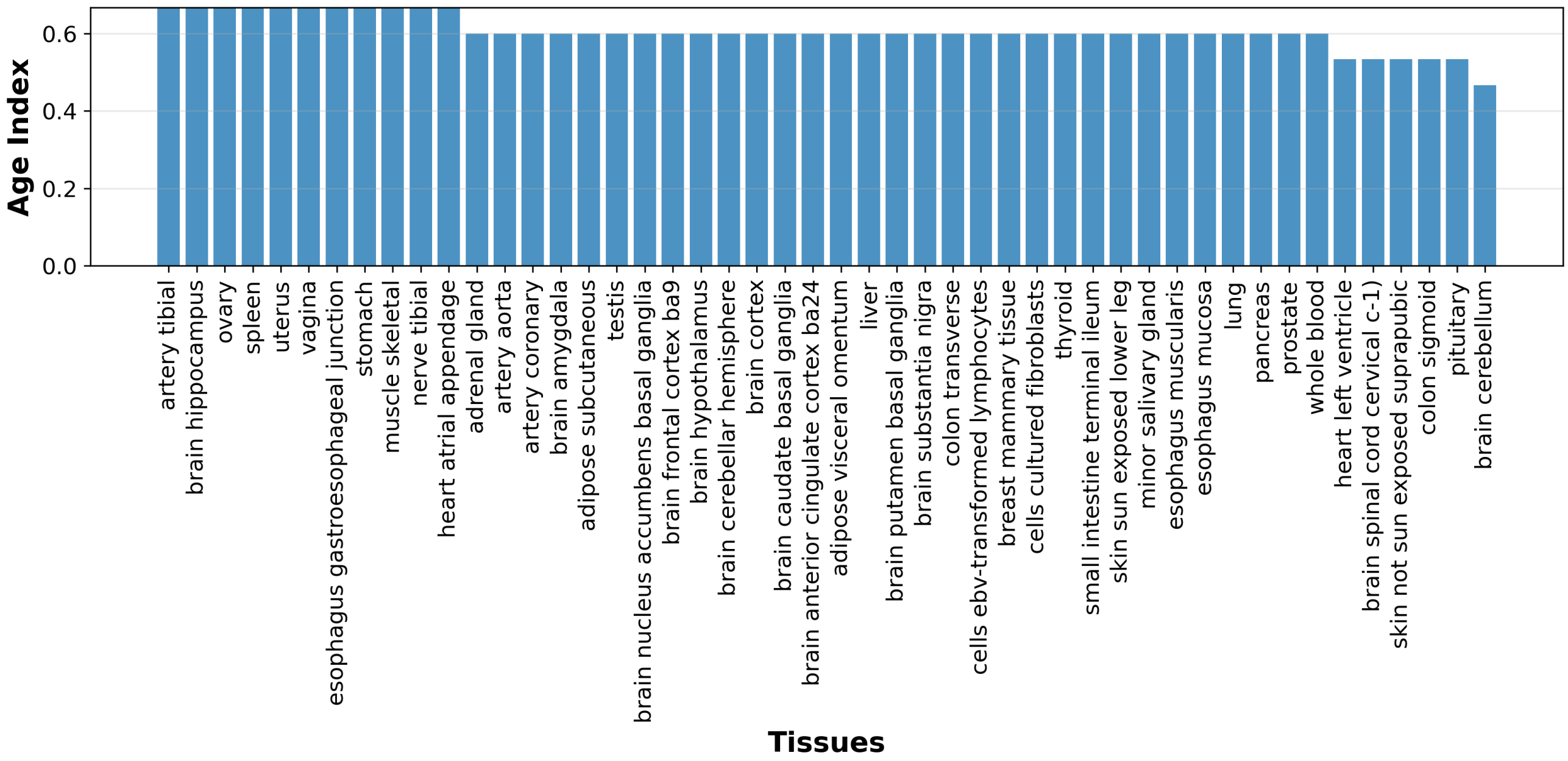

Fig. SM. 11. Maximum Age Index per Tissue for $\lambda = 1.0$, $\epsilon = 0.008$, and $\beta = 0$.

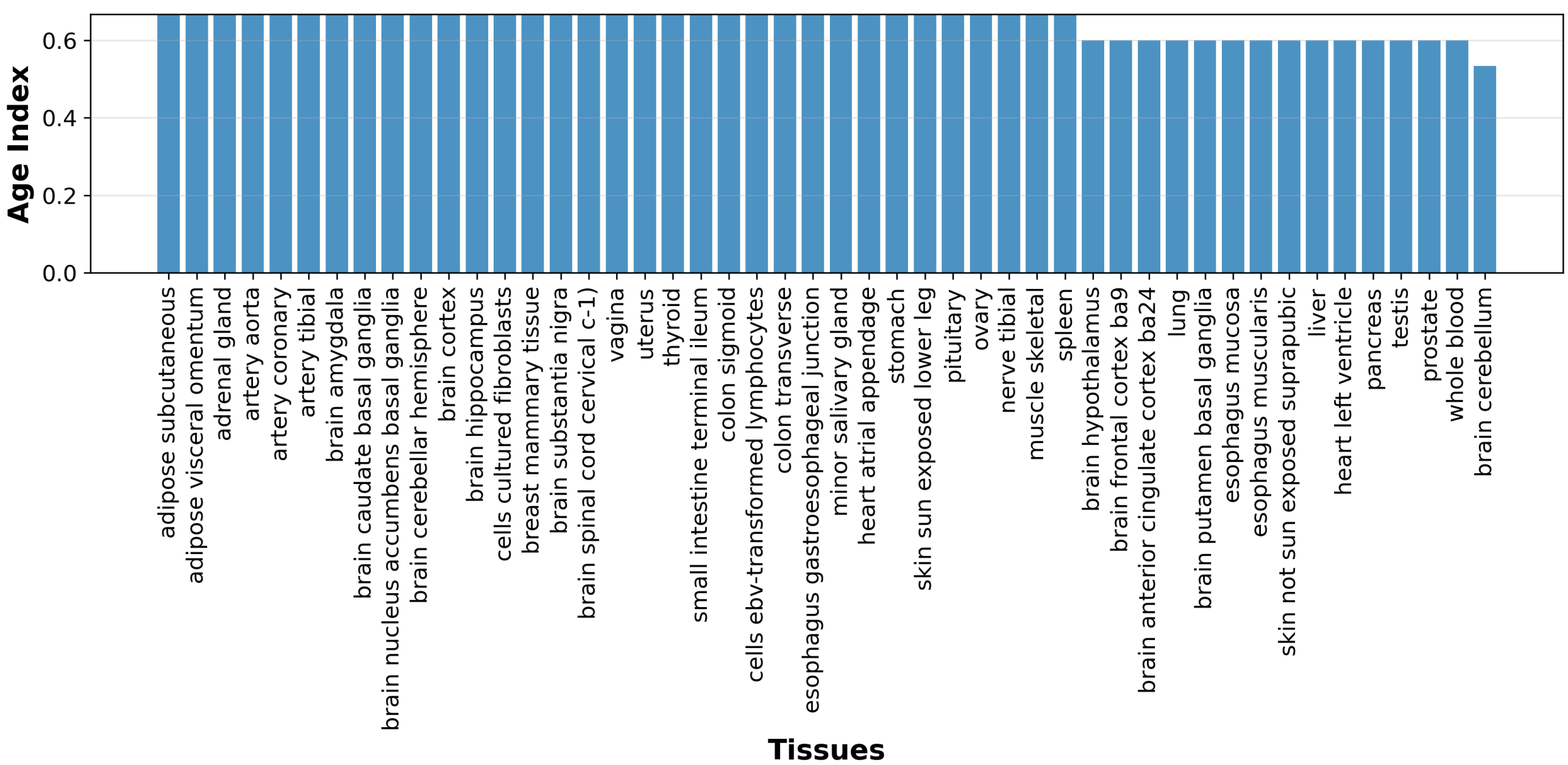

Fig. SM. 12. Maximum Age Index per Tissue for $\lambda = 1.0$, $\epsilon = 0.008$, and $\beta > 0$.

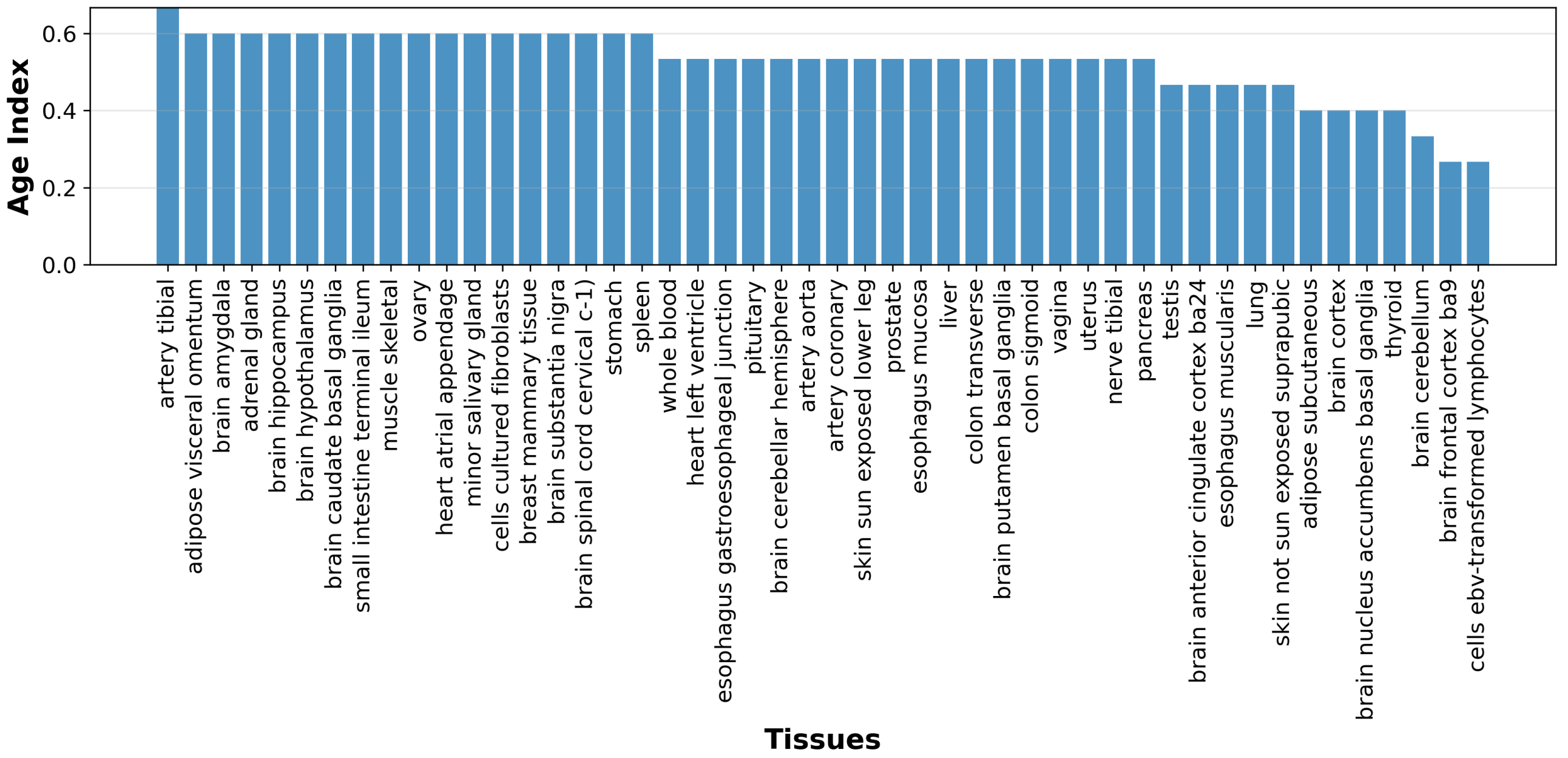

Fig. SM. 13. Maximum Age Index per Tissue for $\lambda = 2.0$, $\epsilon = 0.003$, and $\beta = 0$.

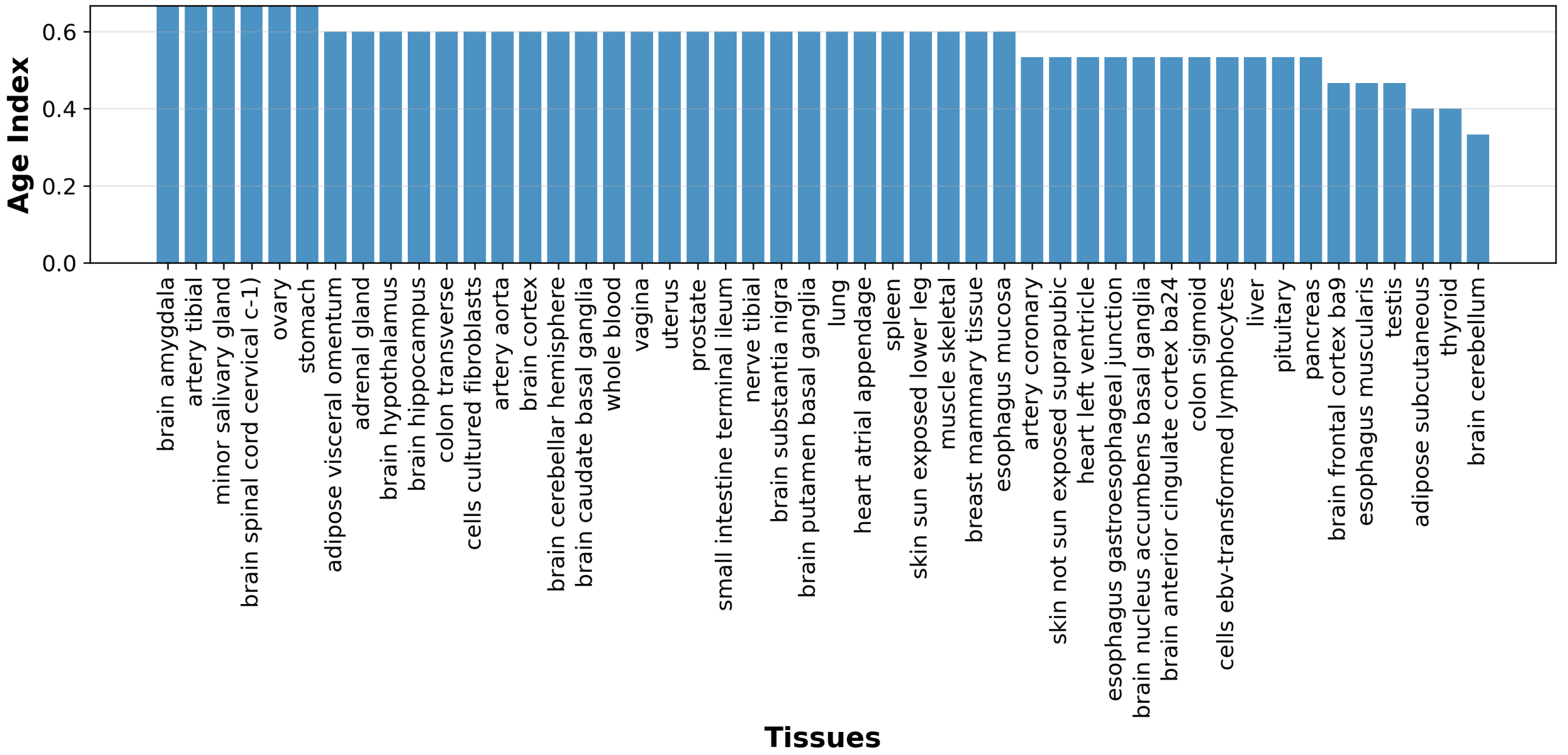

Fig. SM. 14. Maximum Age Index per Tissue for $\lambda = 2.0$, $\epsilon = 0.003$, and $\beta > 0$.

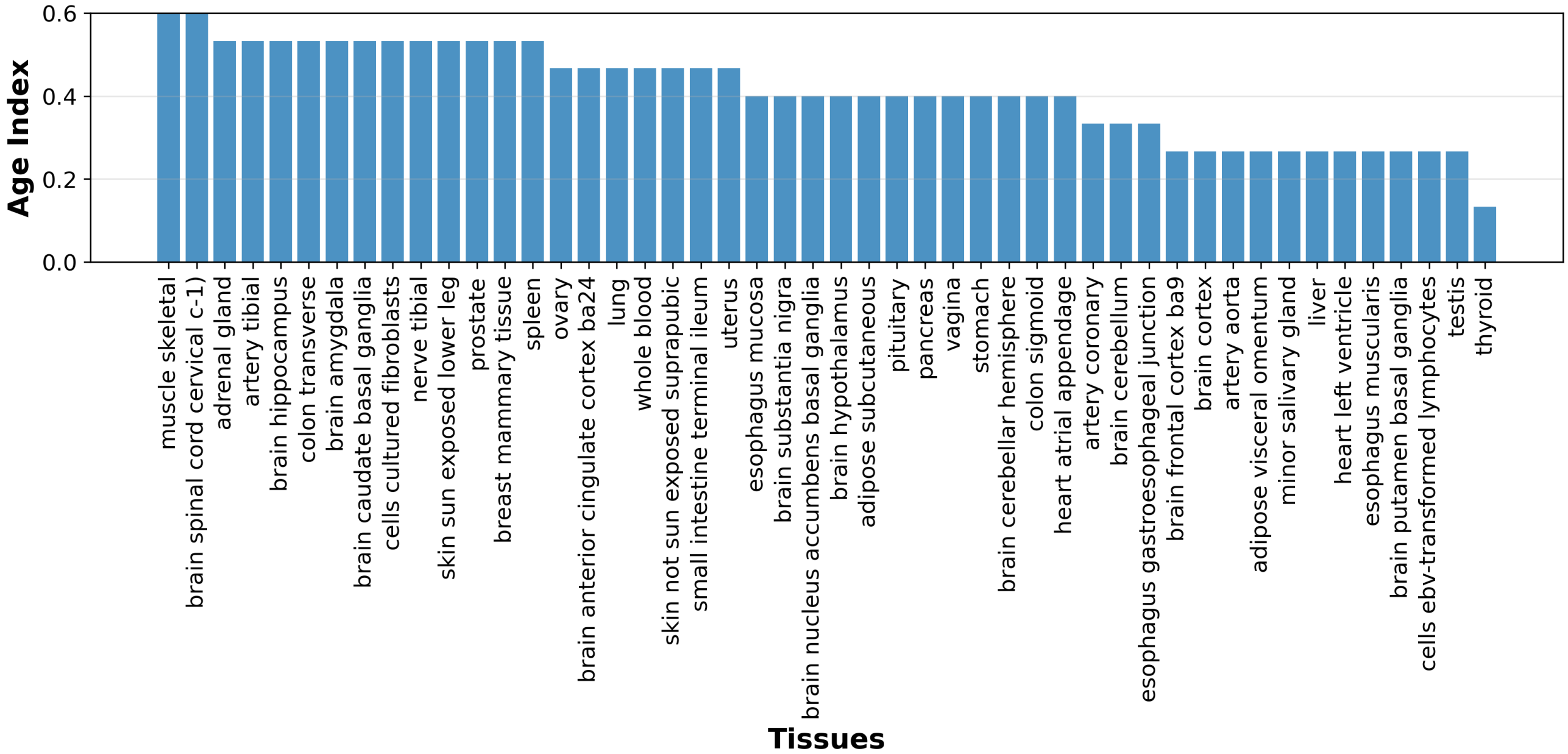

Fig. SM. 15. Maximum Age Index per Tissue for $\lambda = 2.0$, $\epsilon = 0.005$, and $\beta = 0$.

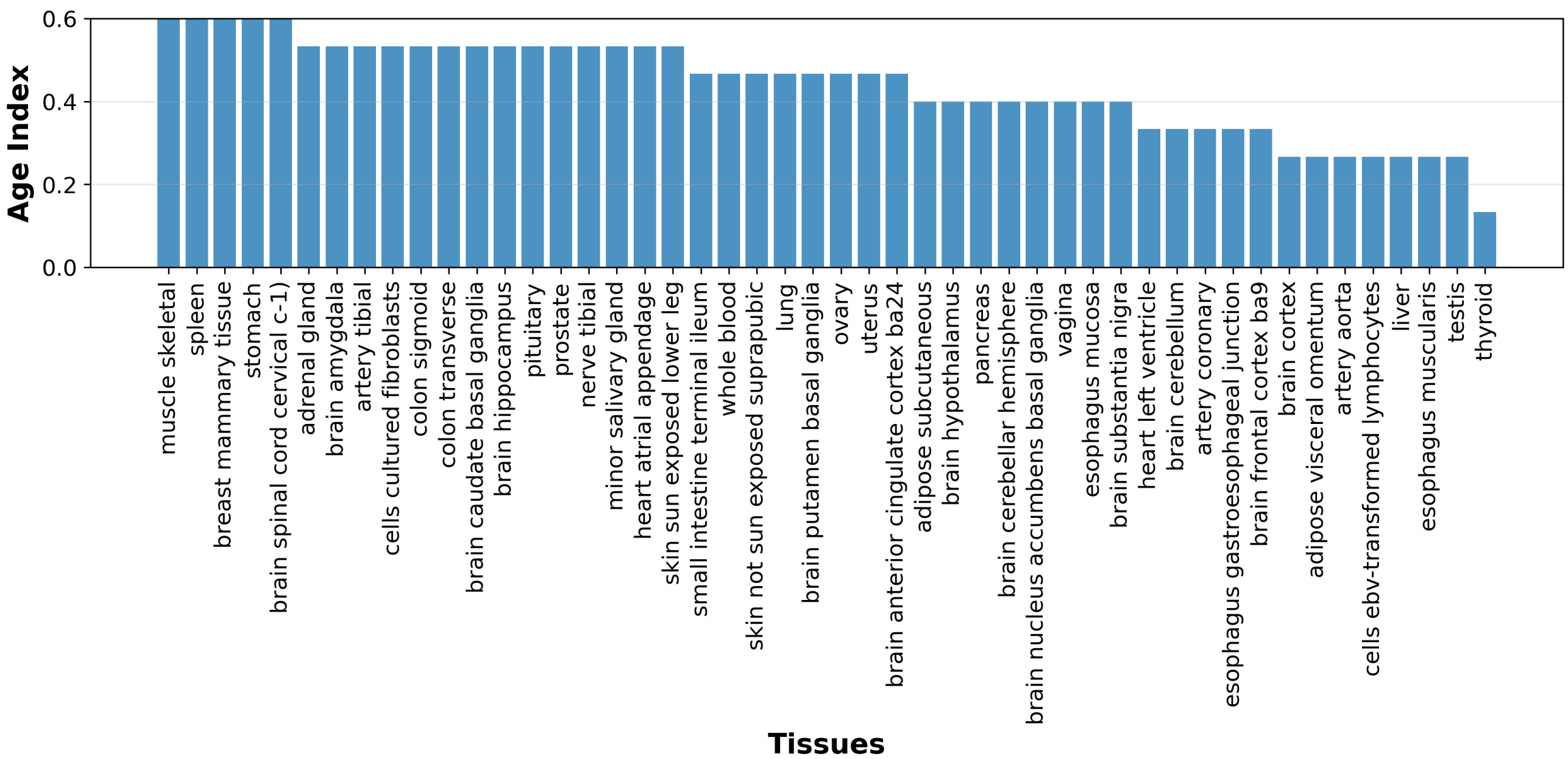

Fig. SM. 16. Maximum Age Index per Tissue for $\lambda = 2.0$, $\epsilon = 0.005$, and $\beta > 0$.

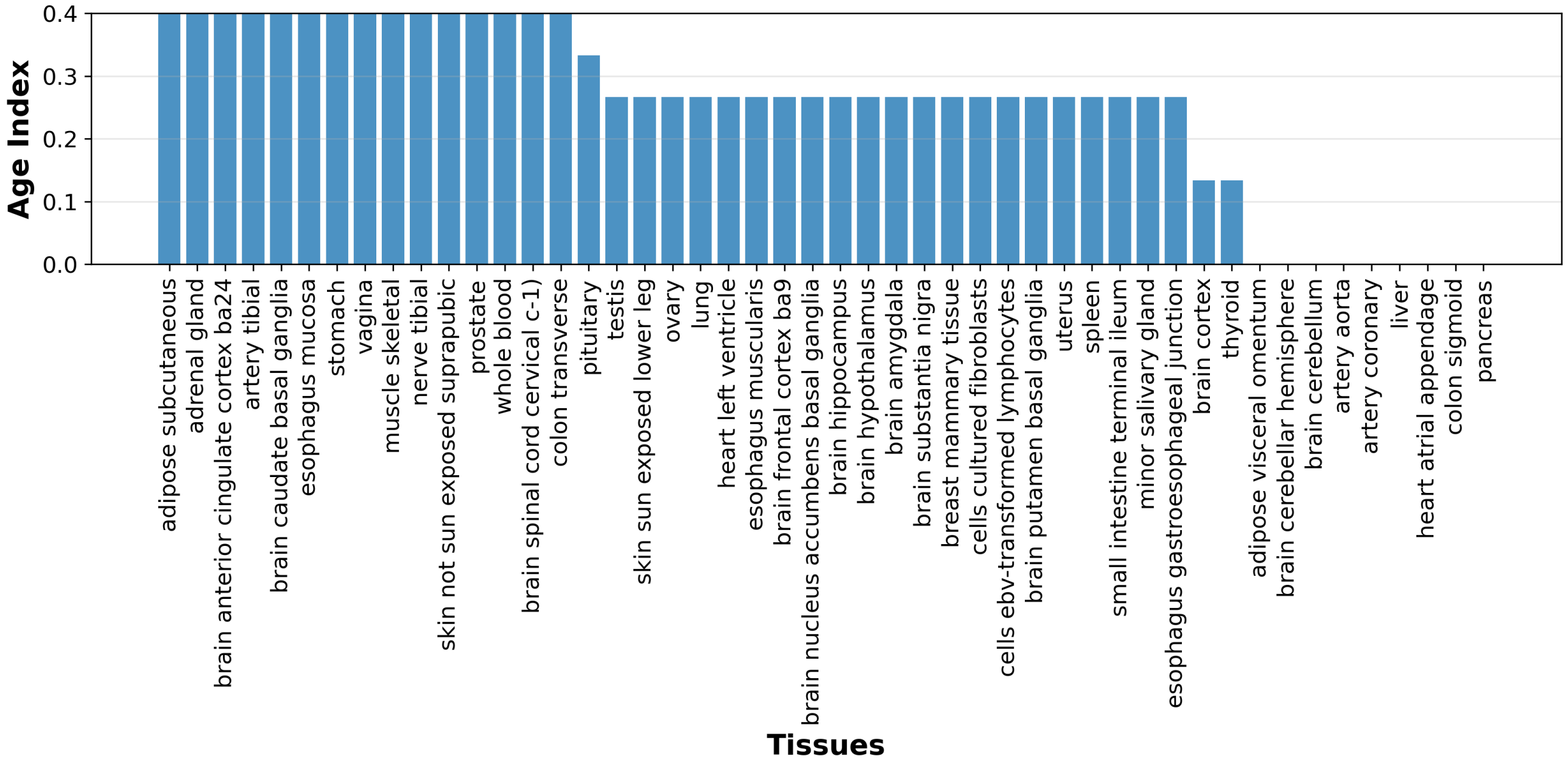

Fig. SM. 17. Maximum Age Index per Tissue for $\lambda = 2.0$, $\epsilon = 0.008$, and $\beta = 0$.

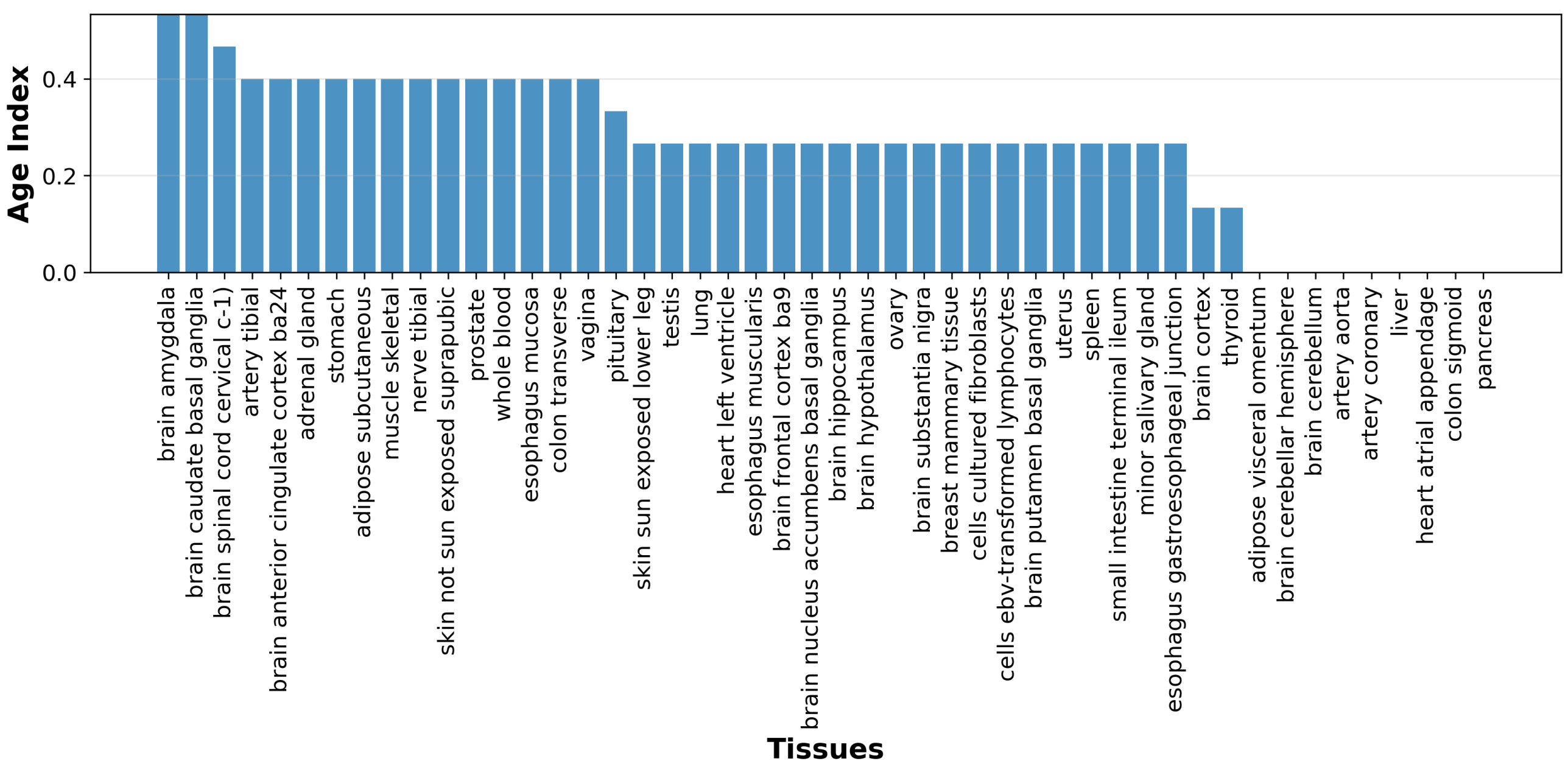

Fig. SM. 18. Maximum Age Index per Tissue for $\lambda = 2.0$, $\epsilon = 0.008$, and $\beta > 0$.

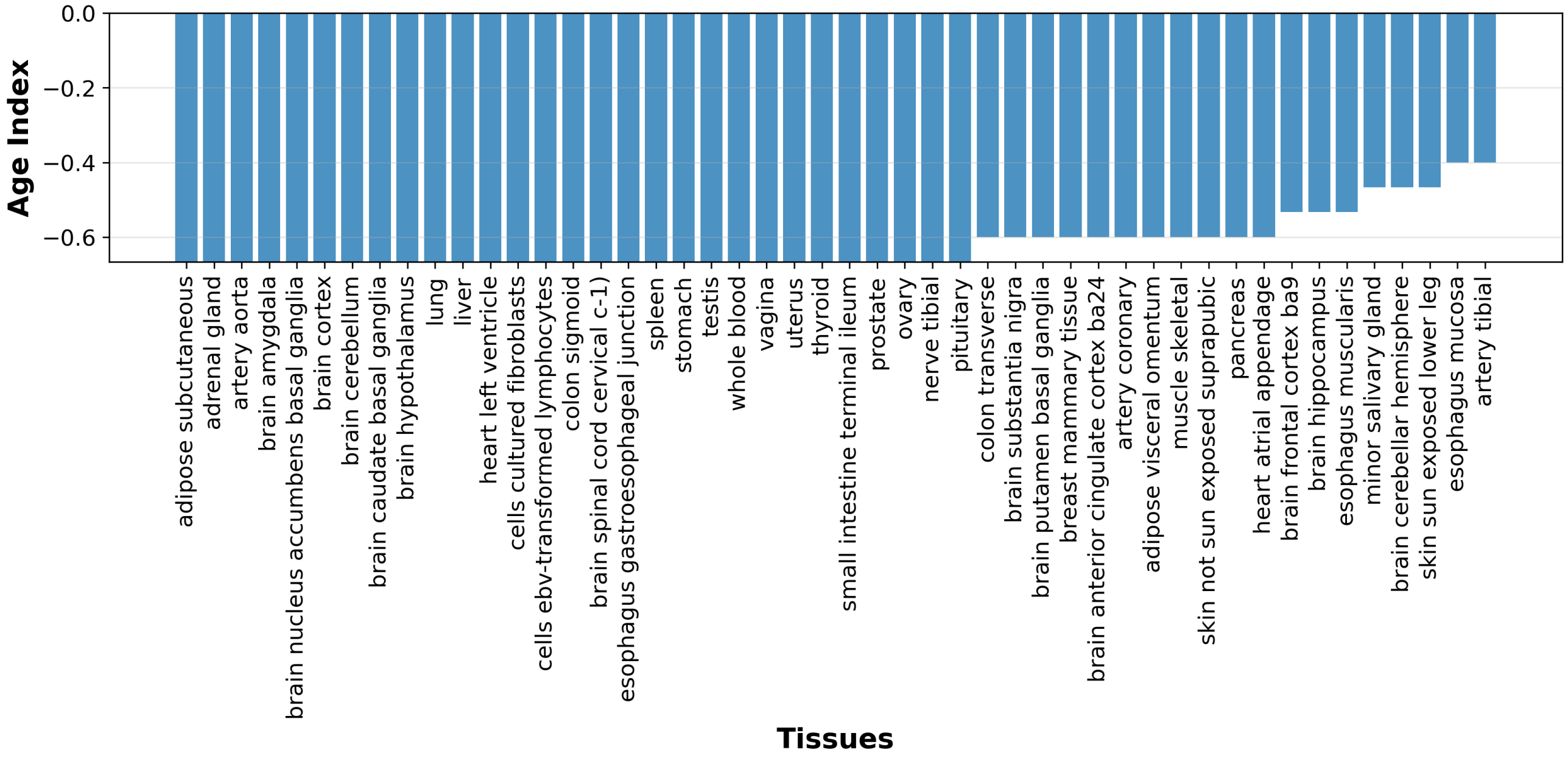

Fig. SM. 19. Minimum Age Index per Tissue for $\lambda = 0.5$, $\epsilon = 0.003$, and $\beta = 0$.

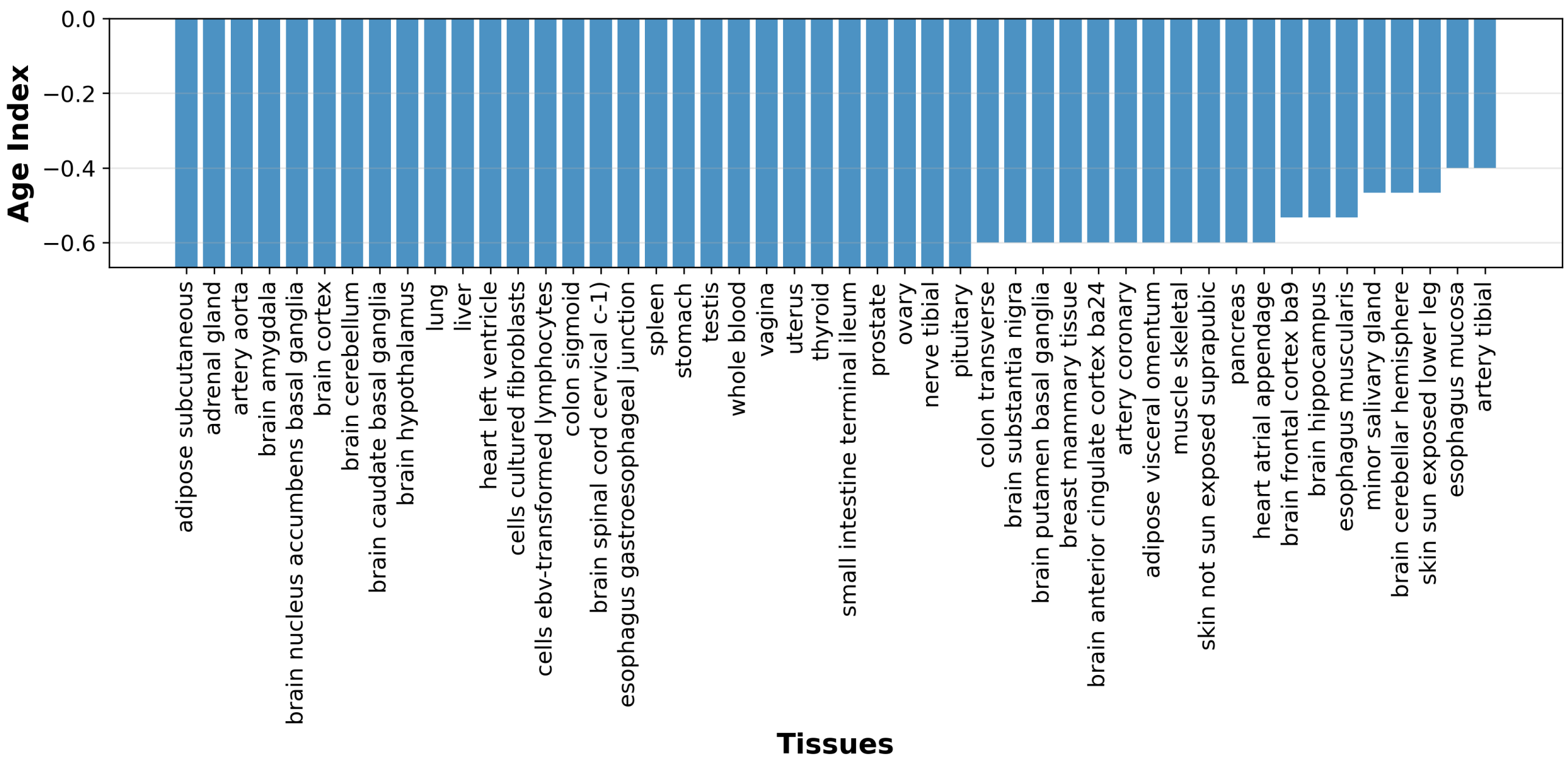

Fig. SM. 20. Minimum Age Index per Tissue for $\lambda = 0.5$, $\epsilon = 0.003$, and $\beta > 0$.

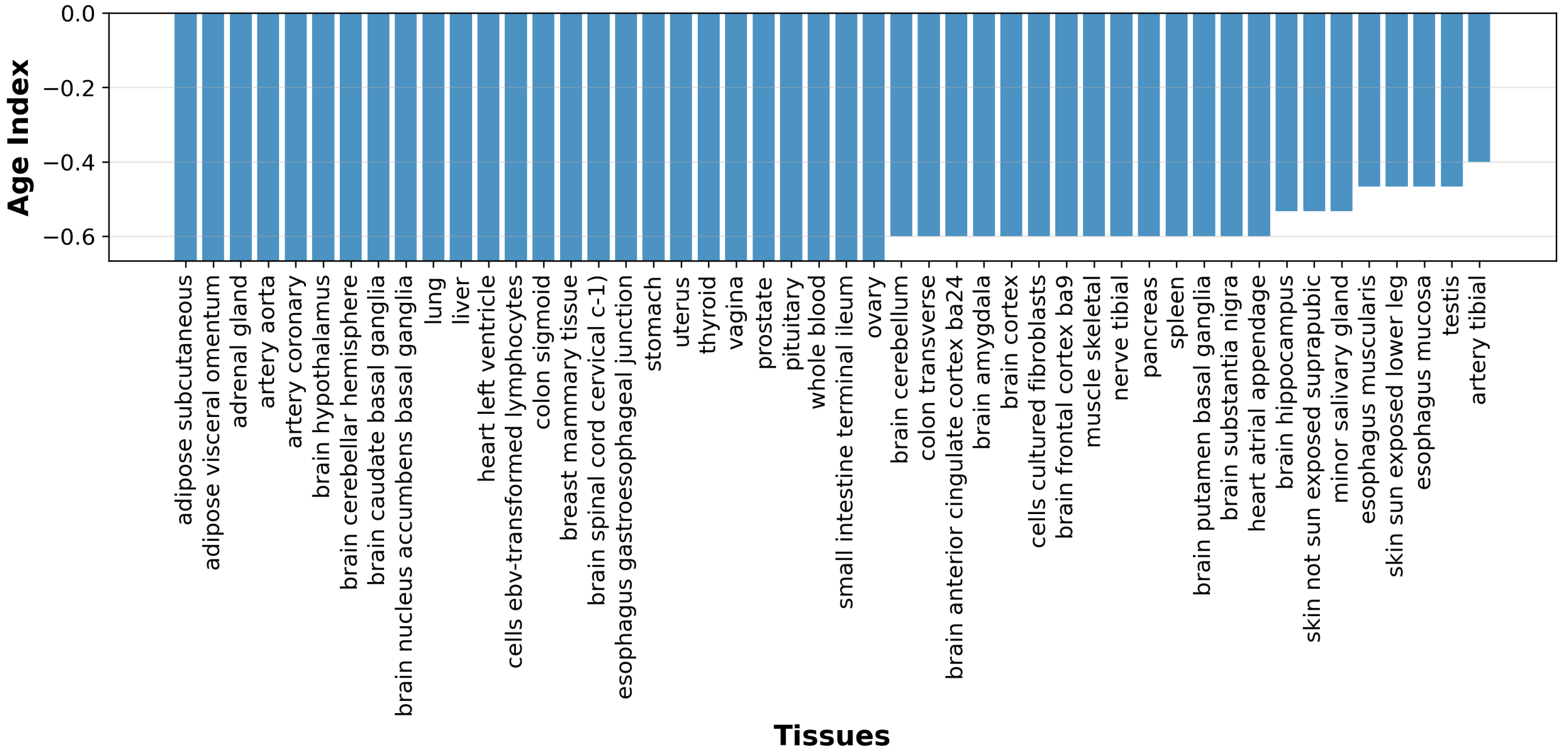

Fig. SM. 21. Minimum Age Index per Tissue for $\lambda = 0.5$, $\epsilon = 0.005$, and $\beta = 0$.

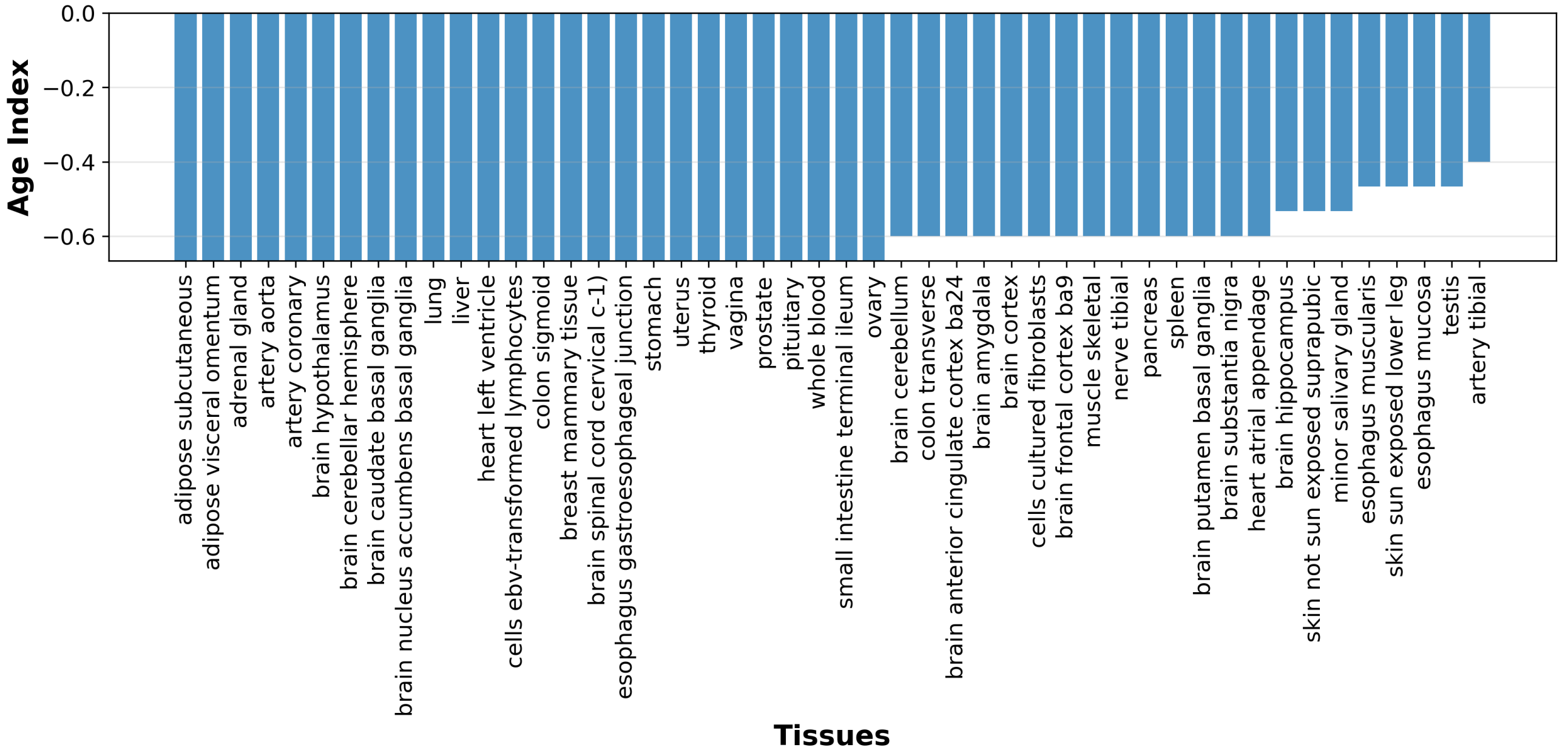

Fig. SM. 22. Minimum Age Index per Tissue for $\lambda = 0.5$, $\epsilon = 0.005$, and $\beta > 0$.

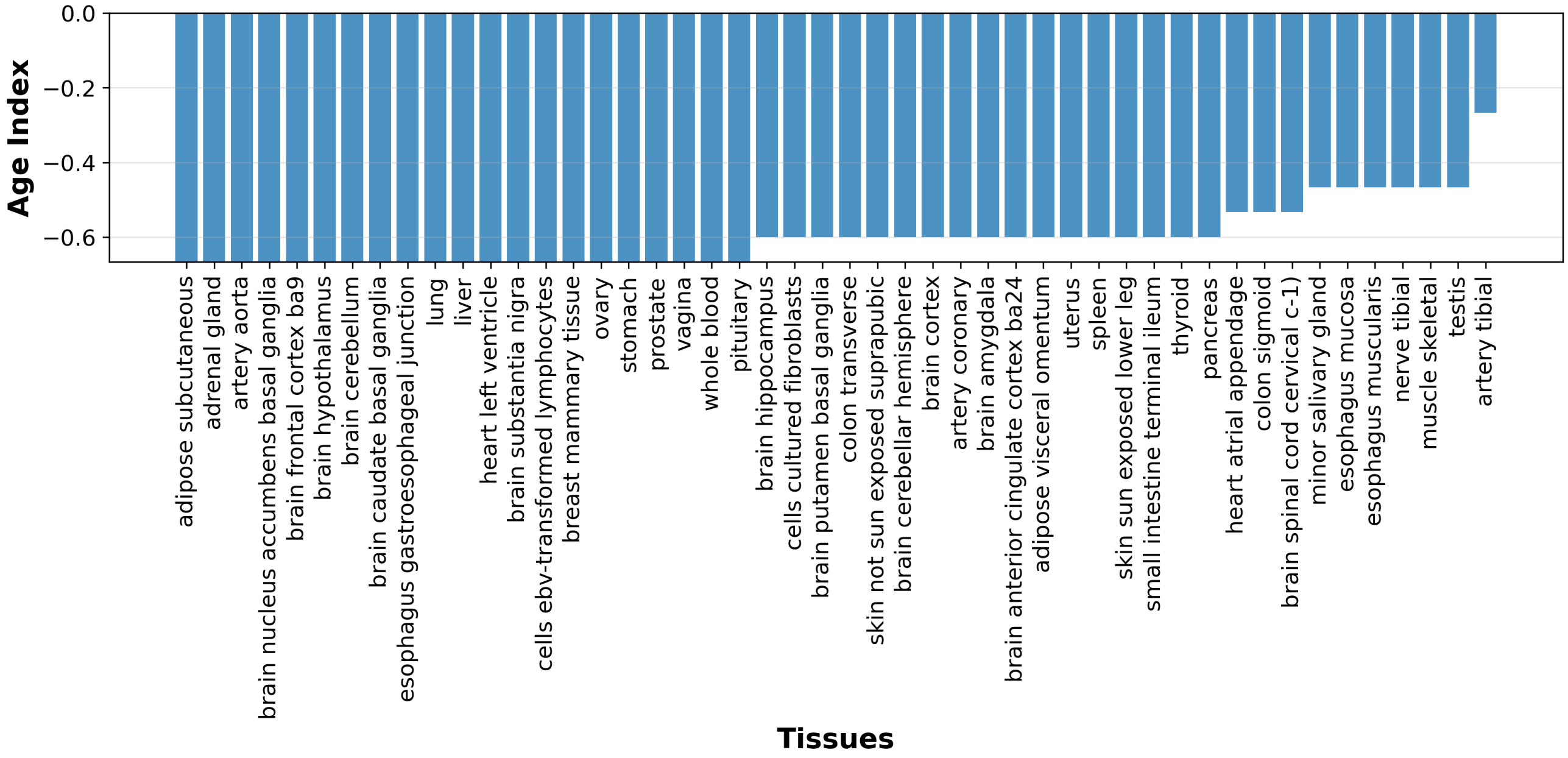

Fig. SM. 23. Minimum Age Index per Tissue for $\lambda = 0.5$, $\epsilon = 0.008$, and $\beta = 0$.

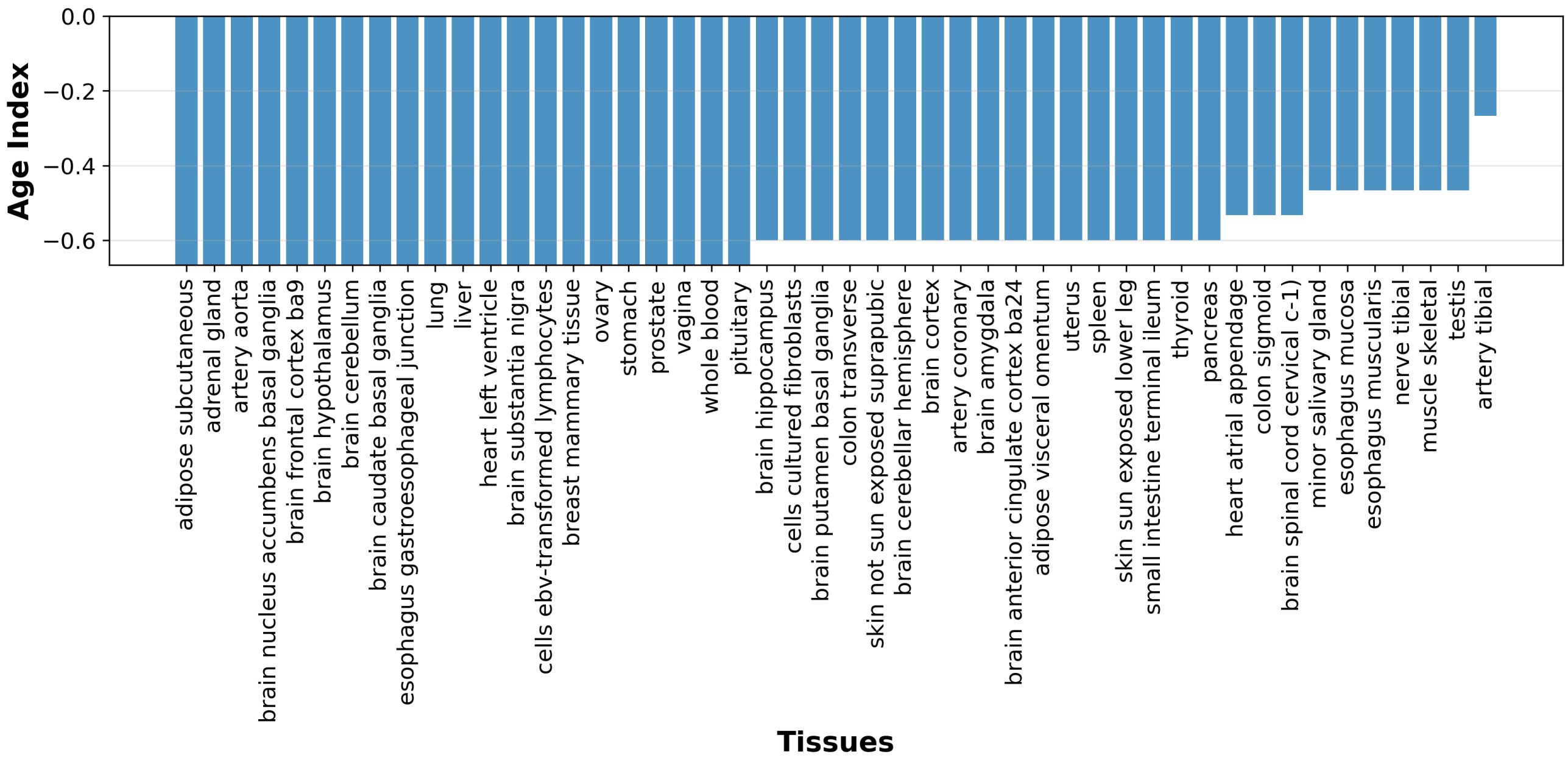

Fig. SM. 24. Minimum Age Index per Tissue for $\lambda = 0.5$, $\epsilon = 0.008$, and $\beta > 0$.

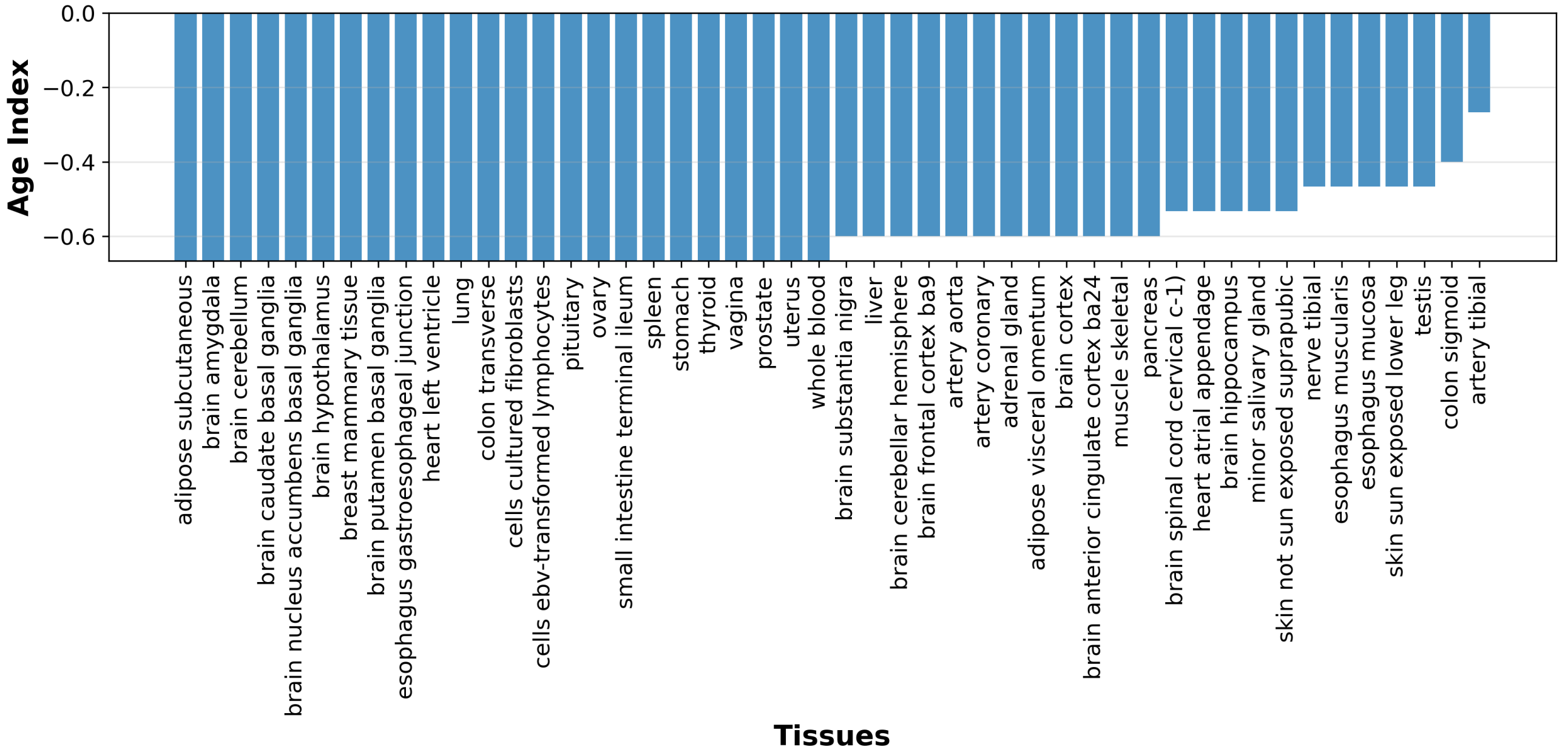

Fig. SM. 25. Minimum Age Index per Tissue for $\lambda = 1.0$, $\epsilon = 0.003$, and $\beta = 0$.

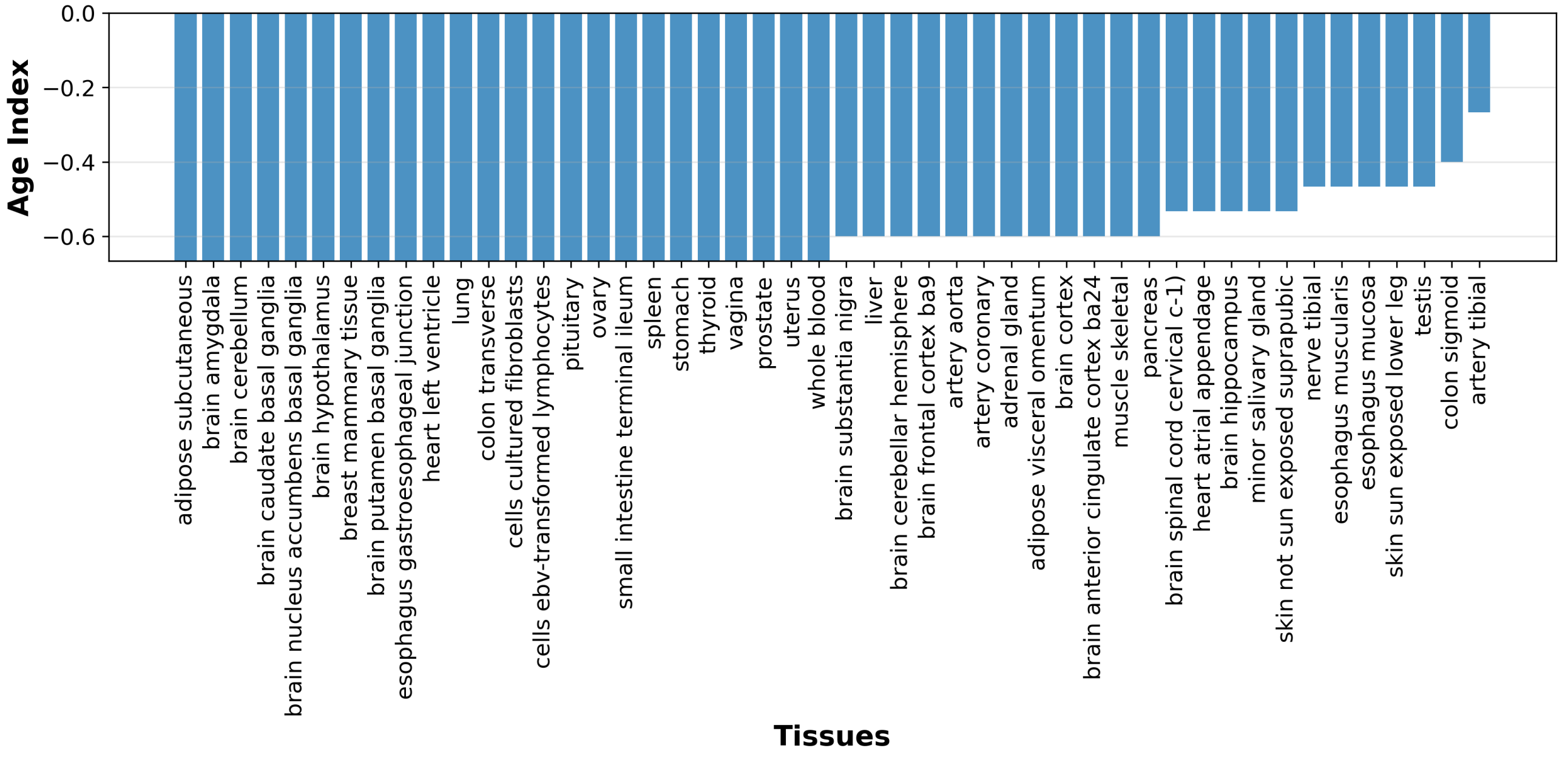

Fig. SM. 26. Minimum Age Index per Tissue for $\lambda = 1.0$, $\epsilon = 0.003$, and $\beta > 0$.

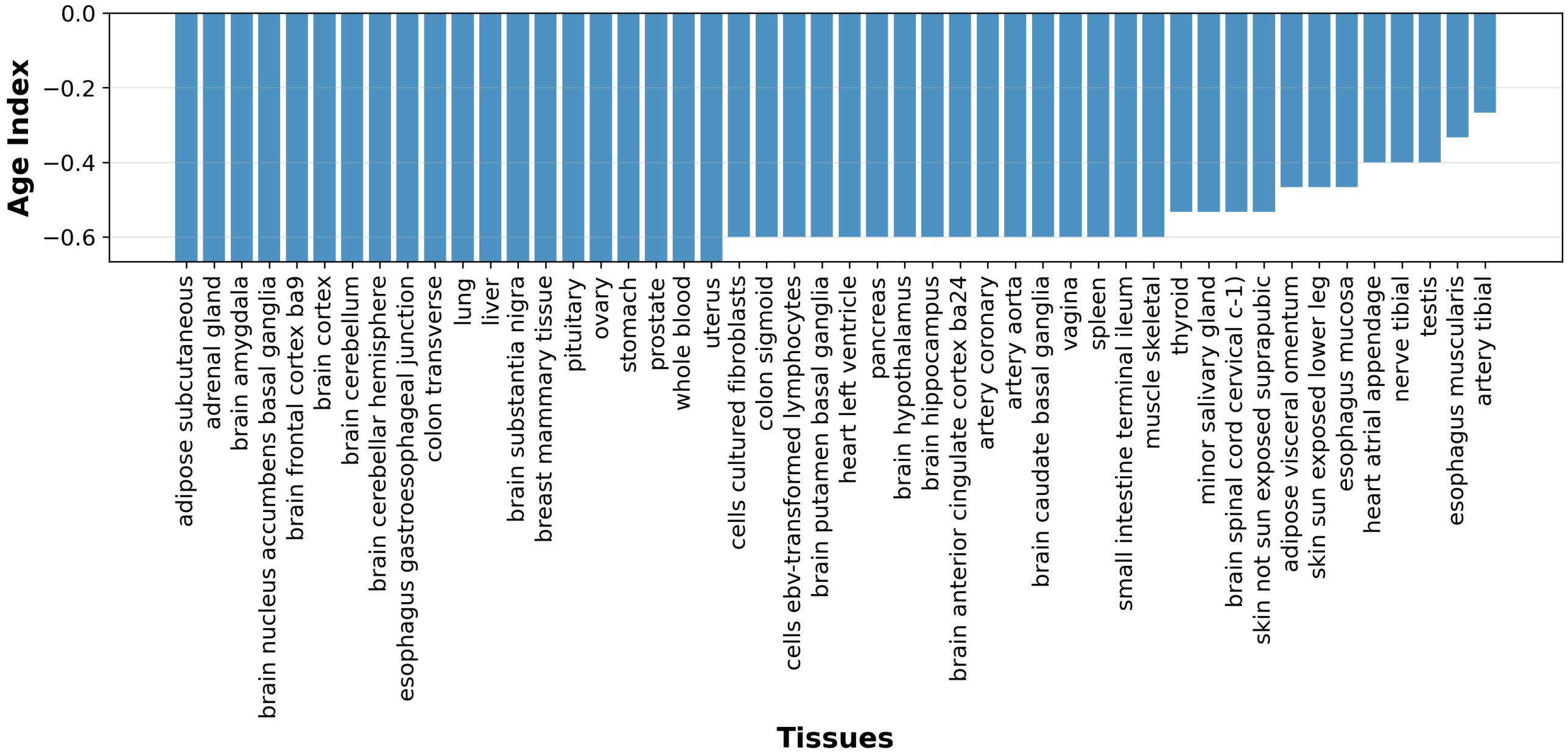

Fig. SM. 27. Minimum Age Index per Tissue for $\lambda = 1.0$, $\epsilon = 0.005$, and $\beta = 0$.

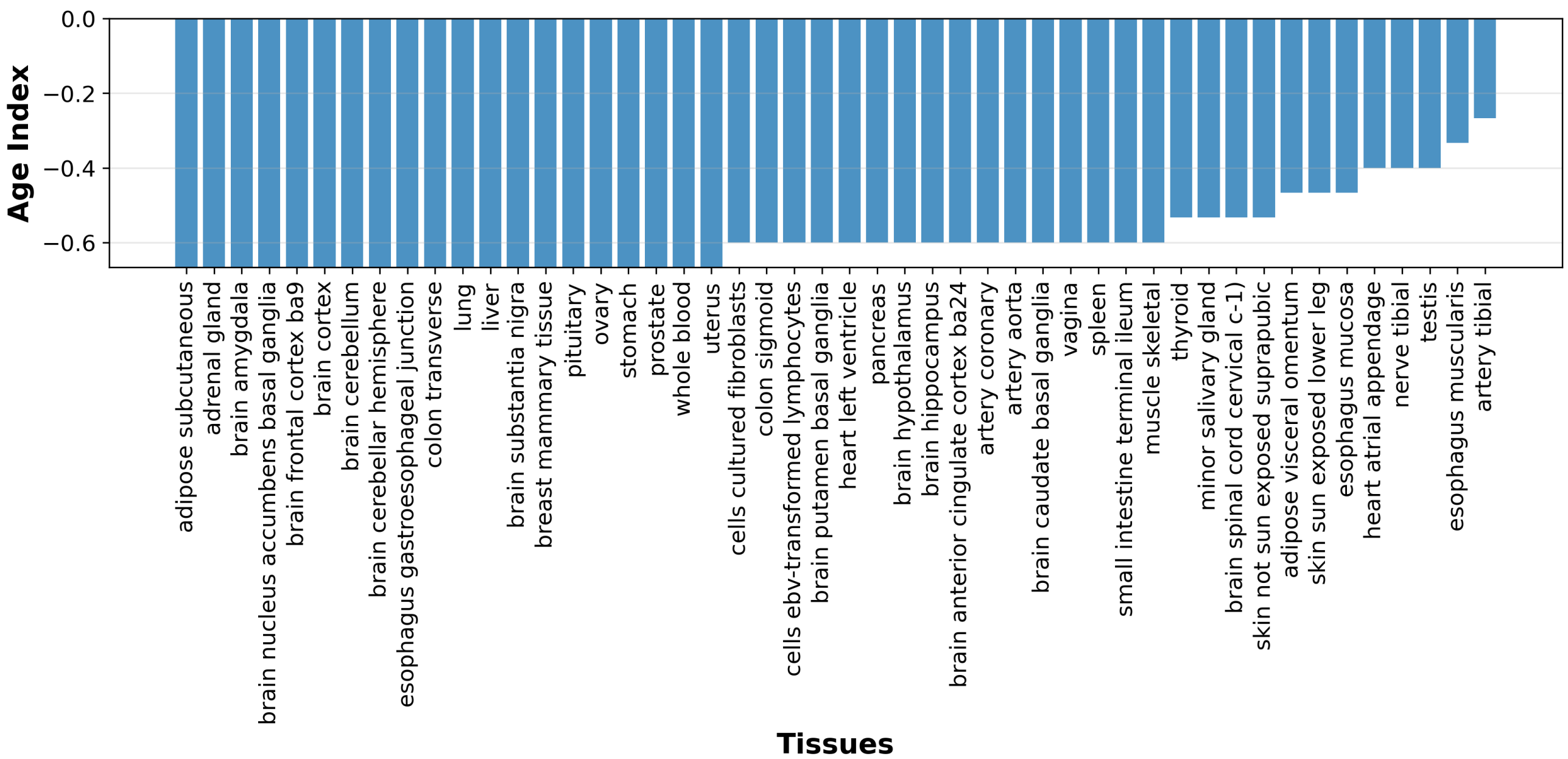

Fig. SM. 28. Minimum Age Index per Tissue for $\lambda = 1.0$, $\epsilon = 0.005$, and $\beta > 0$.

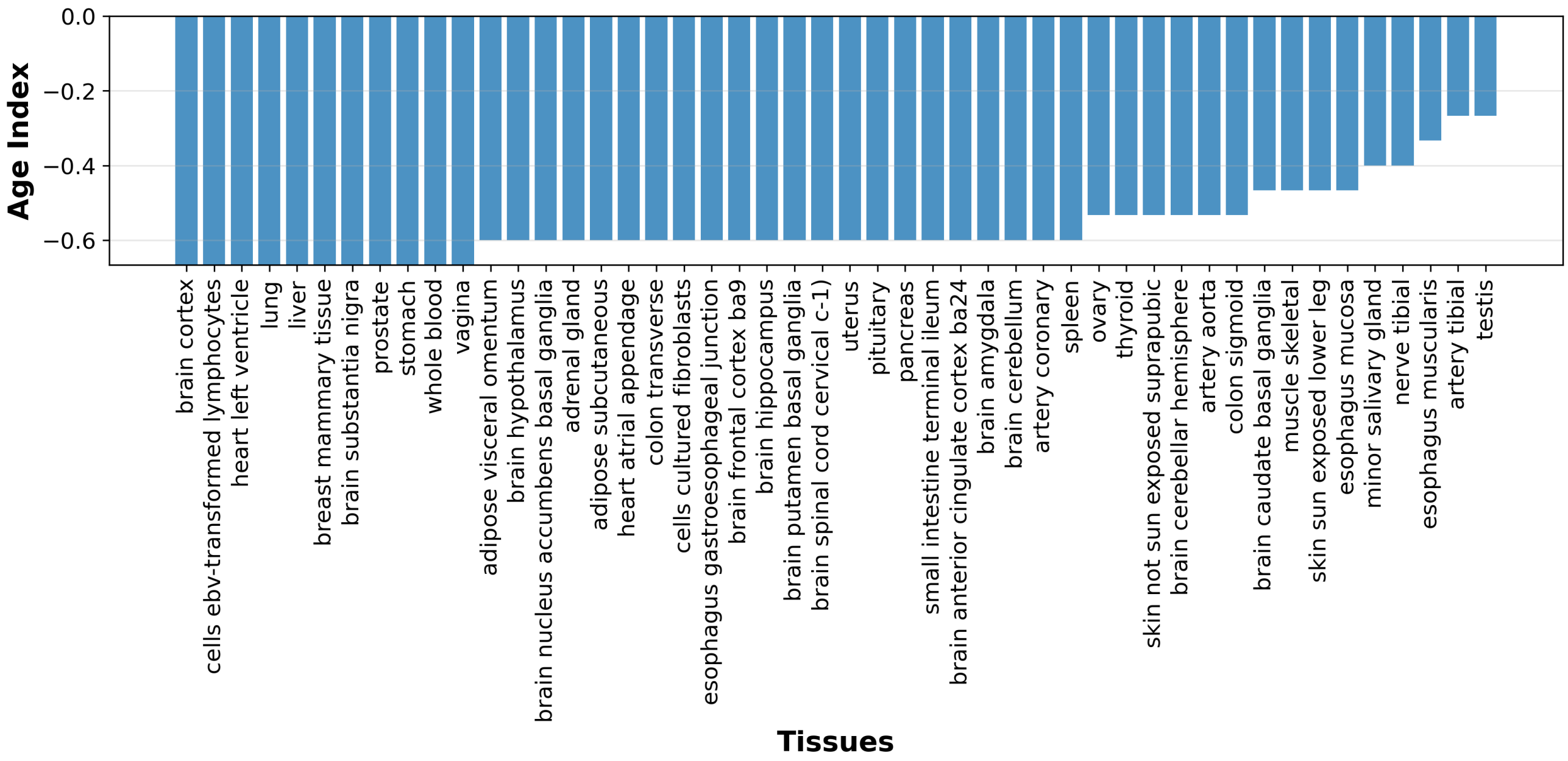

Fig. SM. 29. Minimum Age Index per Tissue for $\lambda = 1.0$, $\epsilon = 0.008$, and $\beta = 0$.

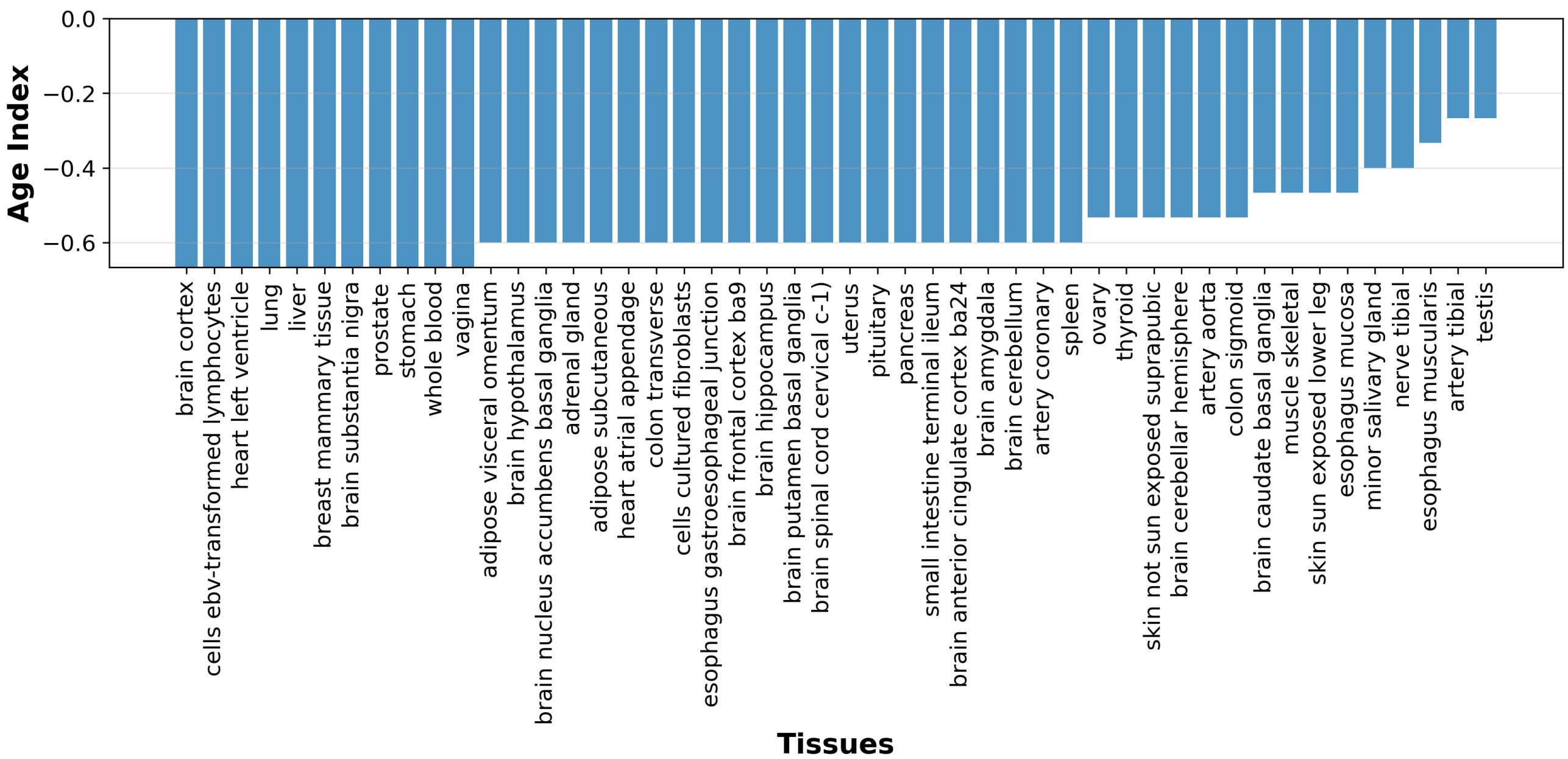

Fig. SM. 30. Minimum Age Index per Tissue for $\lambda = 1.0$, $\epsilon = 0.008$, and $\beta > 0$.

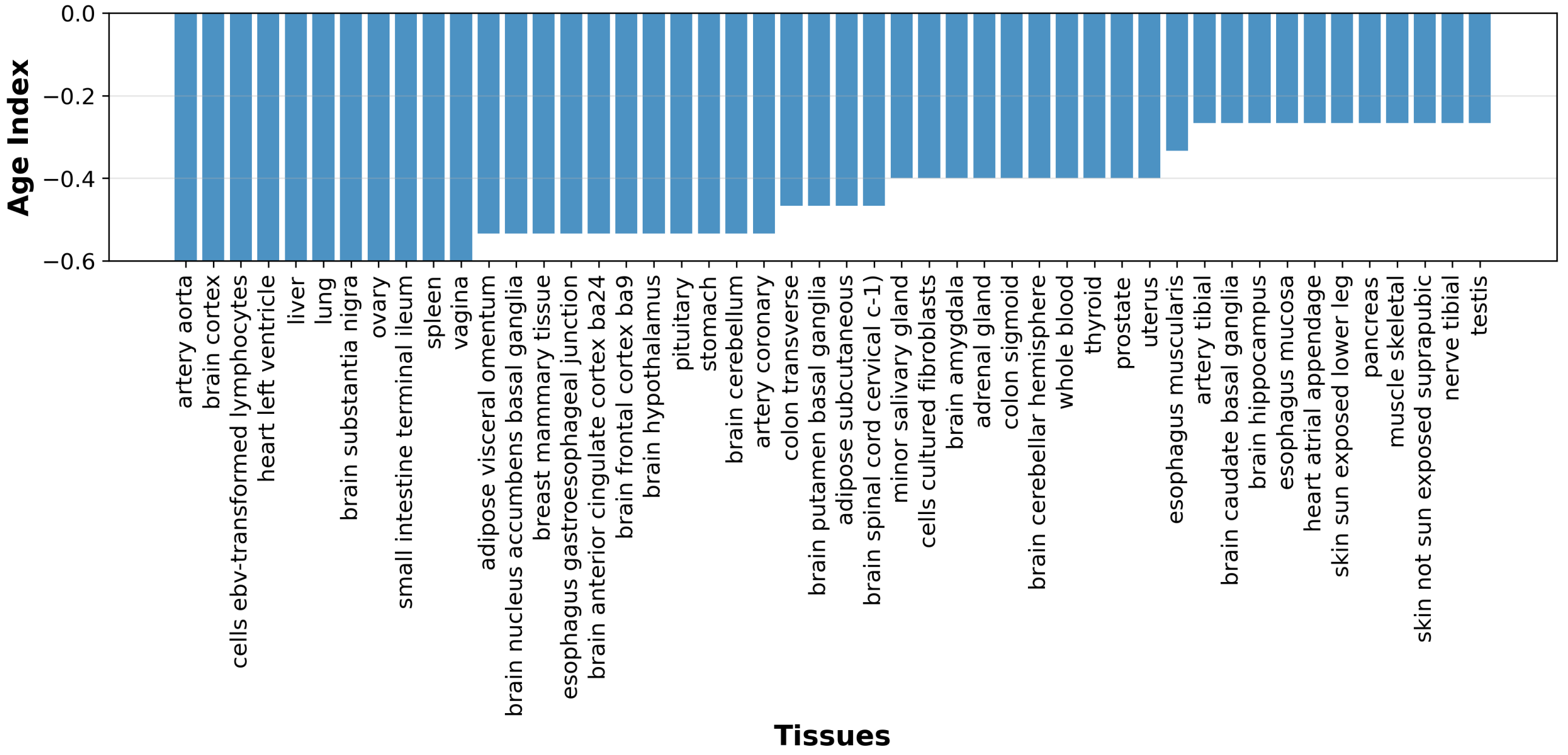

Fig. SM. 31. Minimum Age Index per Tissue for $\lambda = 2.0$, $\epsilon = 0.003$, and $\beta = 0$.

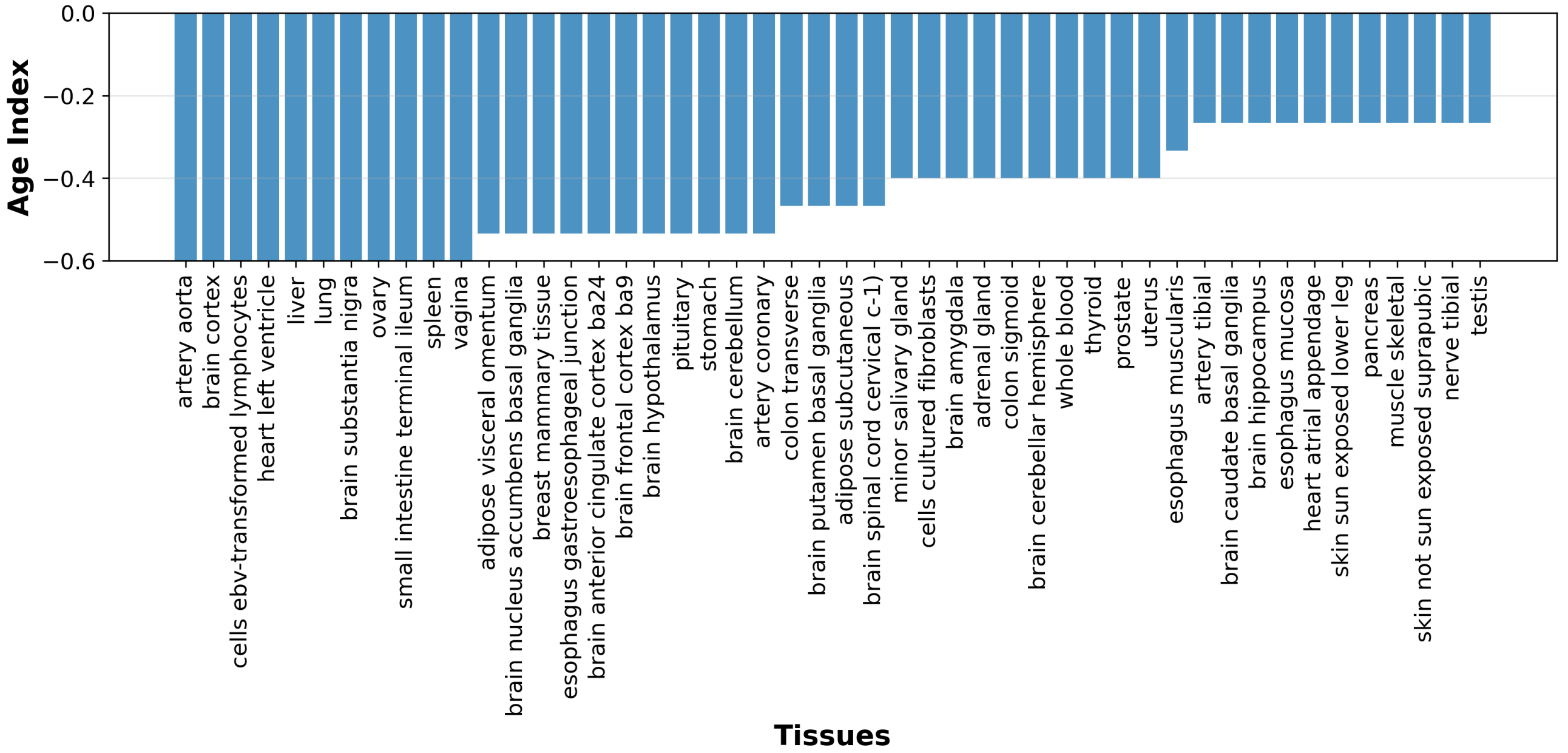

Fig. SM. 32. Minimum Age Index per Tissue for $\lambda = 2.0$, $\epsilon = 0.003$, and $\beta > 0$.

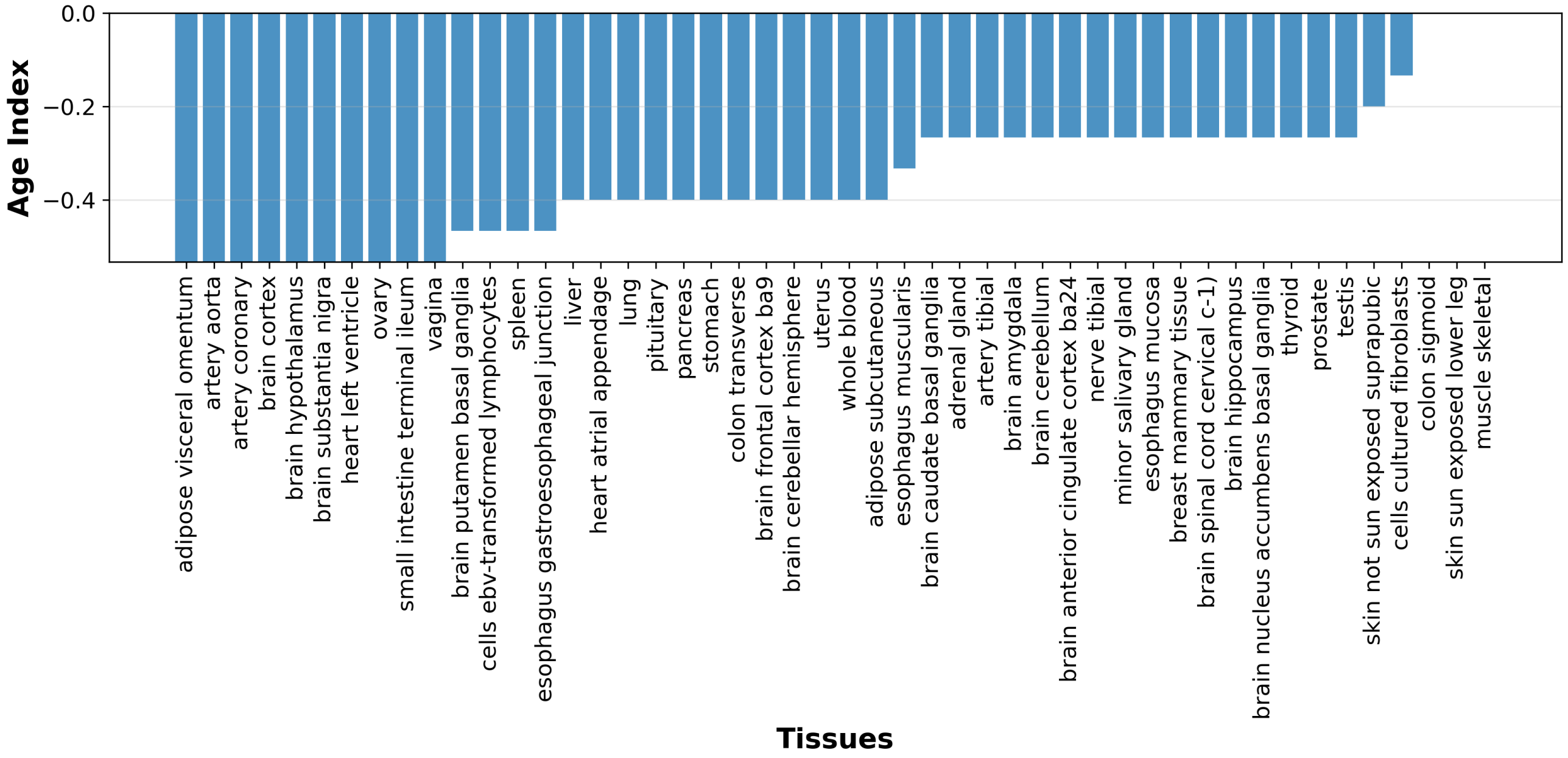

Fig. SM. 33. Minimum Age Index per Tissue for $\lambda = 2.0$, $\epsilon = 0.005$, and $\beta = 0$.

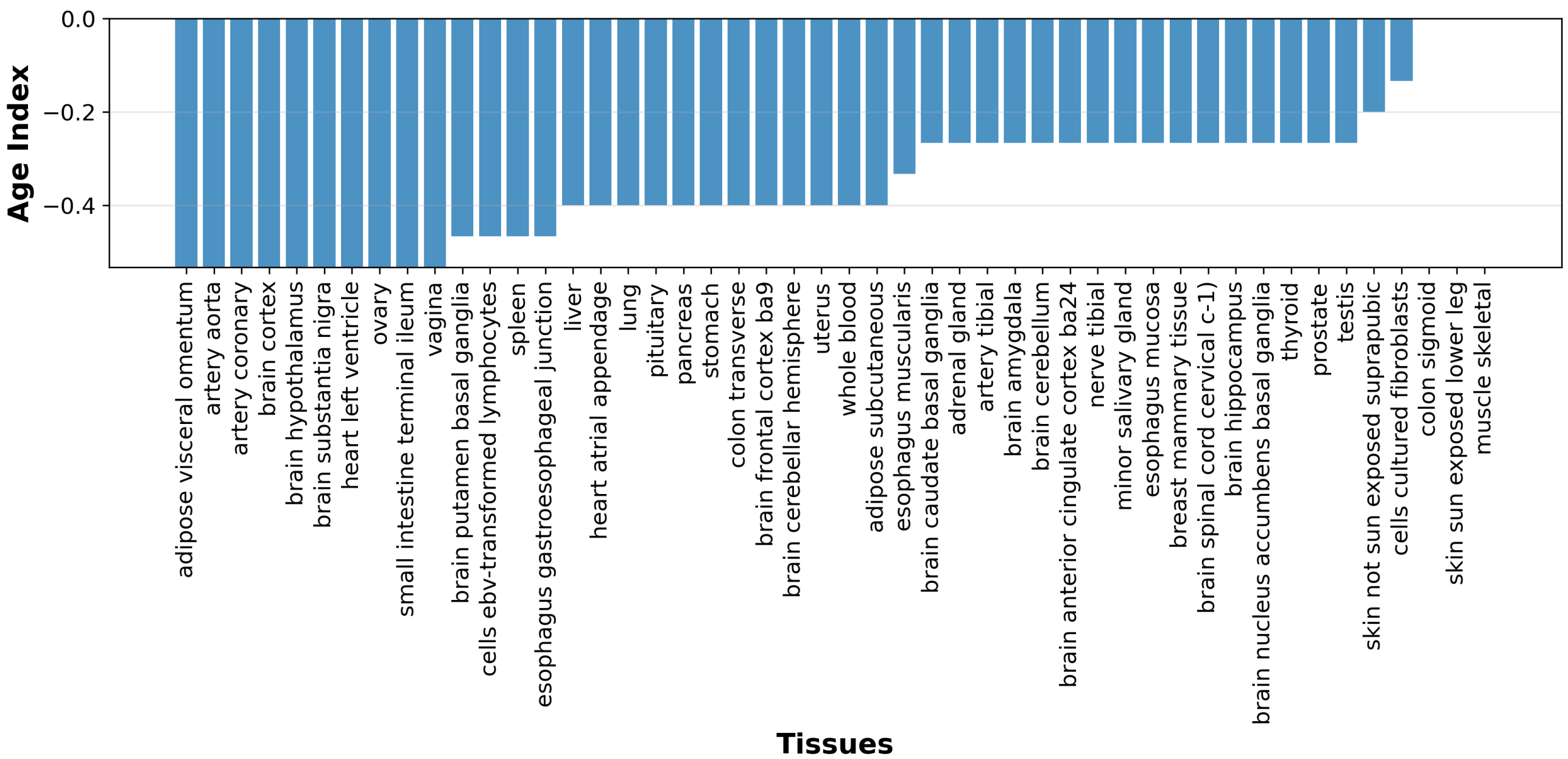

Fig. SM. 34. Minimum Age Index per Tissue for $\lambda = 2.0$, $\epsilon = 0.005$, and $\beta > 0$.

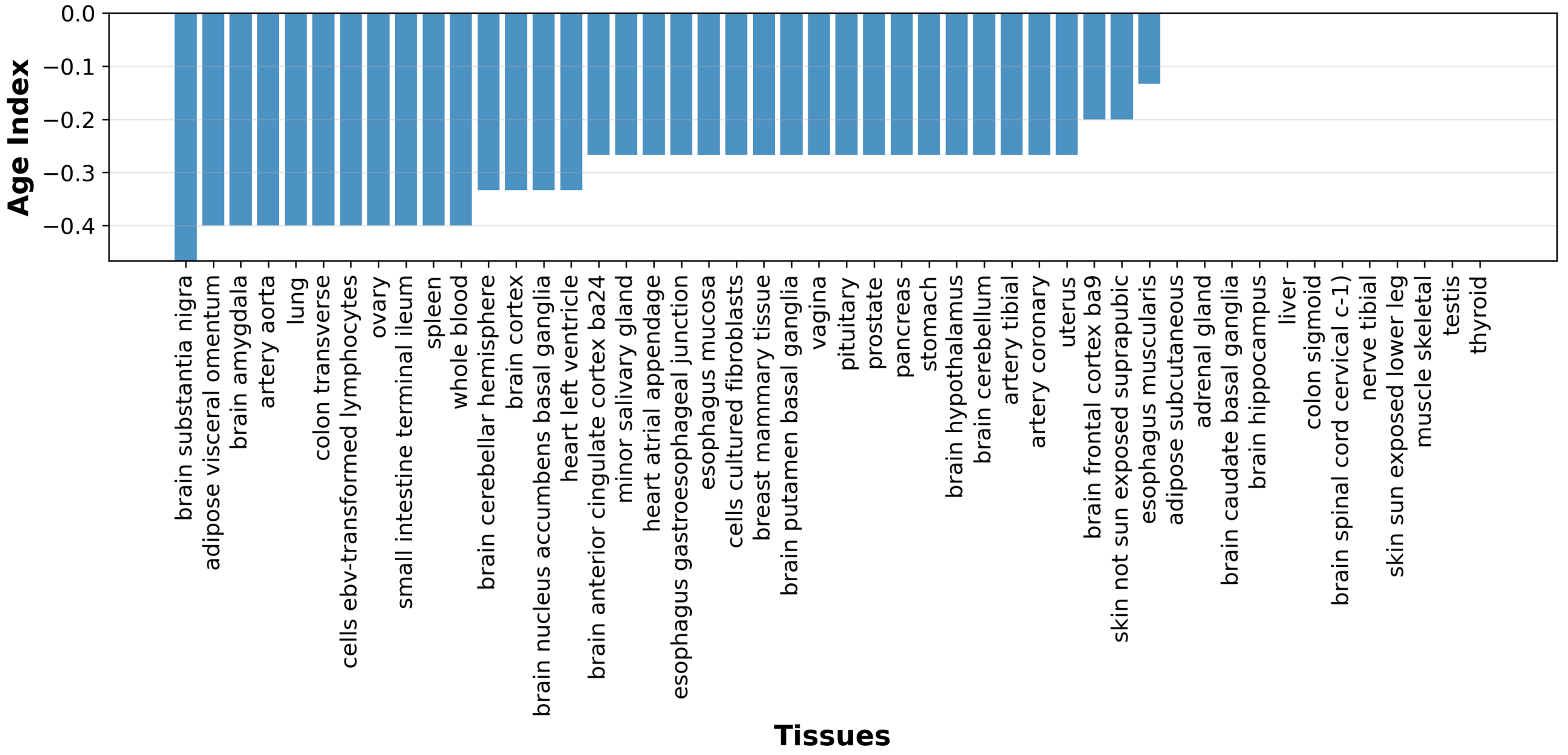

Fig. SM. 35. Minimum Age Index per Tissue for $\lambda = 2.0$, $\epsilon = 0.008$, and $\beta = 0$.

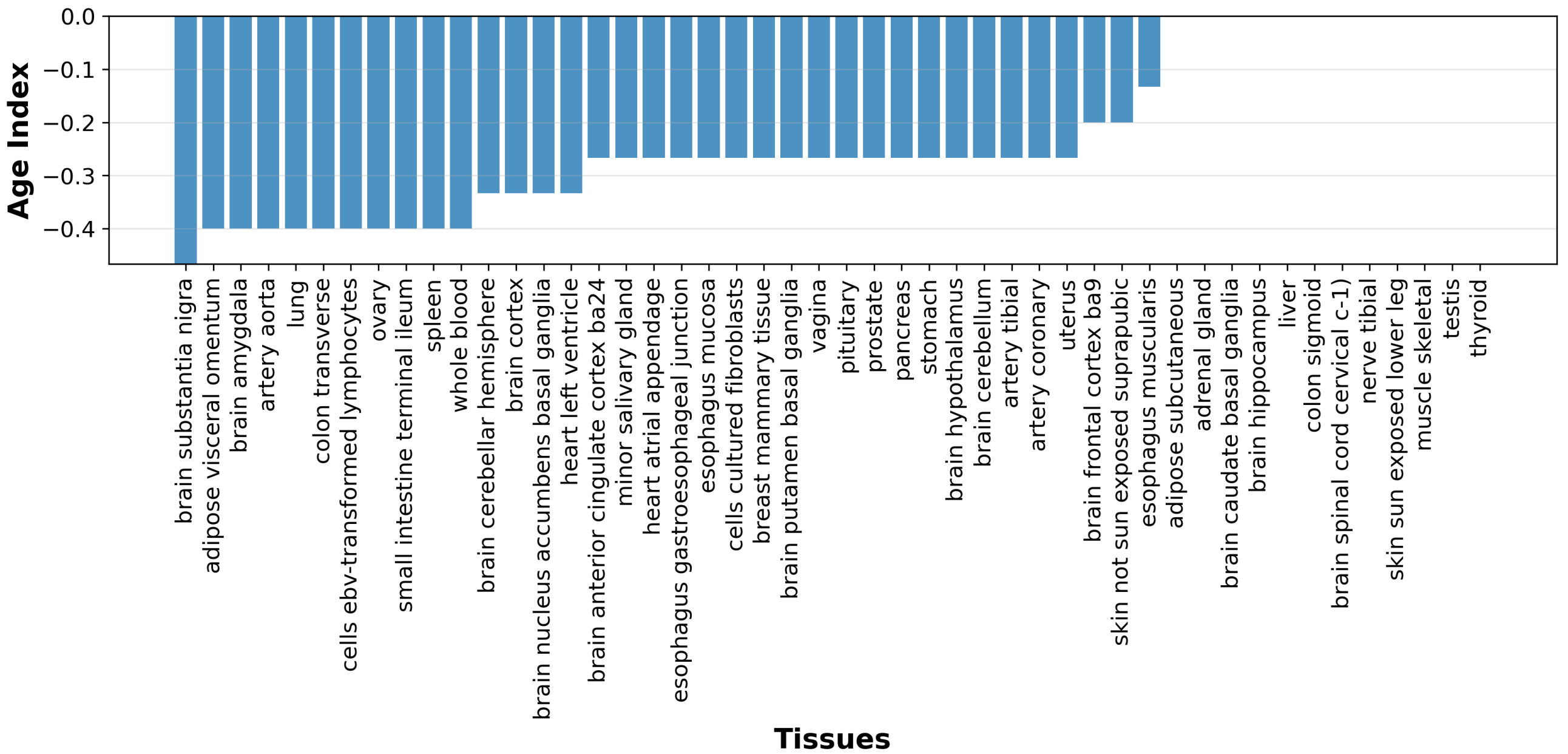

Fig. SM. 36. Minimum Age Index per Tissue for $\lambda = 2.0$, $\epsilon = 0.008$, and $\beta > 0$.

Robustness of Tissue-wise Age Index under Edge Rewiring Perturbations

Tissue-wise Maximal/Minimal Age Index under controlled network perturbations ($p \in \{1\%, 2\%, 4\%, 5\%, 10\%\}$). The Age Index was computed using $\lambda = 0.3$, $\epsilon = 0.005$, and $\beta > 0$, based on $n = 10$ independent stochastic runs. For all Supplementary Figures SM.37–SM.66, bars represent the arithmetic mean and error bars denote ± one sample standard deviation across the 10 independent runs.

Perturbed networks were generated using three types of controlled edge modifications applied to $p\%$ of the total number of edges: (i) **Rewiring**: for existing edges $a \to b$ and $c \to d$, the connections were replaced with $a \to d$ and $c \to b$, while preserving the total number of edges in the network; (ii) **Insertion**: new directed edges $u \to v$ were added between randomly selected node pairs without an existing connection, increasing the total number of edges while maintaining the directed structure of the network; (iii) **Deletion**: a randomly selected subset of existing directed edges $u \to v$ was removed, reducing the total number of edges while preserving the overall directed topology of the network.

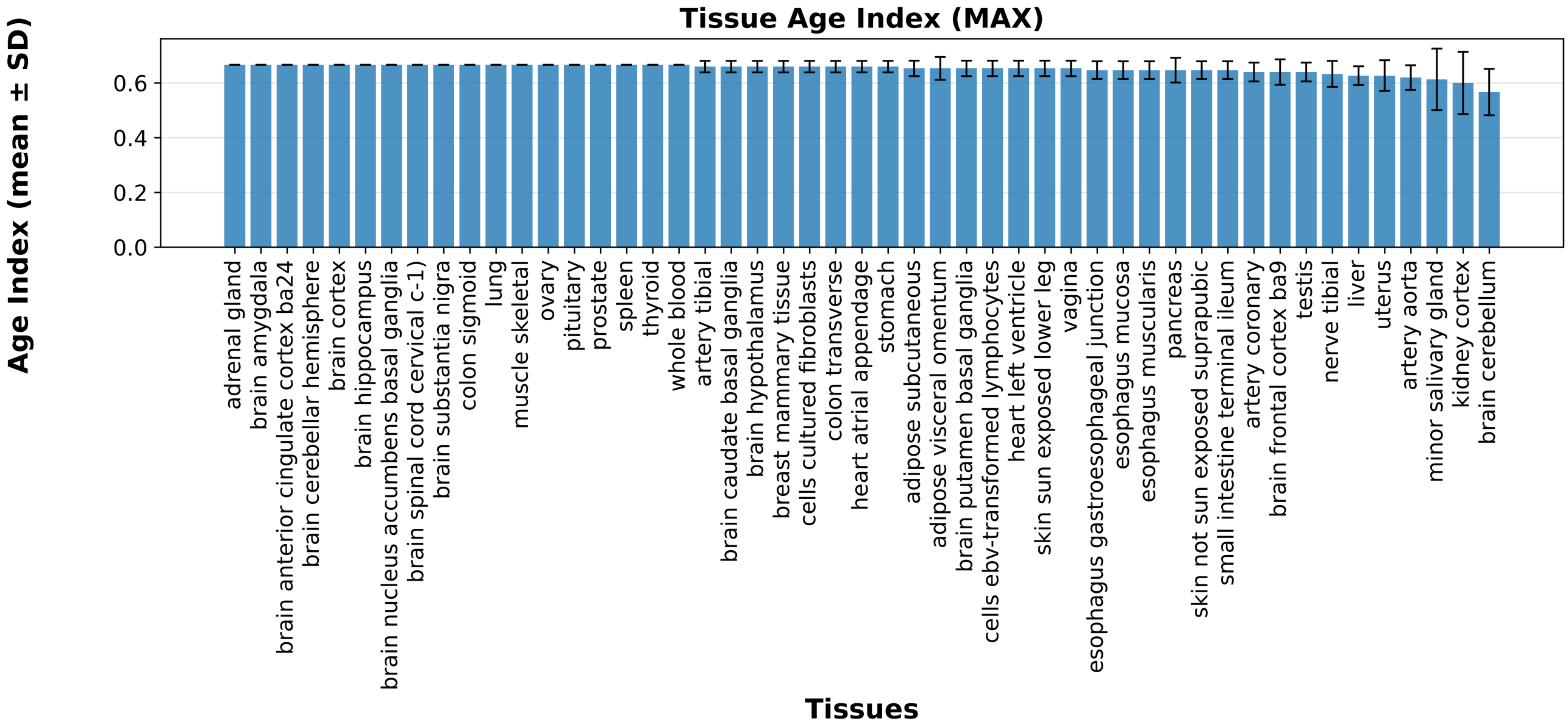

Fig. SM. 37. Tissue-wise Age Index (MAX) under INSERT perturbation ($p = 1\%$).

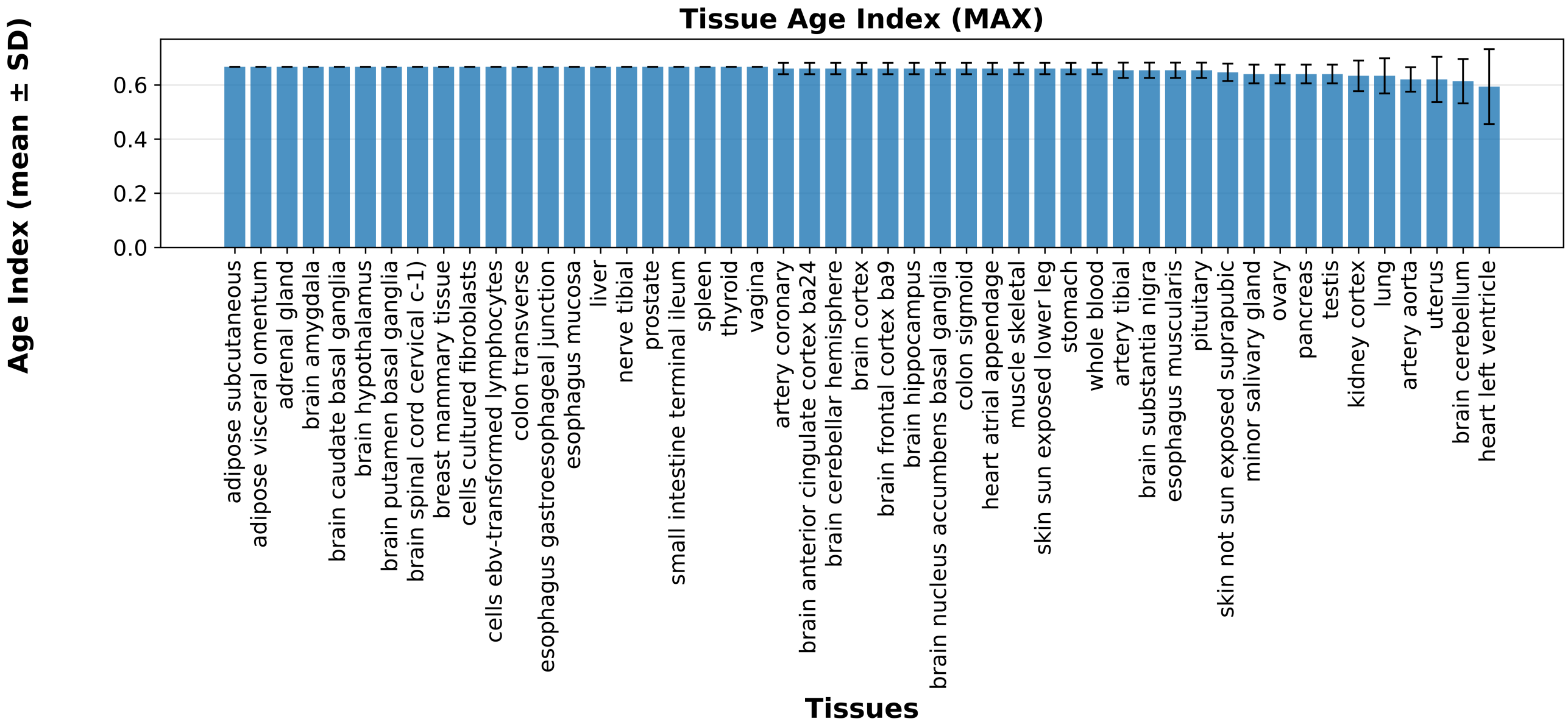

Fig. SM. 38. Tissue-wise Age Index (MAX) under INSERT perturbation ($p = 2\%$).

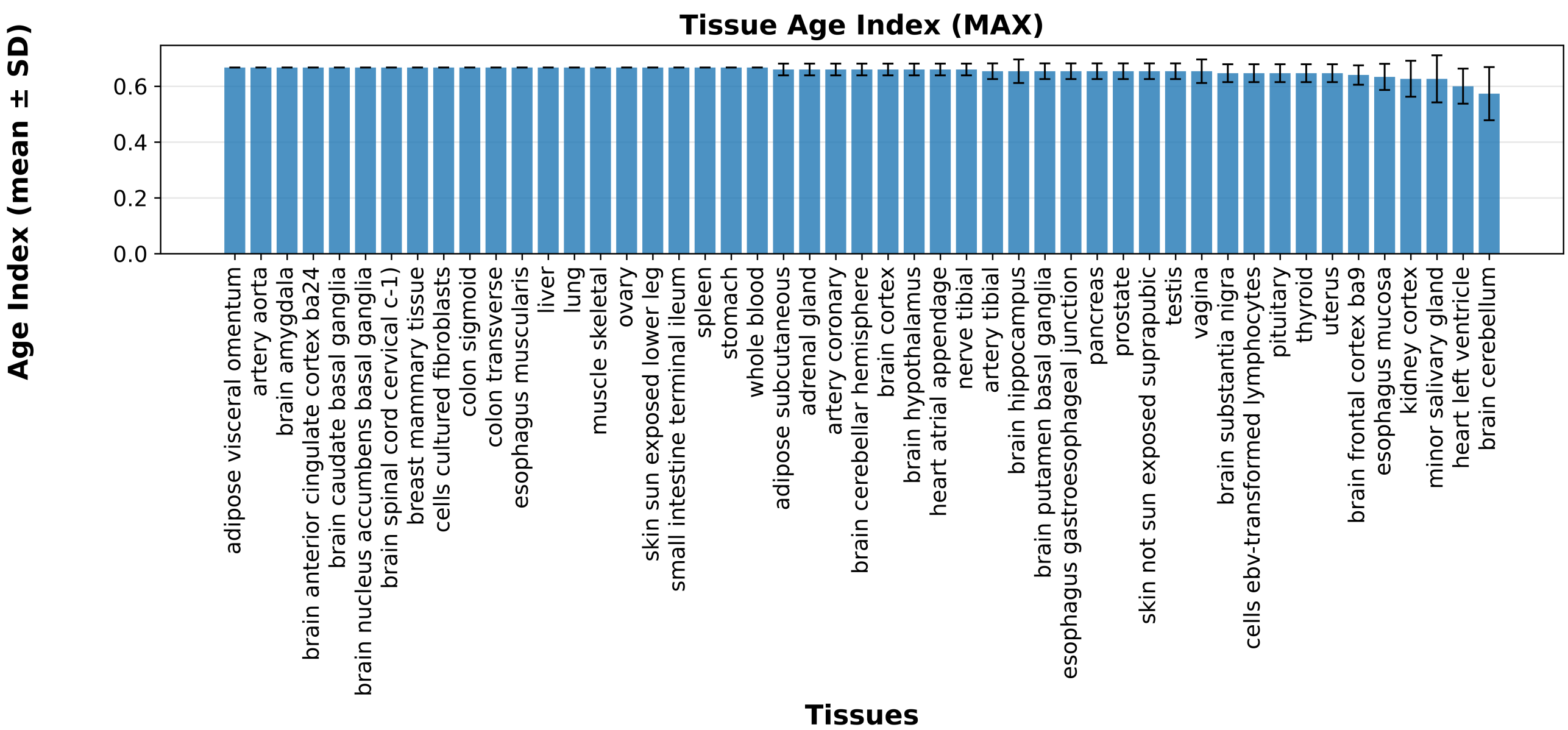

Fig. SM. 39. Tissue-wise Age Index (MAX) under INSERT perturbation ($p = 4\%$).

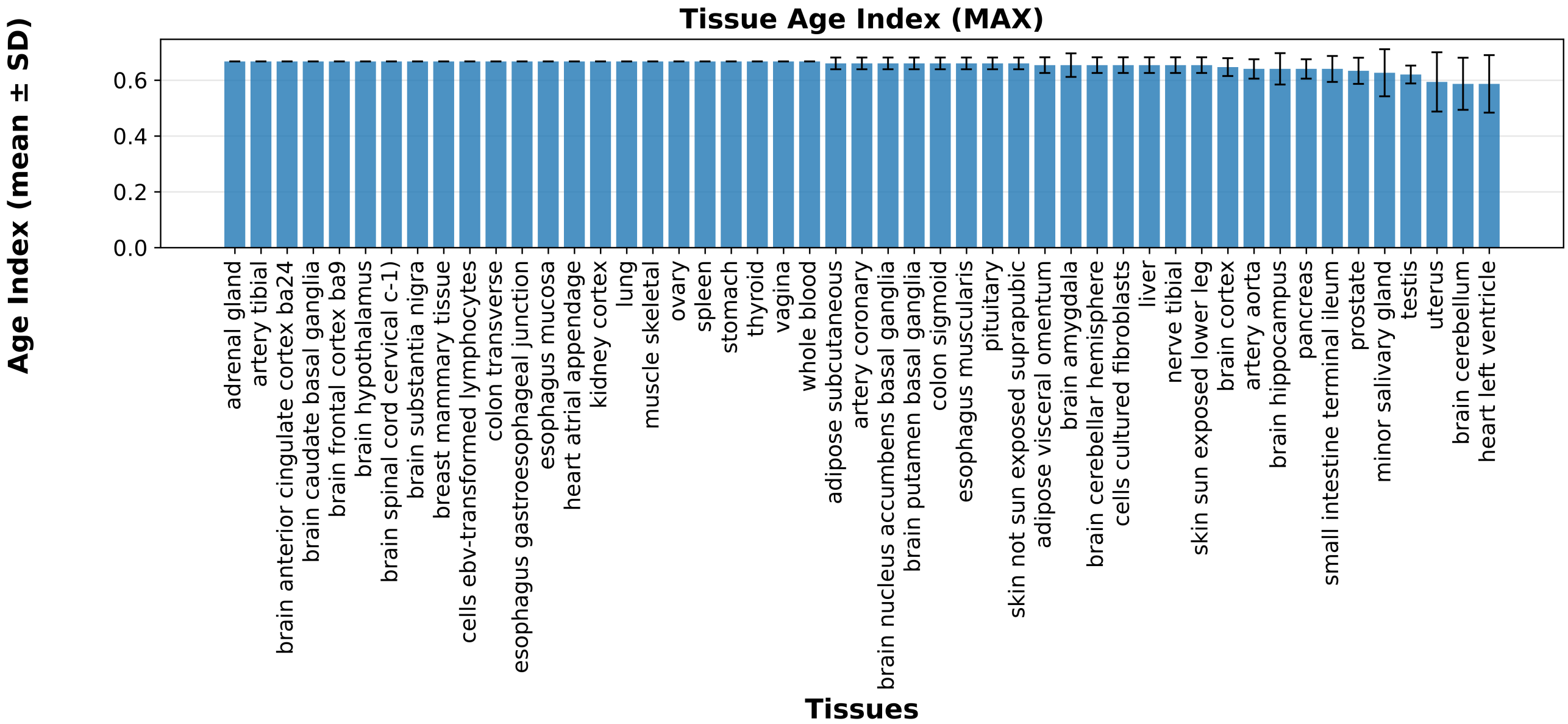

Fig. SM. 40. Tissue-wise Age Index (MAX) under INSERT perturbation ($p = 5\%$).

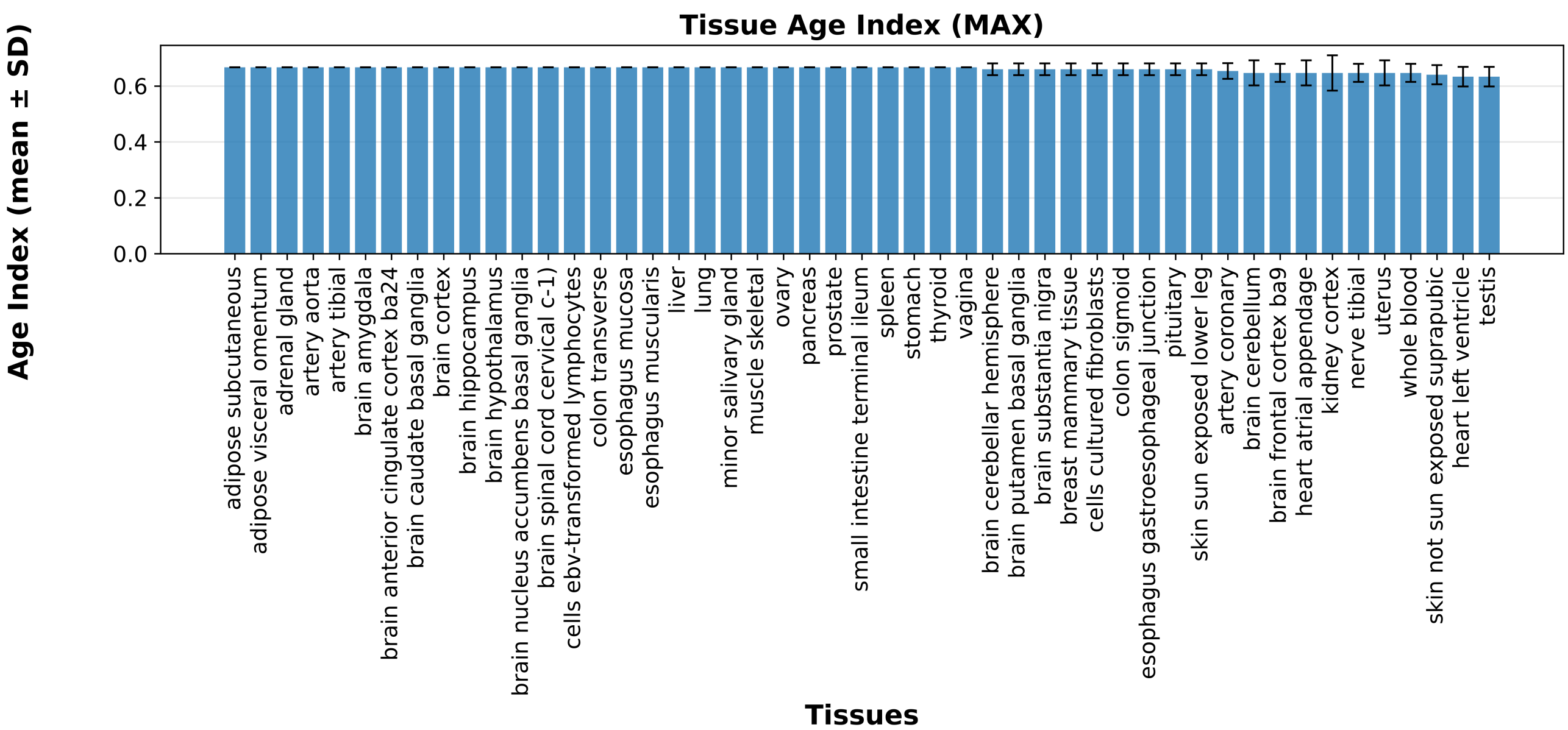

Fig. SM. 41. Tissue-wise Age Index (MAX) under INSERT perturbation ($p = 10\%$).

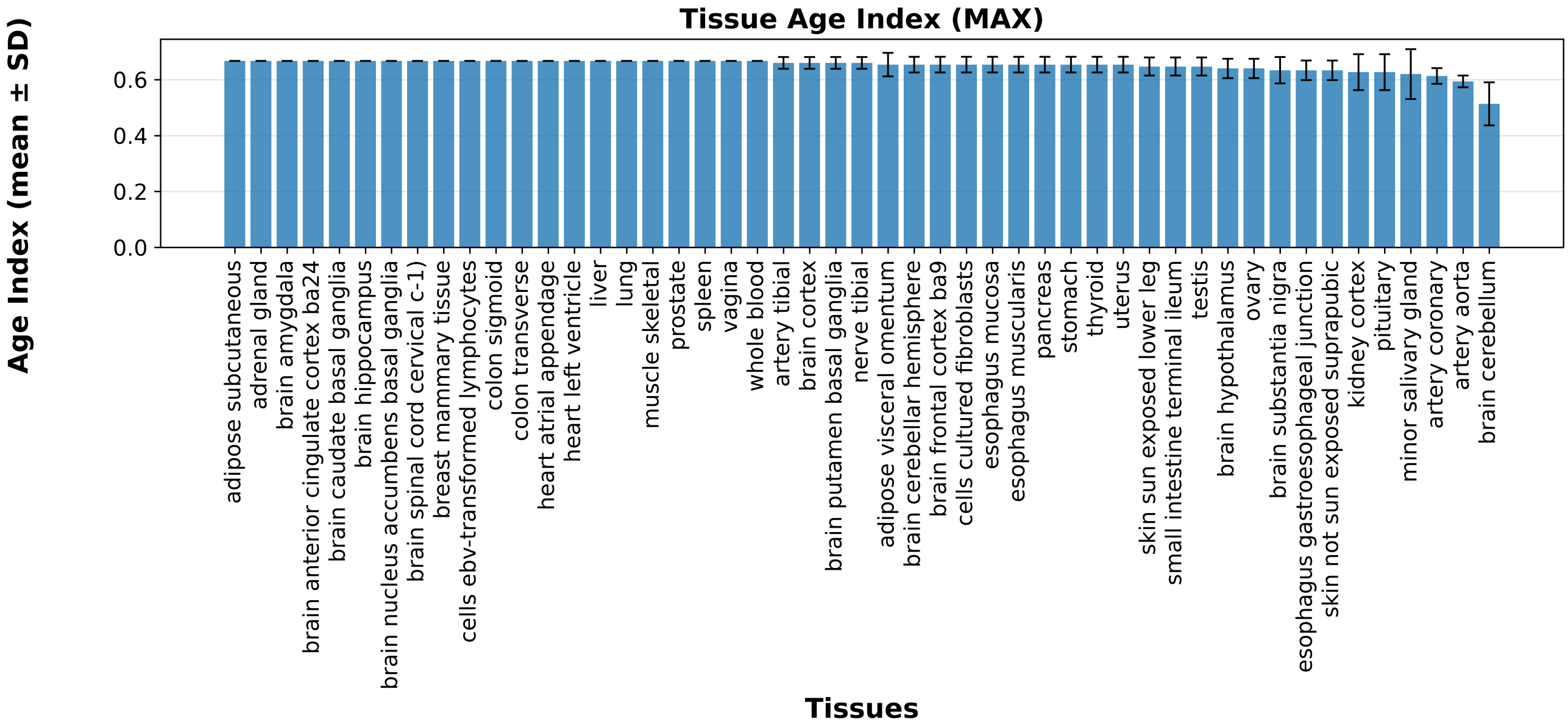

Fig. SM. 42. Tissue-wise Age Index (MAX) under DELETE perturbation ($p = 1\%$).

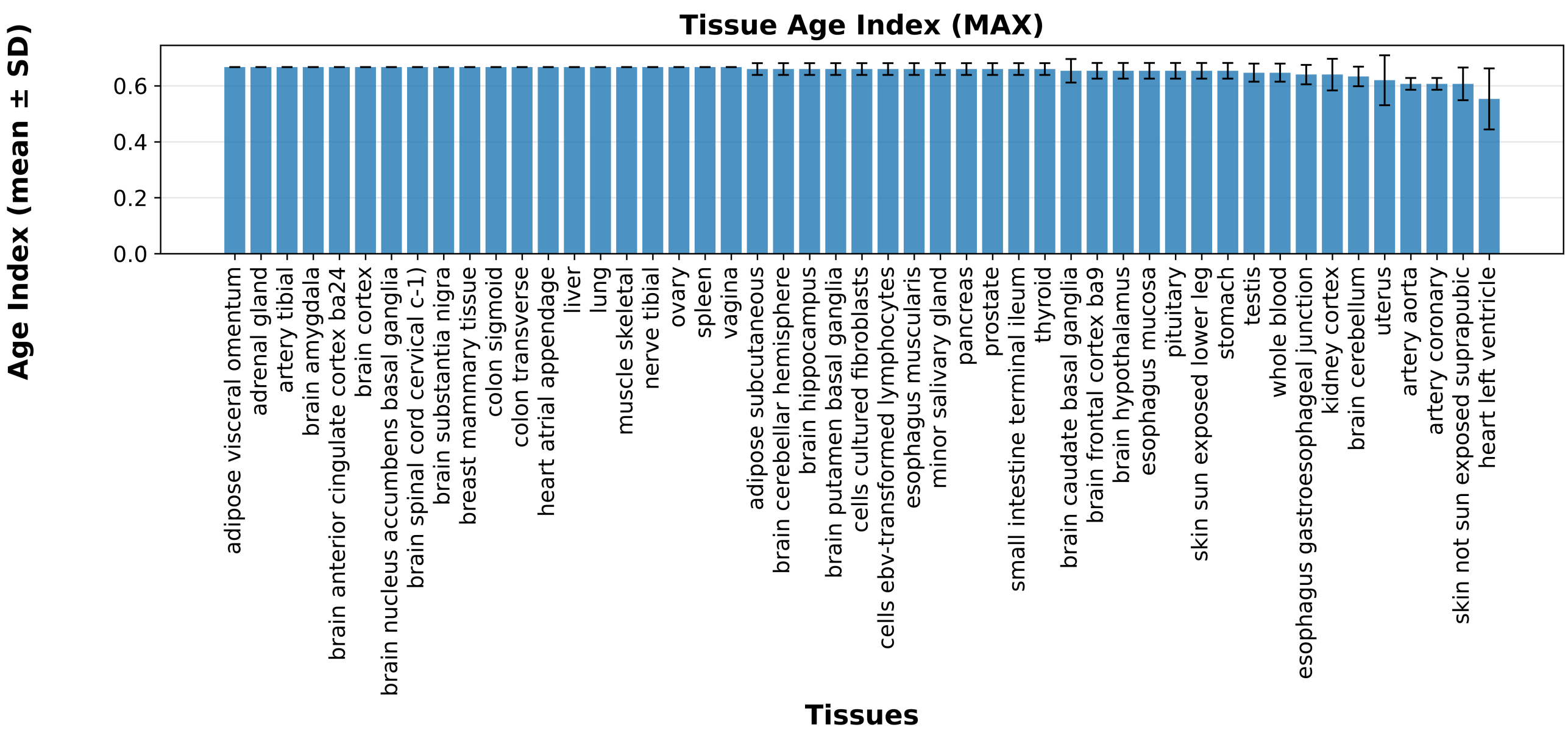

Fig. SM. 43. Tissue-wise Age Index (MAX) under DELETE perturbation ($p = 2\%$).

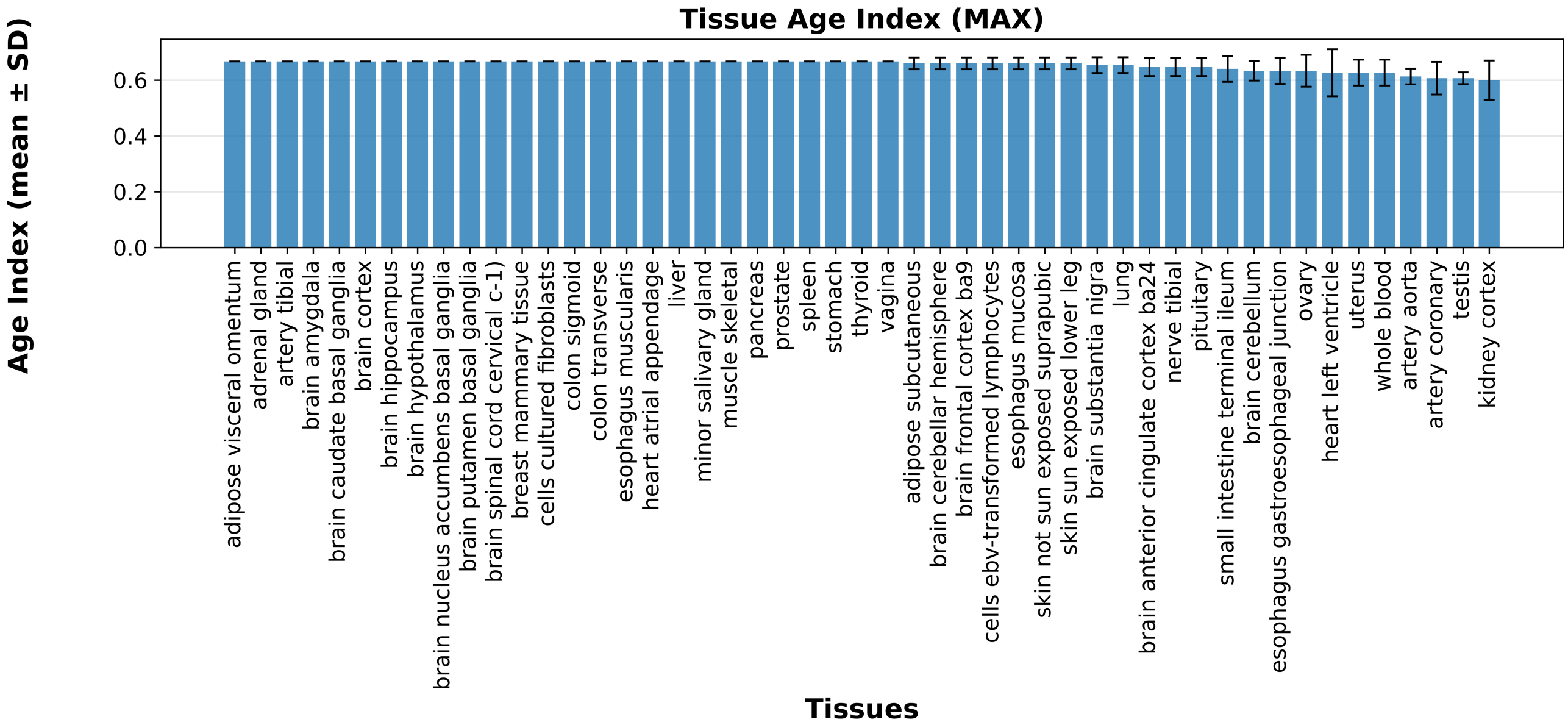

Fig. SM. 44. Tissue-wise Age Index (MAX) under DELETE perturbation ($p = 4\%$).

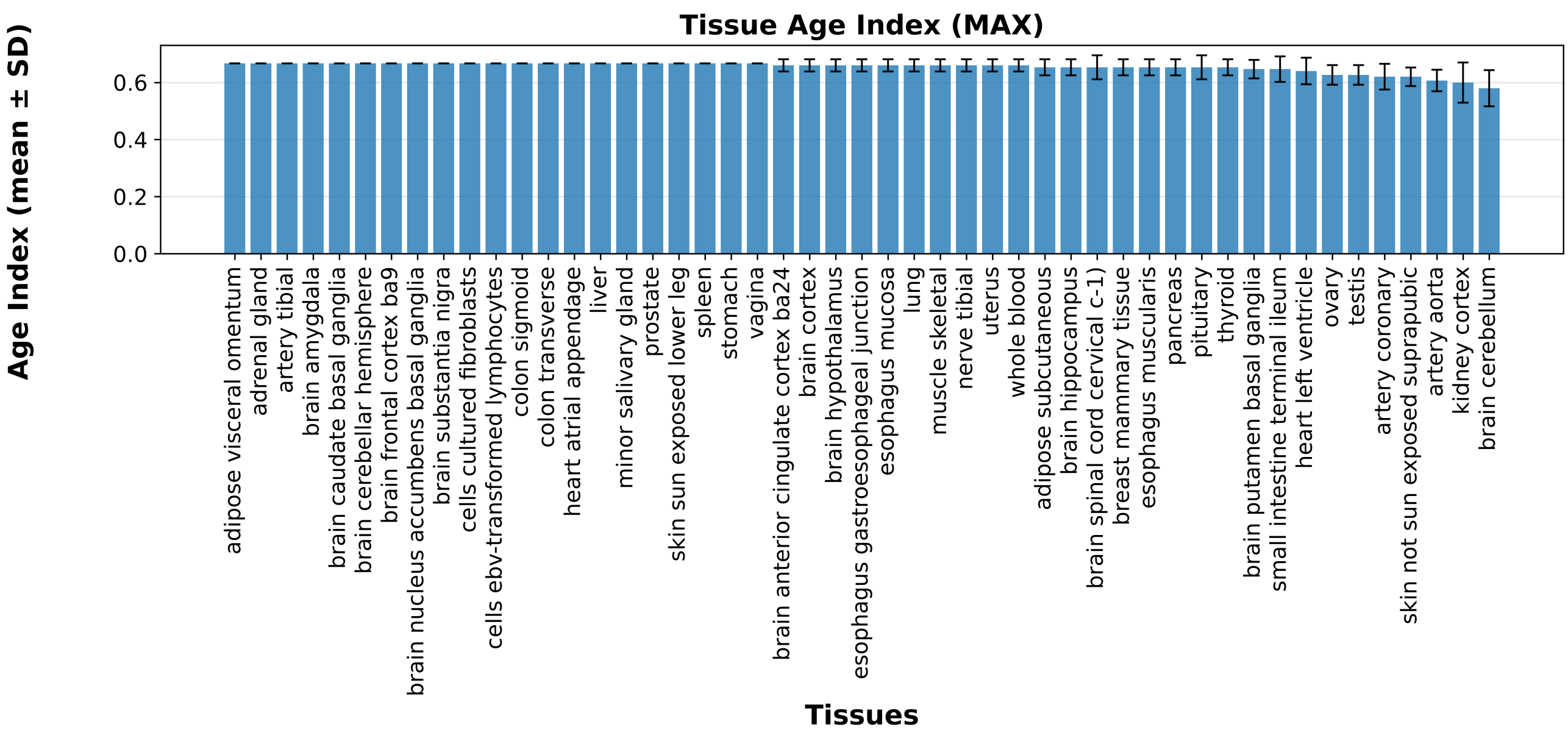

Fig. SM. 45. Tissue-wise Age Index (MAX) under DELETE perturbation ($p = 5\%$).

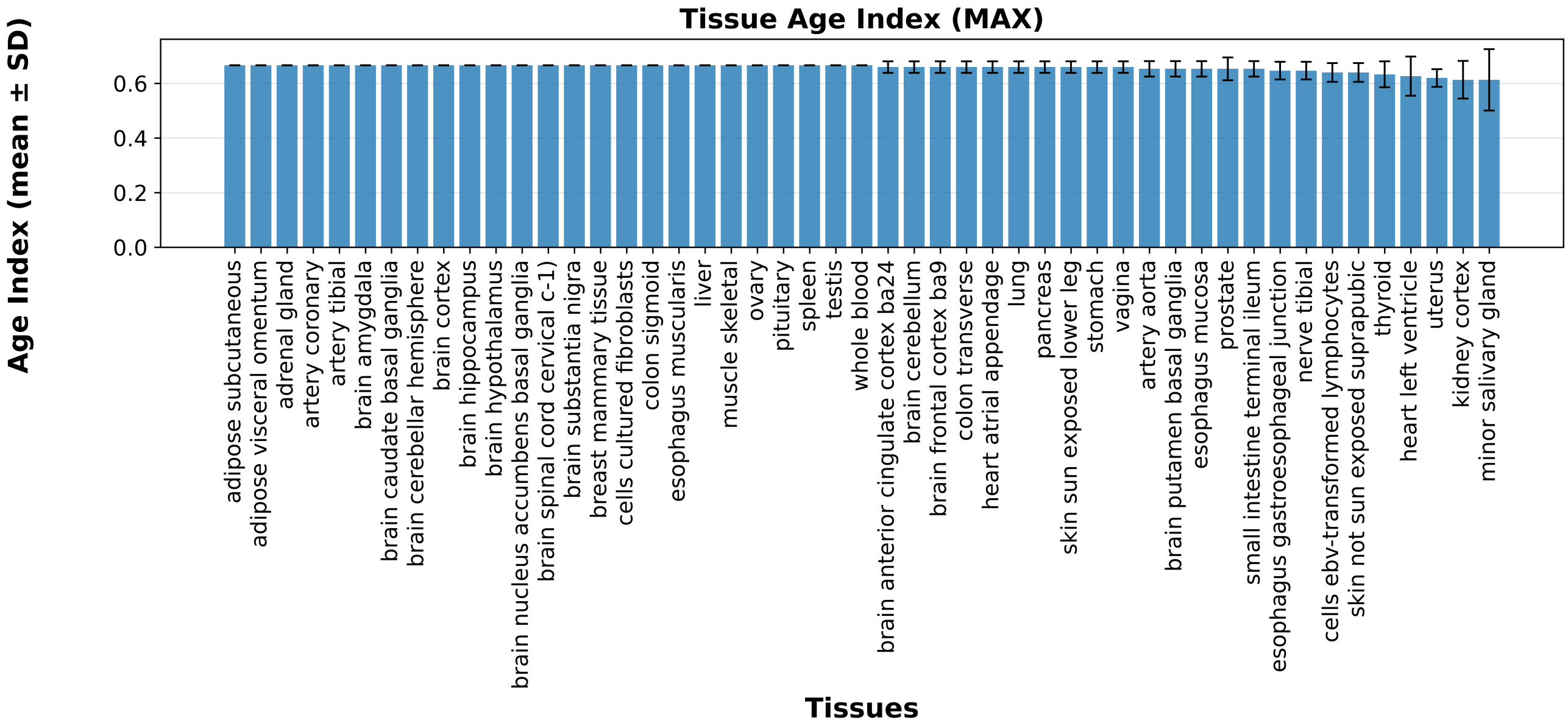

Fig. SM. 46. Tissue-wise Age Index (MAX) under DELETE perturbation ($p = 10\%$).

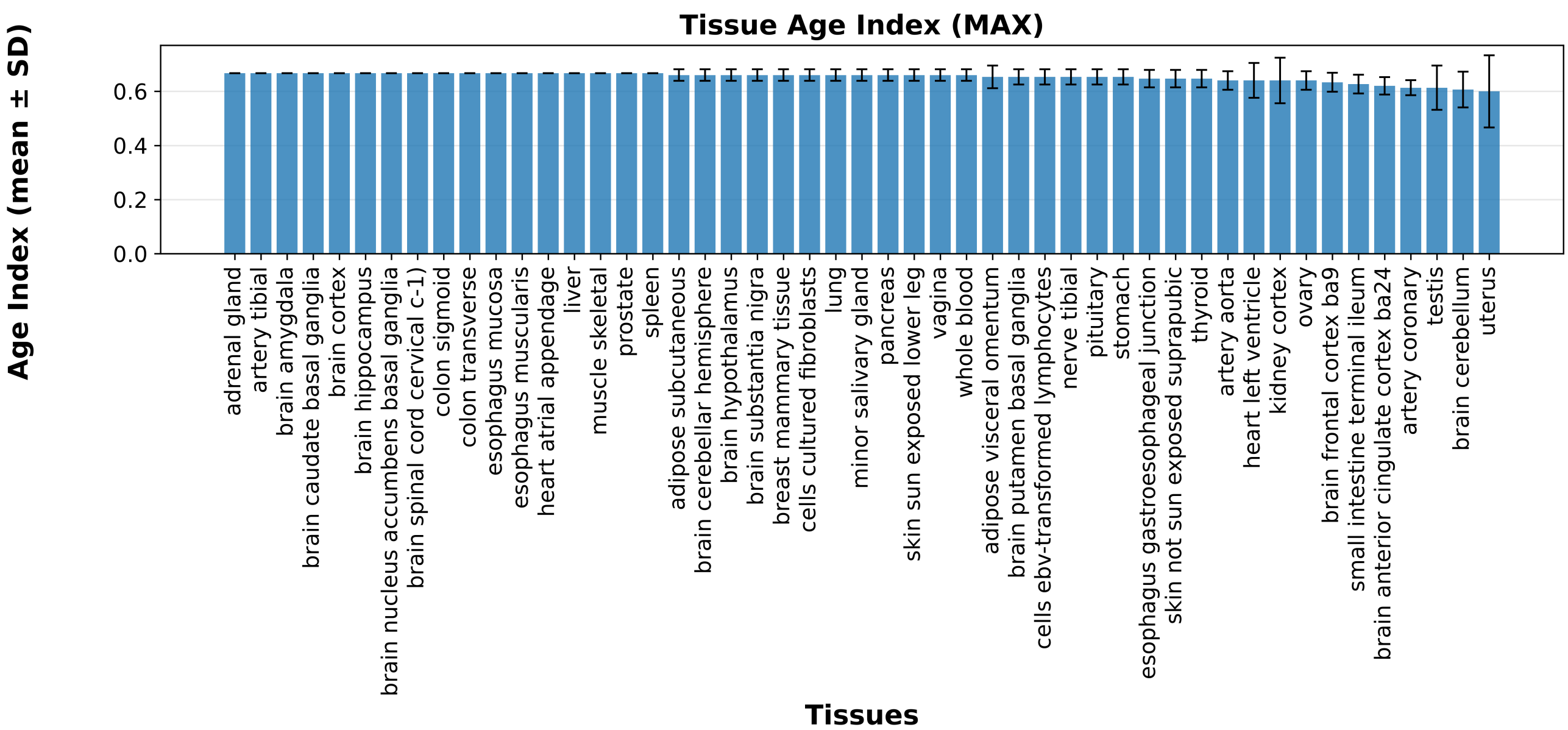

Fig. SM. 47. Tissue-wise Age Index (MAX) under REWIRE perturbation ($p = 1\%$).

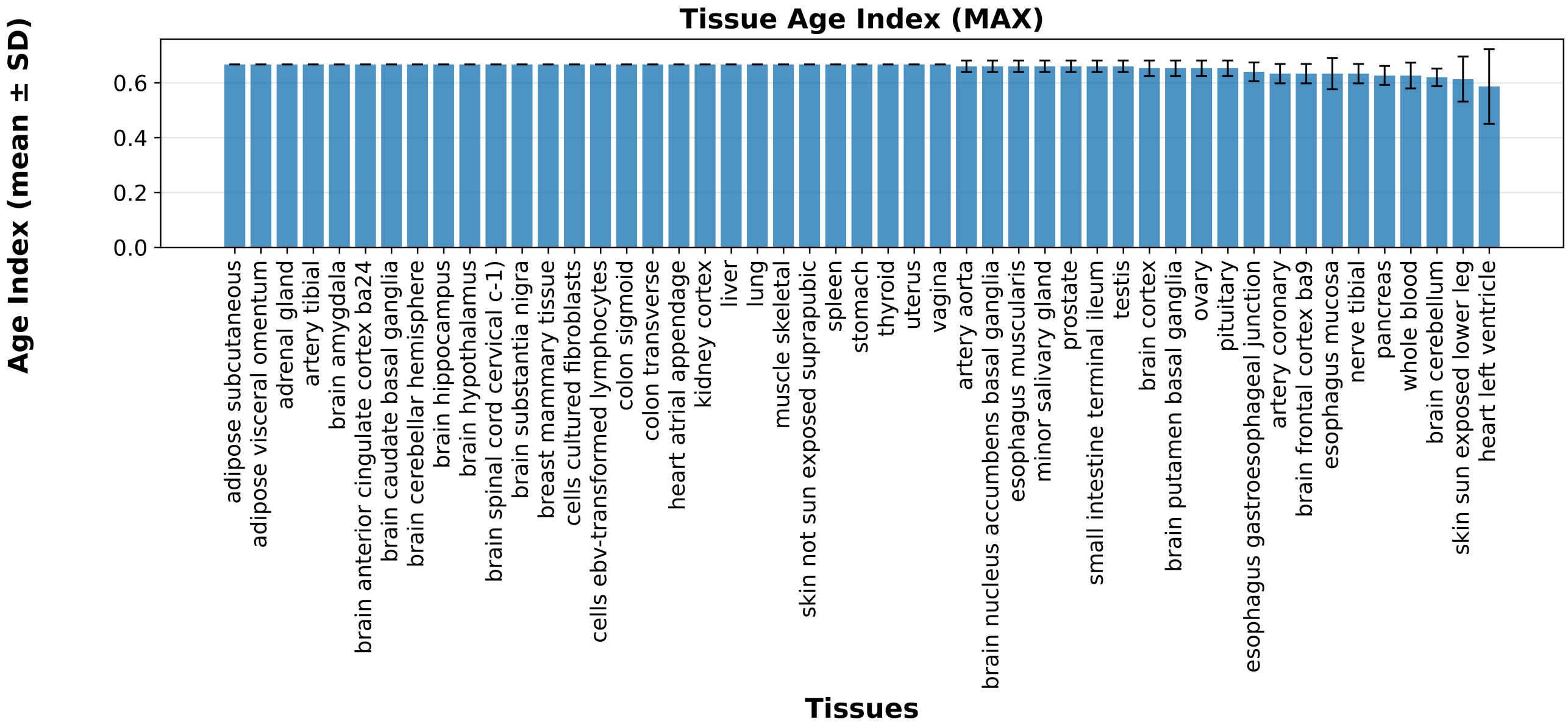

Fig. SM. 48. Tissue-wise Age Index (MAX) under REWIRE perturbation ($p = 2\%$).

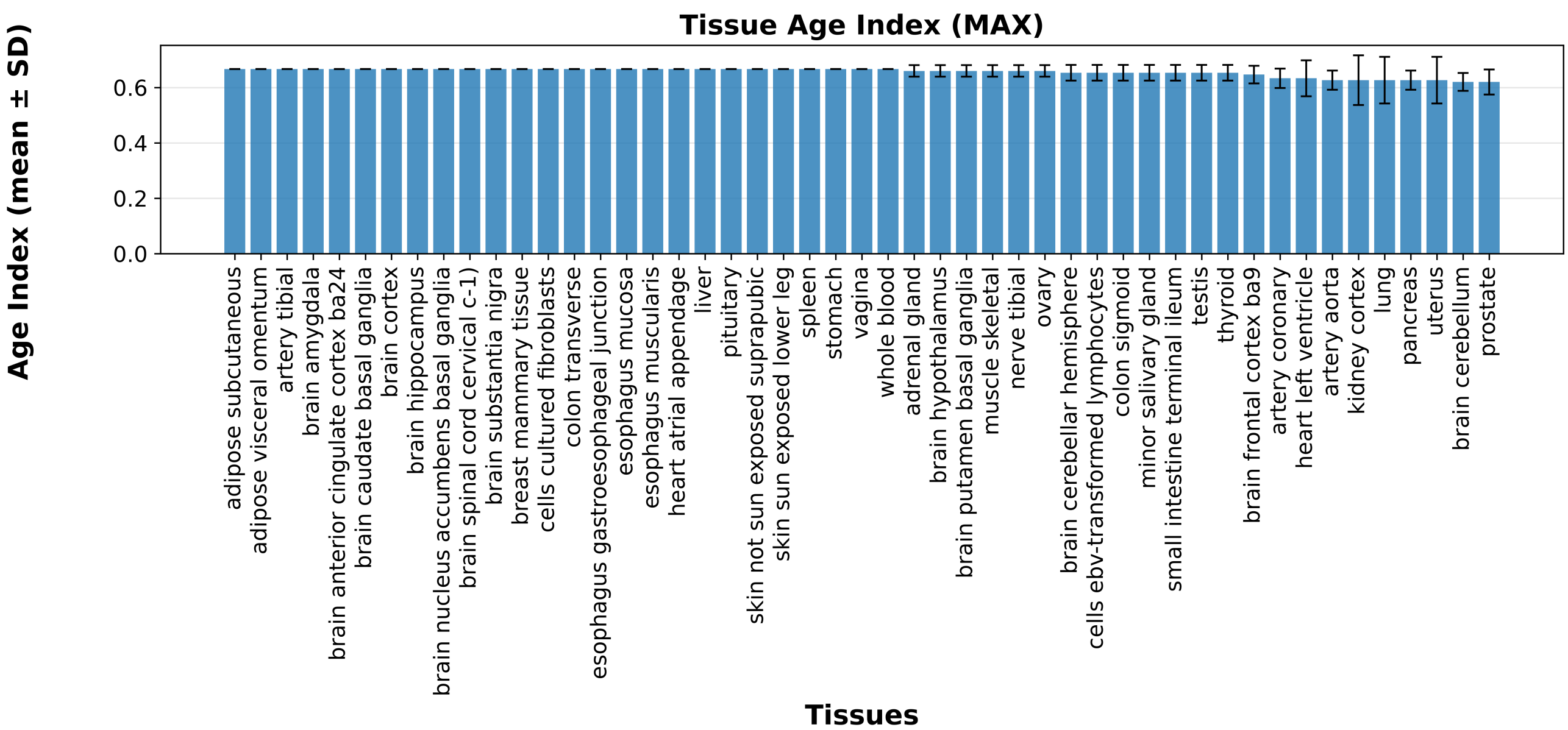

Fig. SM. 49. Tissue-wise Age Index (MAX) under REWIRE perturbation ($p = 4\%$).

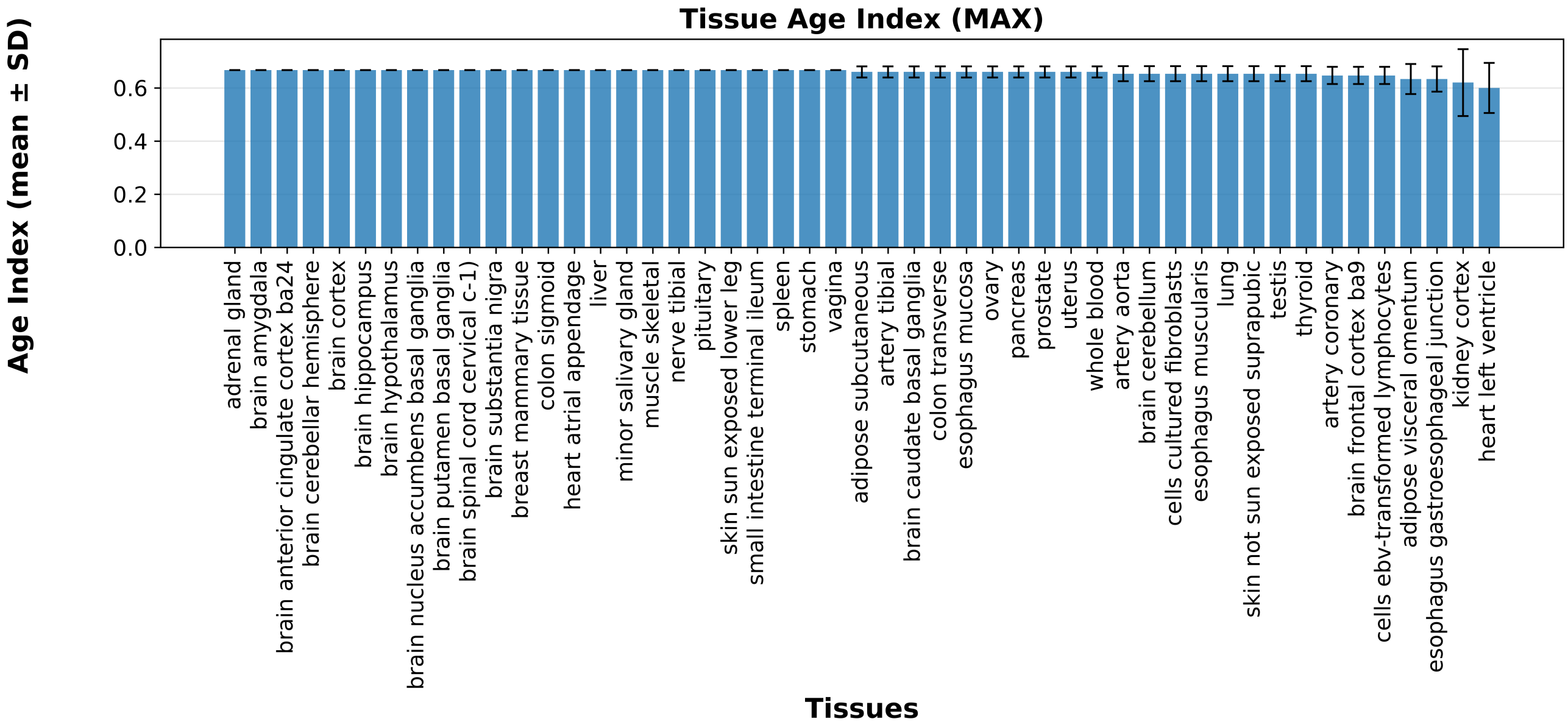

Fig. SM. 50. Tissue-wise Age Index (MAX) under REWIRE perturbation ($p = 5\%$).

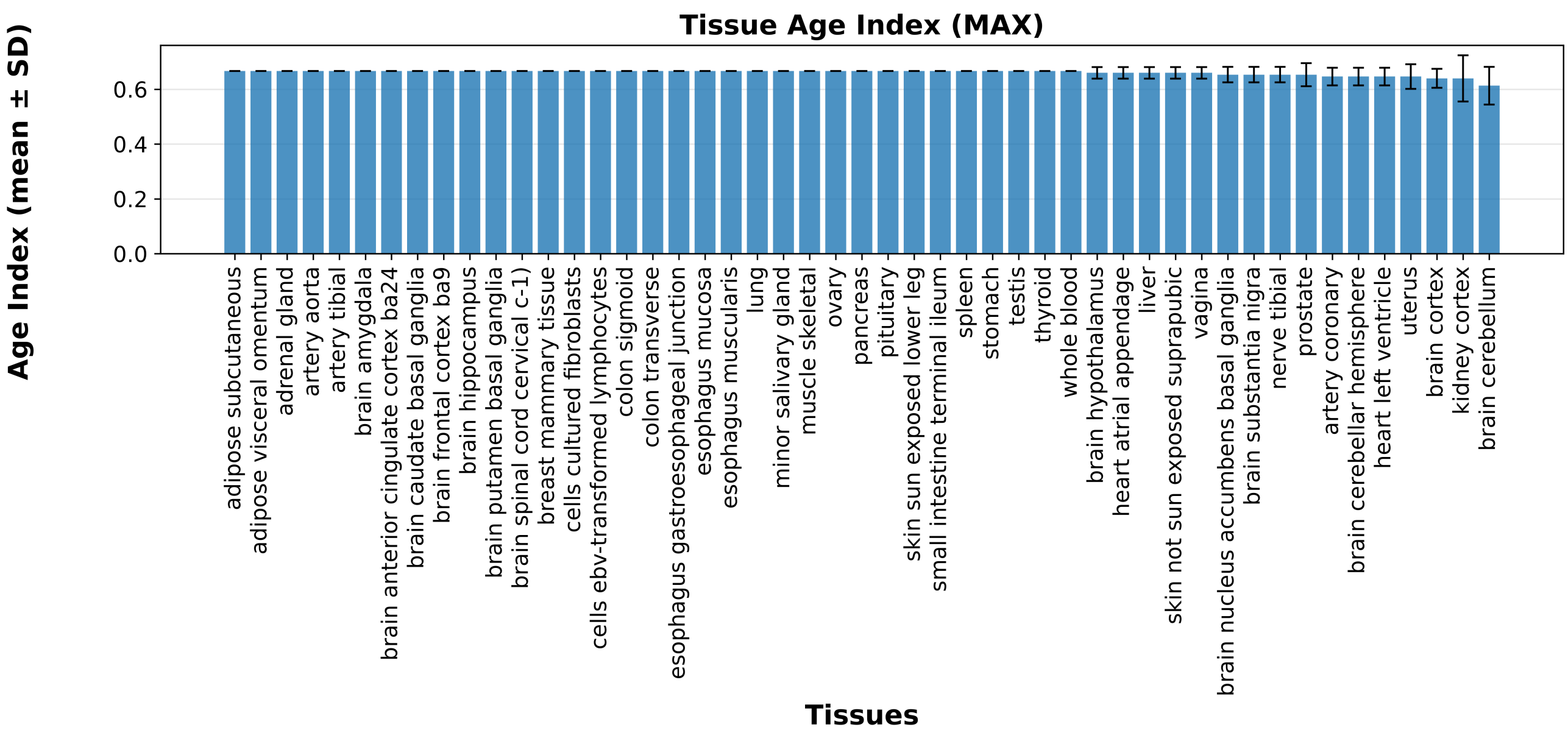

Fig. SM. 51. Tissue-wise Age Index (MAX) under REWIRE perturbation ($p = 10\%$).

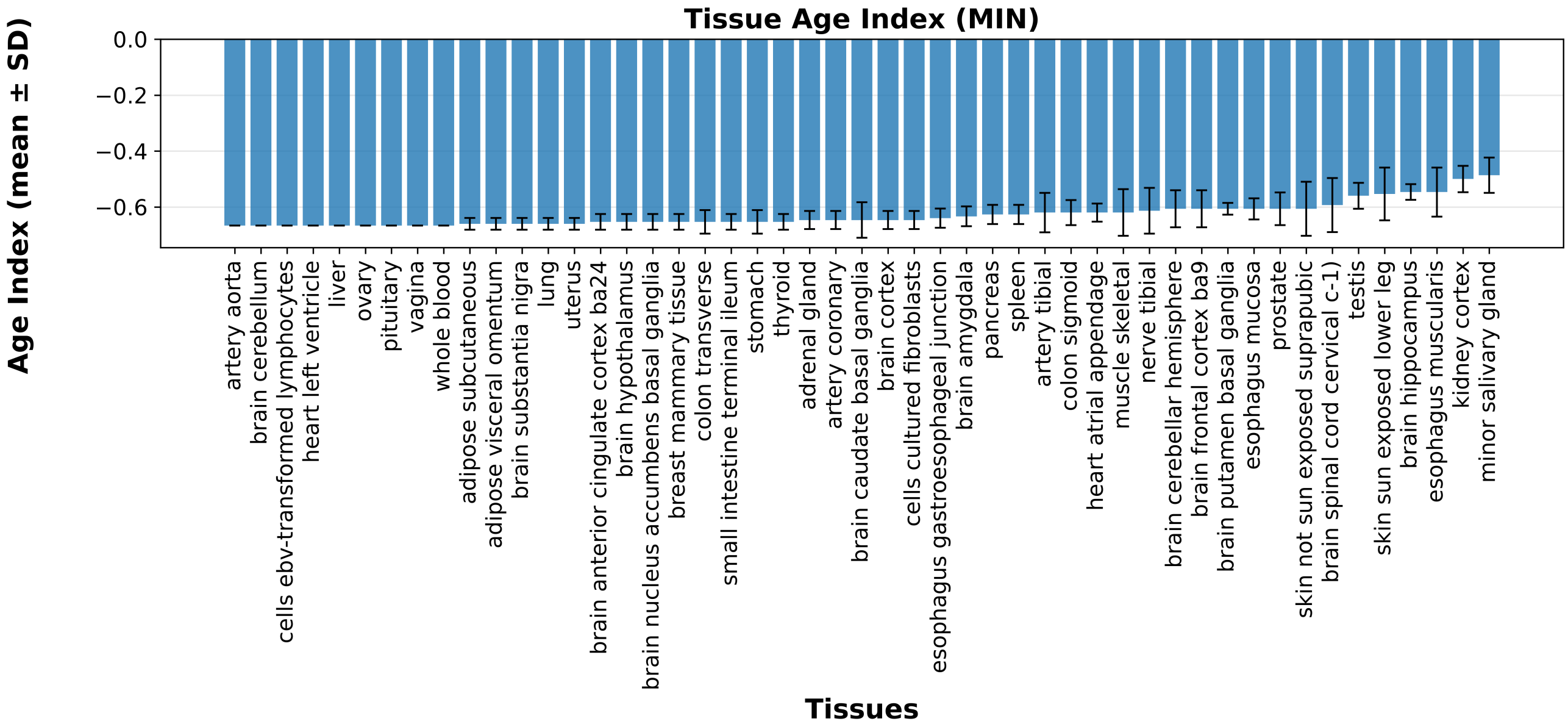

Fig. SM. 52. Tissue-wise Age Index (MIN) under INSERT perturbation ($p = 1\%$).

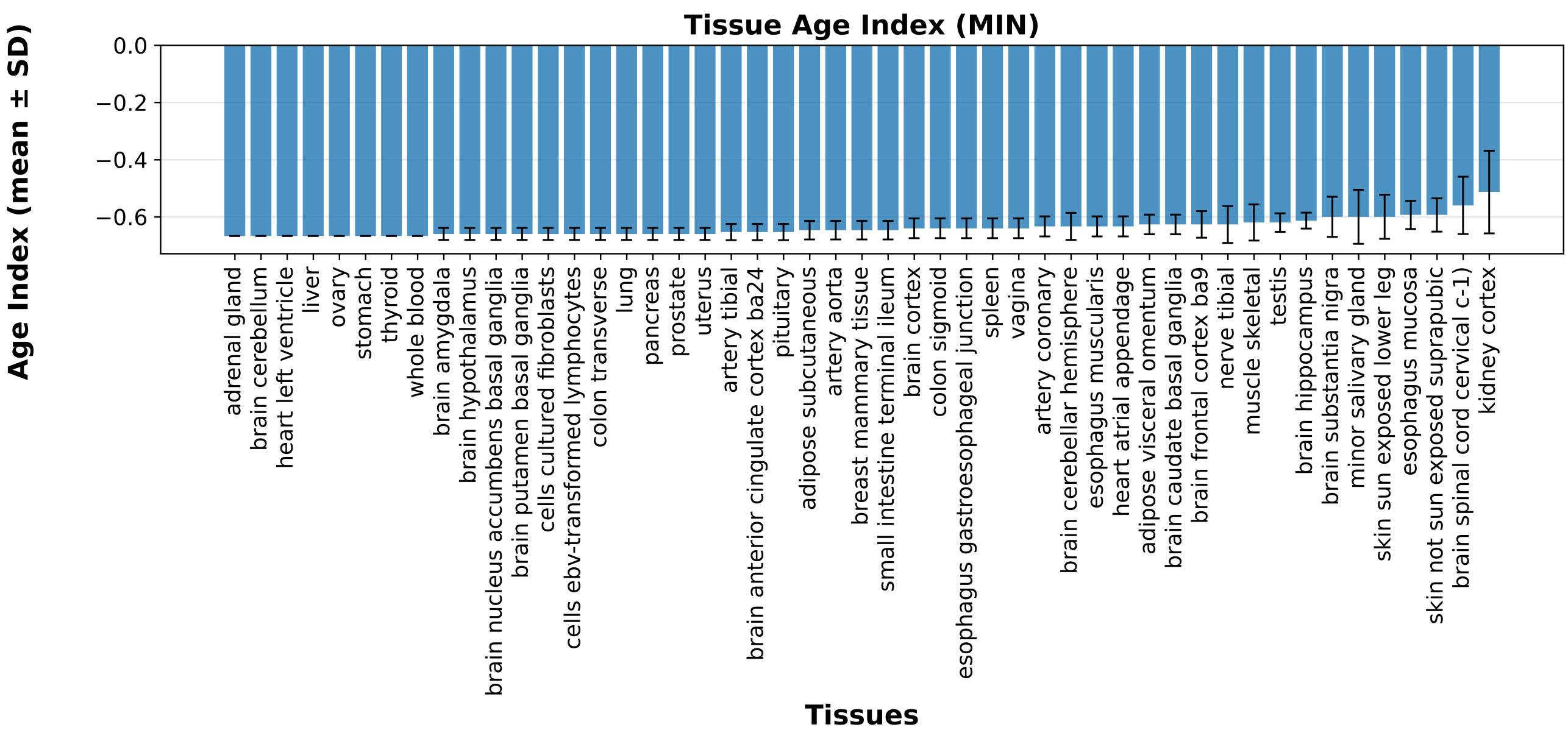

Fig. SM. 53. Tissue-wise Age Index (MIN) under INSERT perturbation ($p = 2\%$).

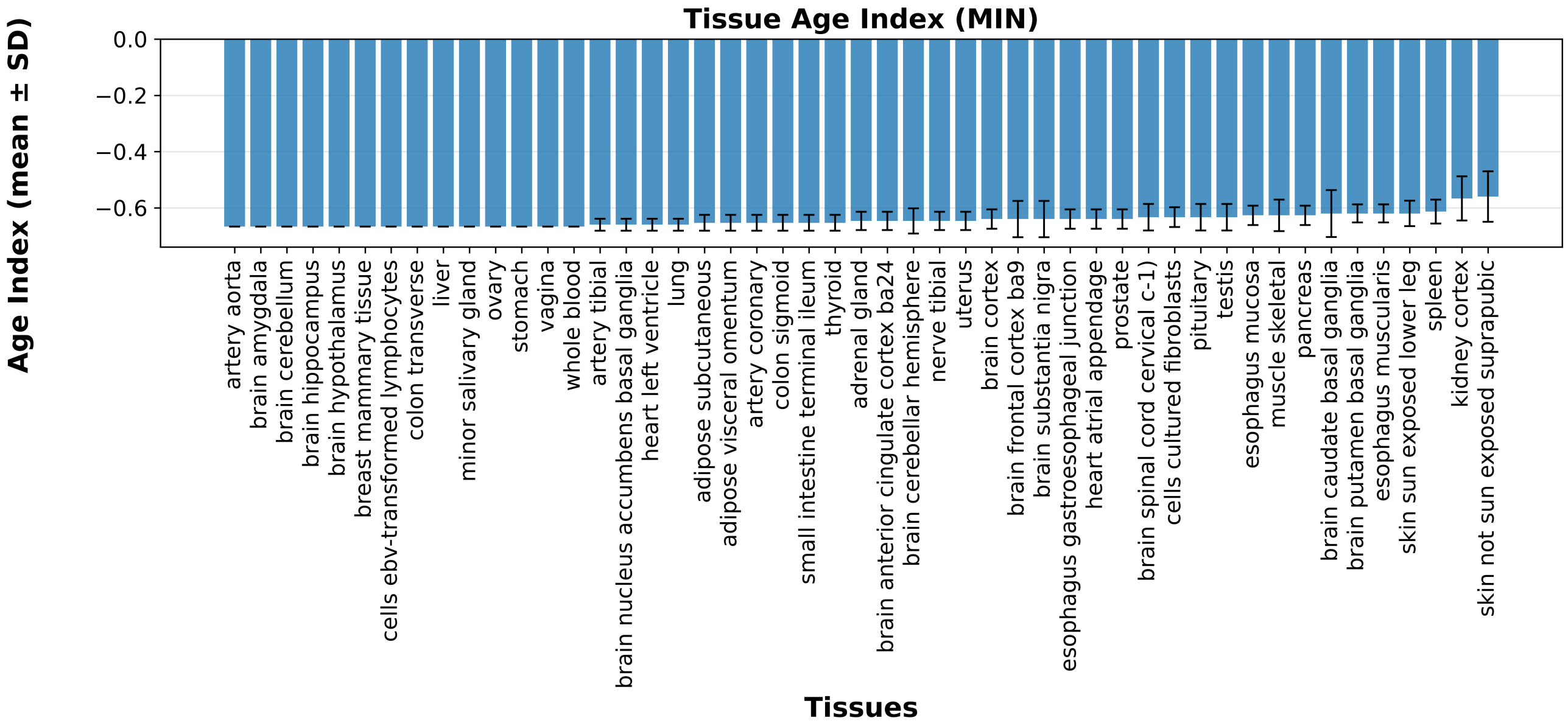

Fig. SM. 54. Tissue-wise Age Index (MIN) under INSERT perturbation ($p = 4\%$).

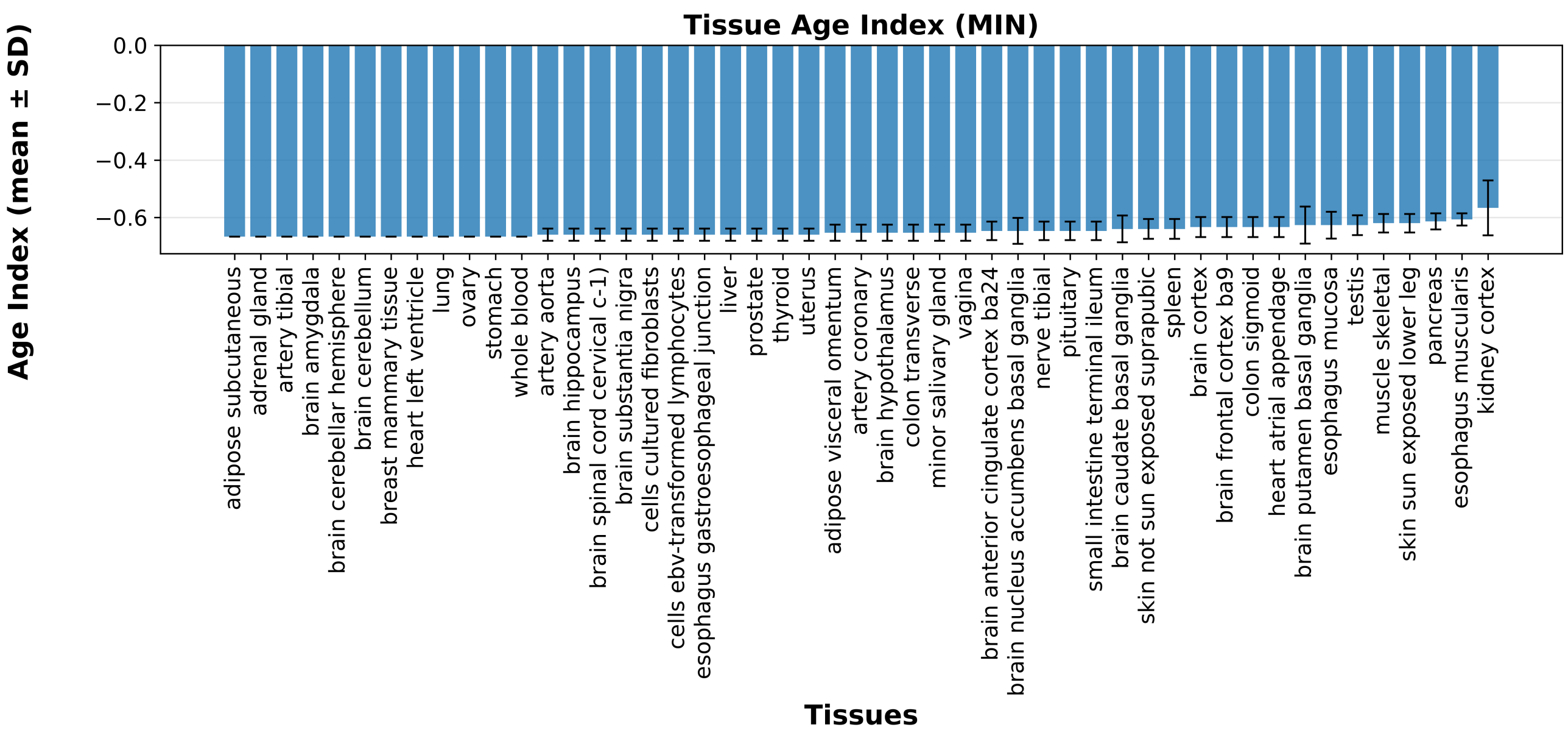

Fig. SM. 55. Tissue-wise Age Index (MIN) under INSERT perturbation ($p = 5\%$).

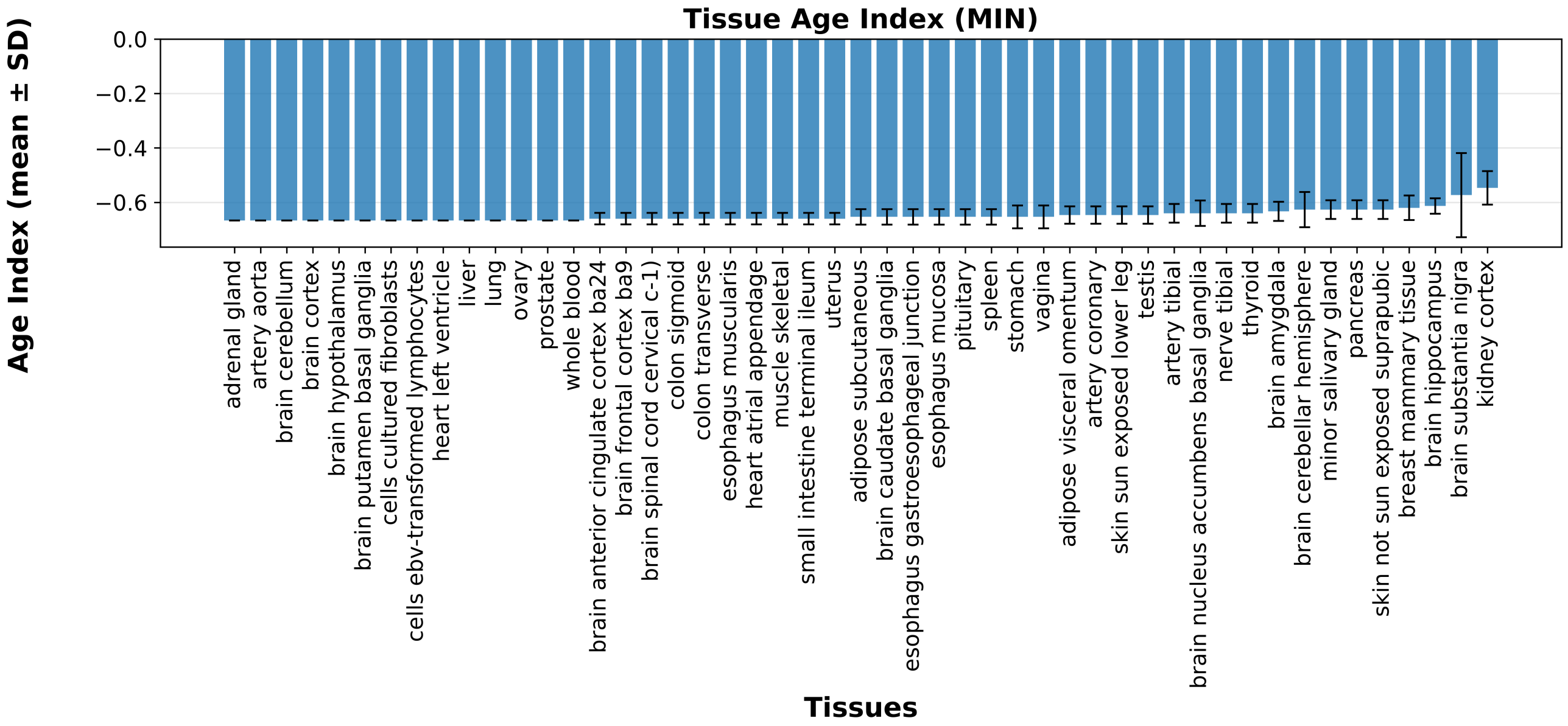

Fig. SM. 56. Tissue-wise Age Index (MIN) under INSERT perturbation ($p = 10\%$).

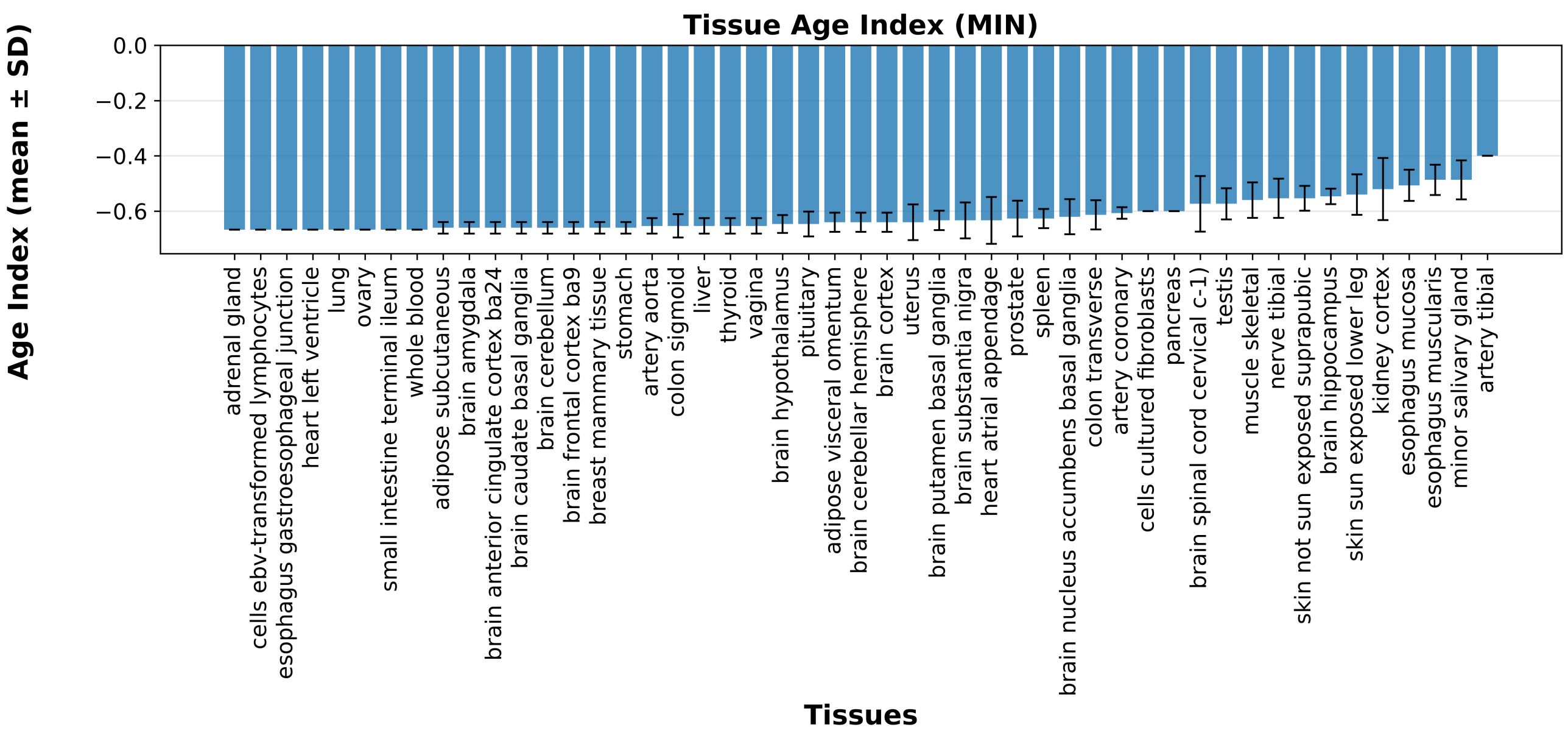

Fig. SM. 57. Tissue-wise Age Index (MIN) under DELETE perturbation ($p = 1\%$).

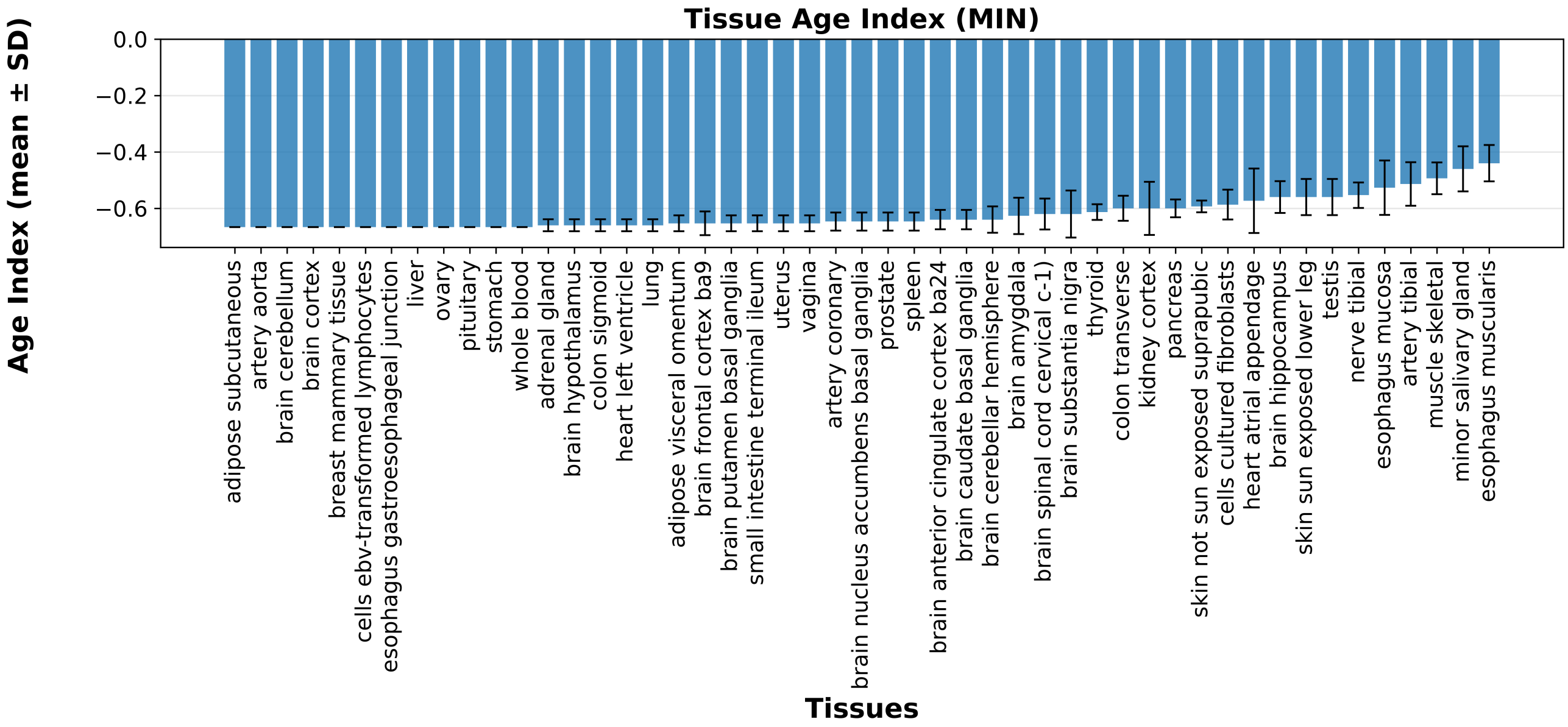

Fig. SM. 58. Tissue-wise Age Index (MIN) under DELETE perturbation ($p = 2\%$).

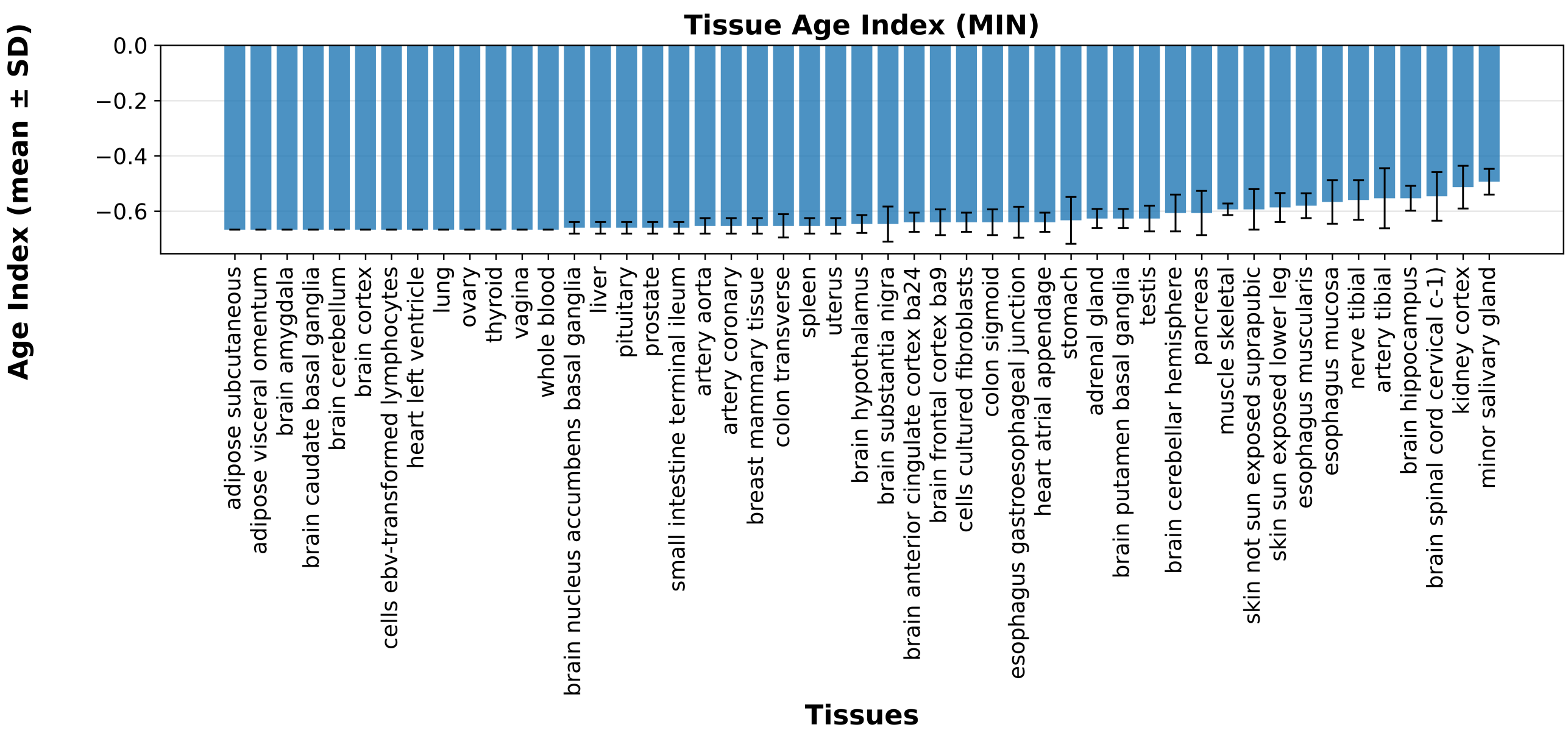

Fig. SM. 59. Tissue-wise Age Index (MIN) under DELETE perturbation ($p = 4\%$).

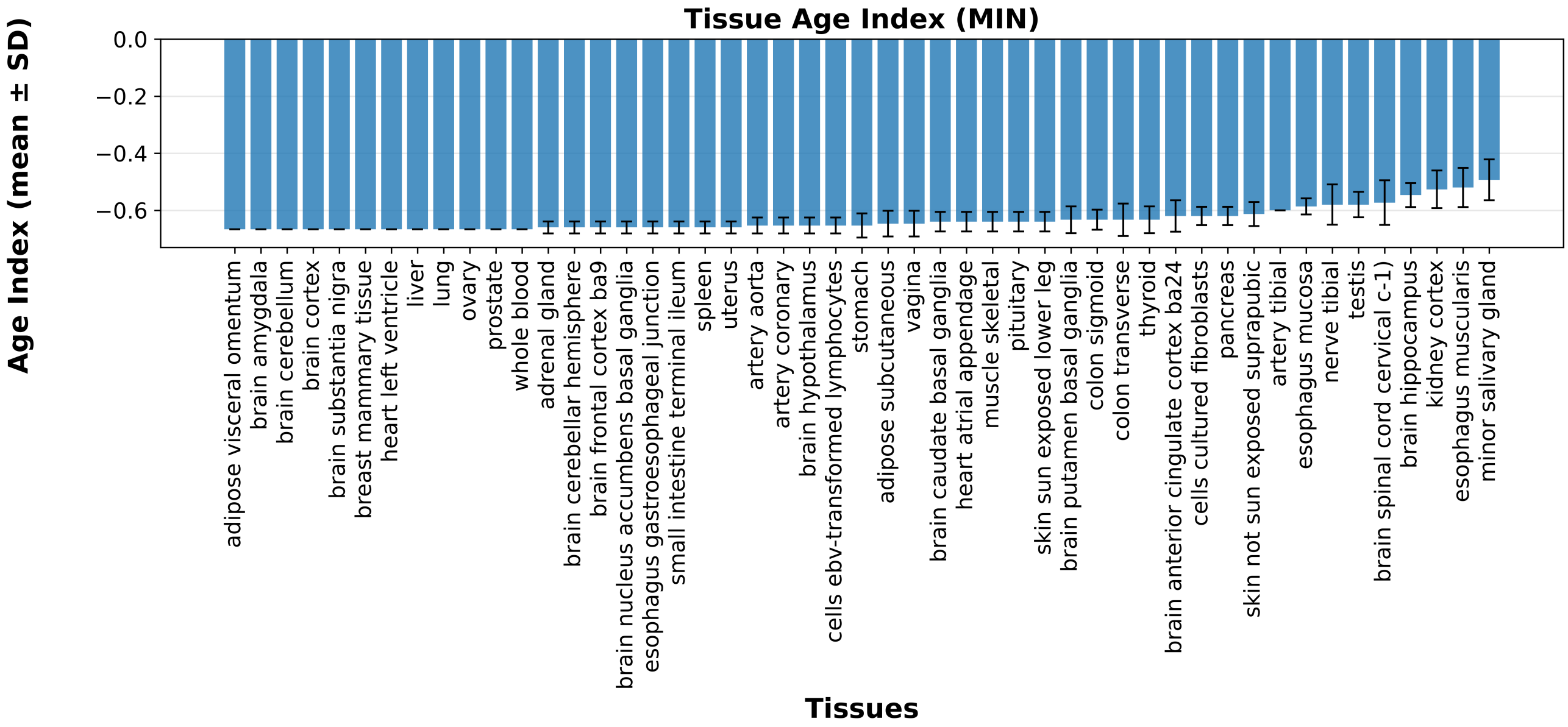

Fig. SM. 60. Tissue-wise Age Index (MIN) under DELETE perturbation ($p = 5\%$).

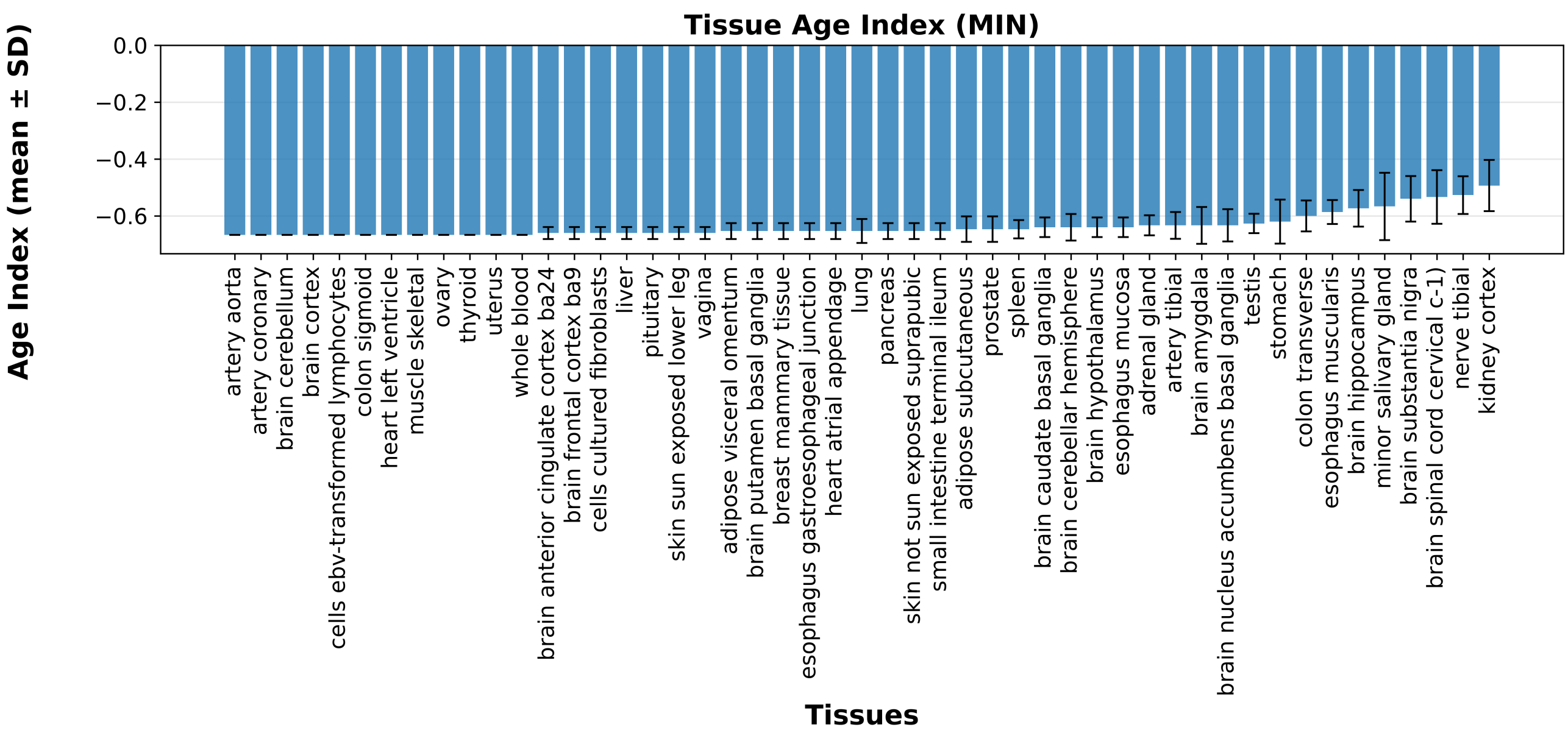

Fig. SM. 61. Tissue-wise Age Index (MIN) under DELETE perturbation ($p = 10\%$).

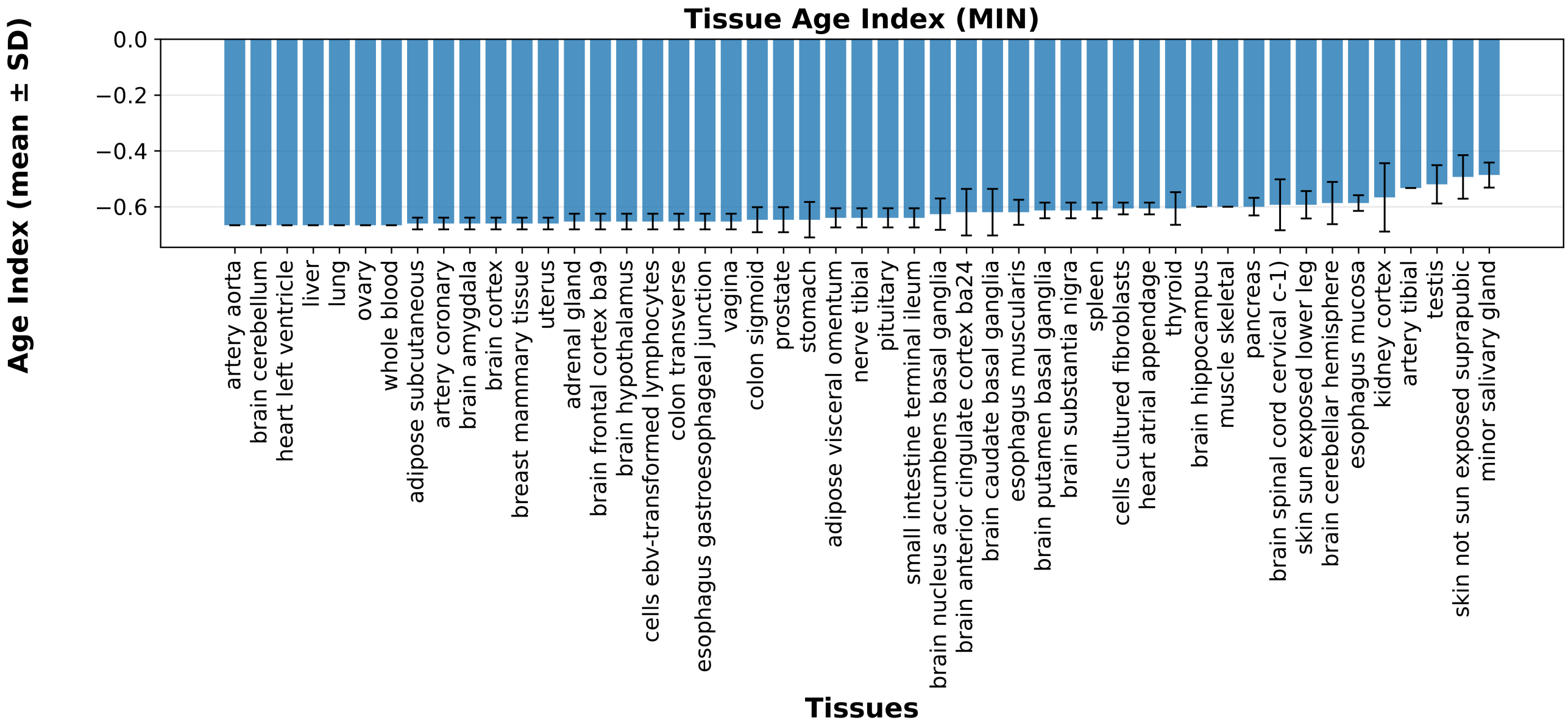

Fig. SM. 62. Tissue-wise Age Index (MIN) under REWIRE perturbation ($p = 1\%$).

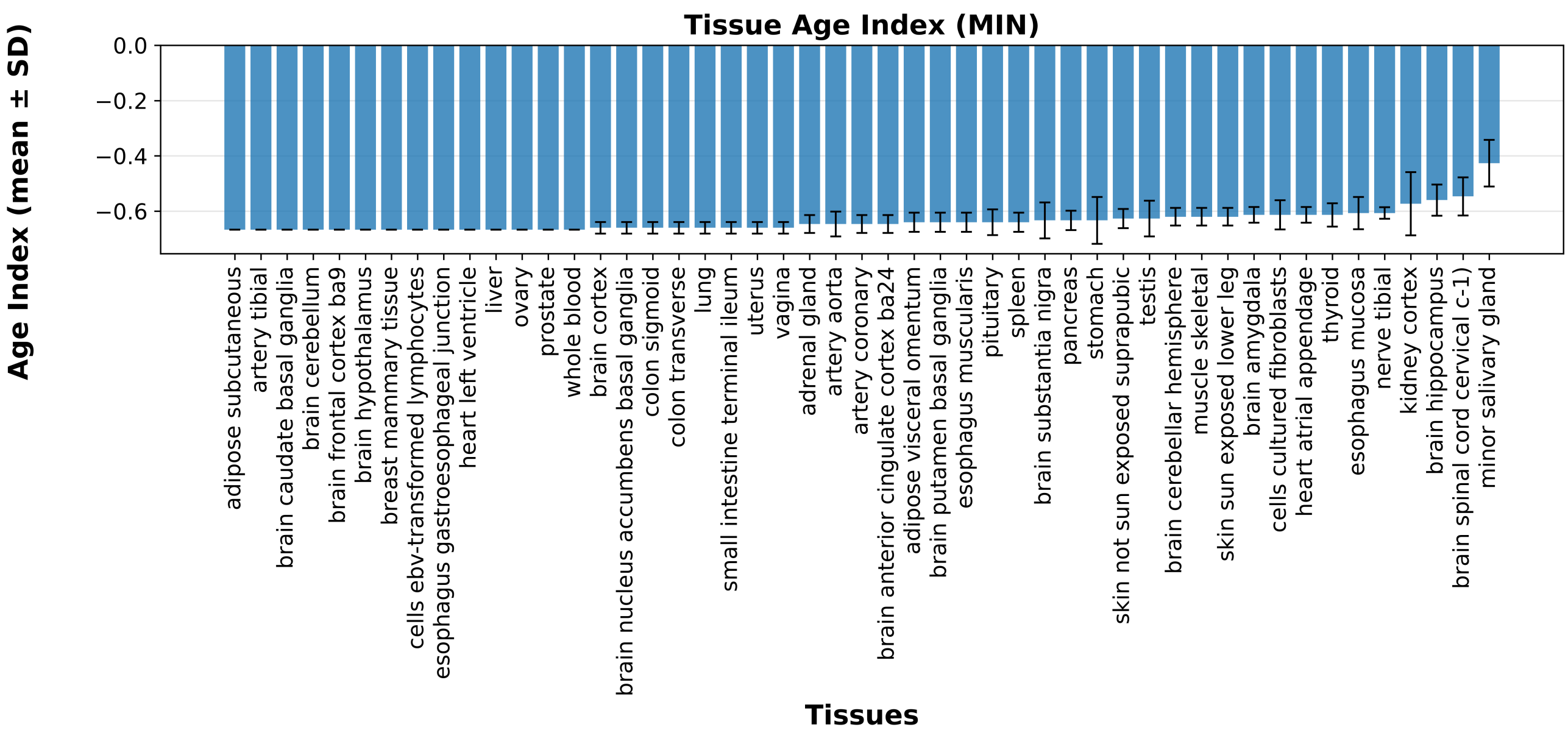

Fig. SM. 63. Tissue-wise Age Index (MIN) under REWIRE perturbation ($p = 2\%$).

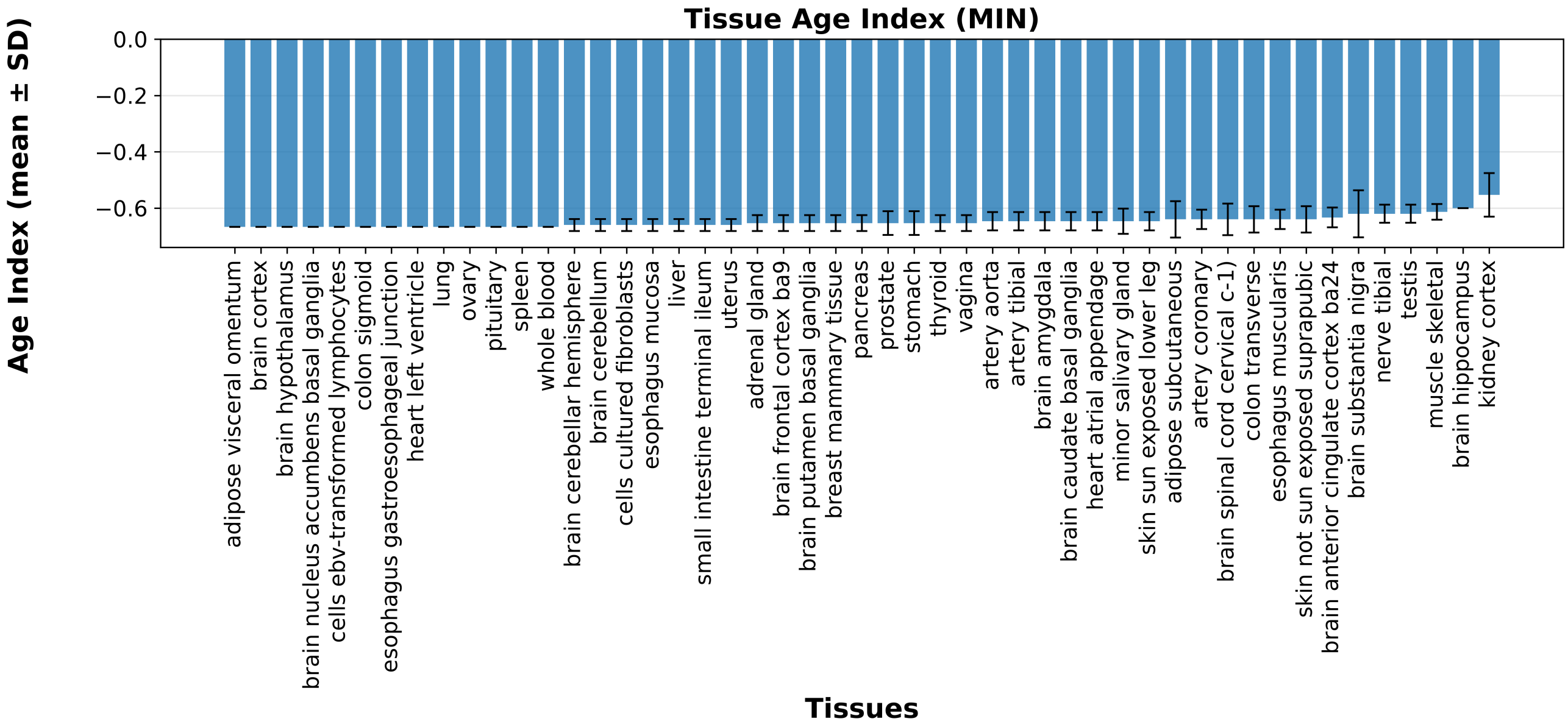

Fig. SM. 64. Tissue-wise Age Index (MIN) under REWIRE perturbation ($p = 4\%$).

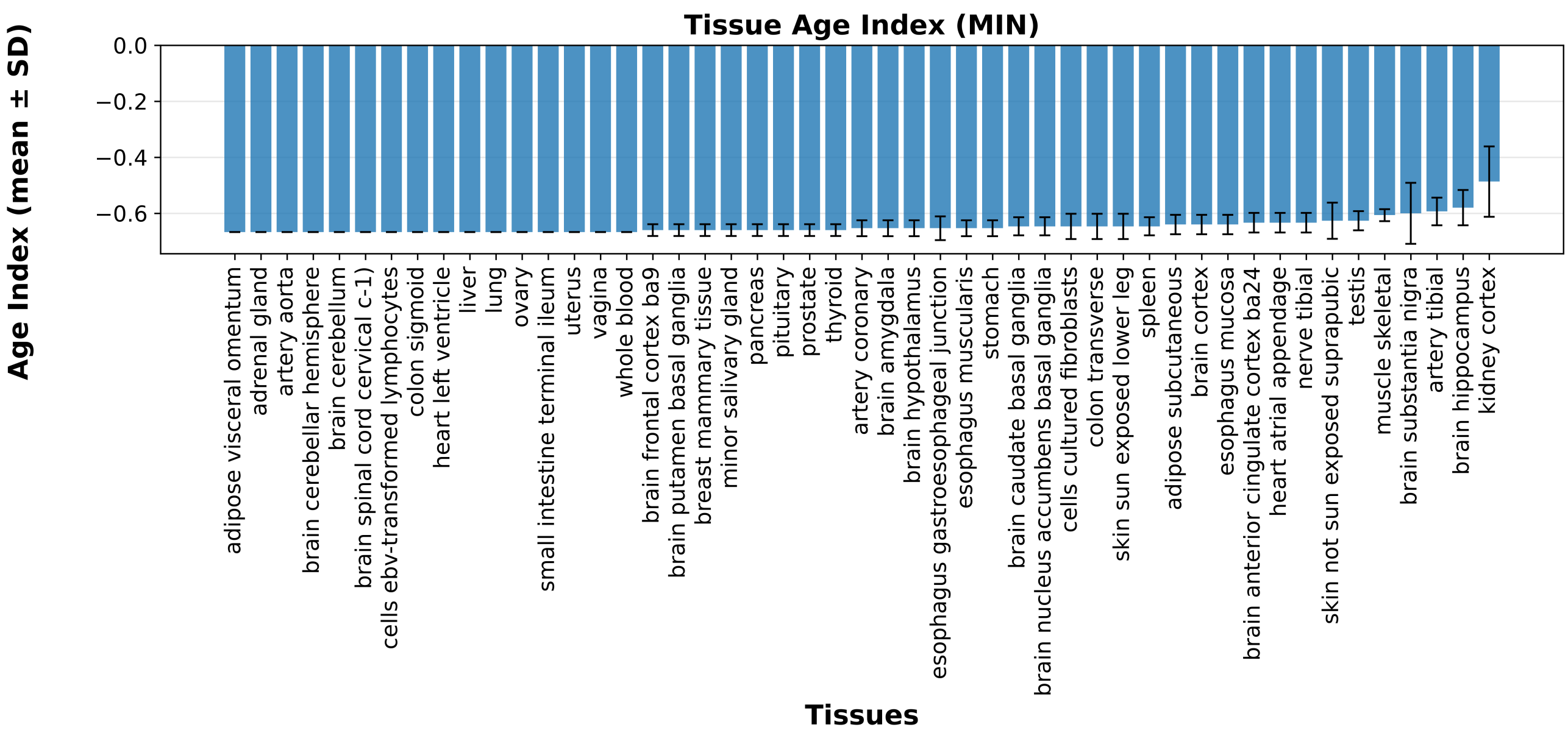

Fig. SM. 65. Tissue-wise Age Index (MIN) under REWIRE perturbation ($p = 5\%$).

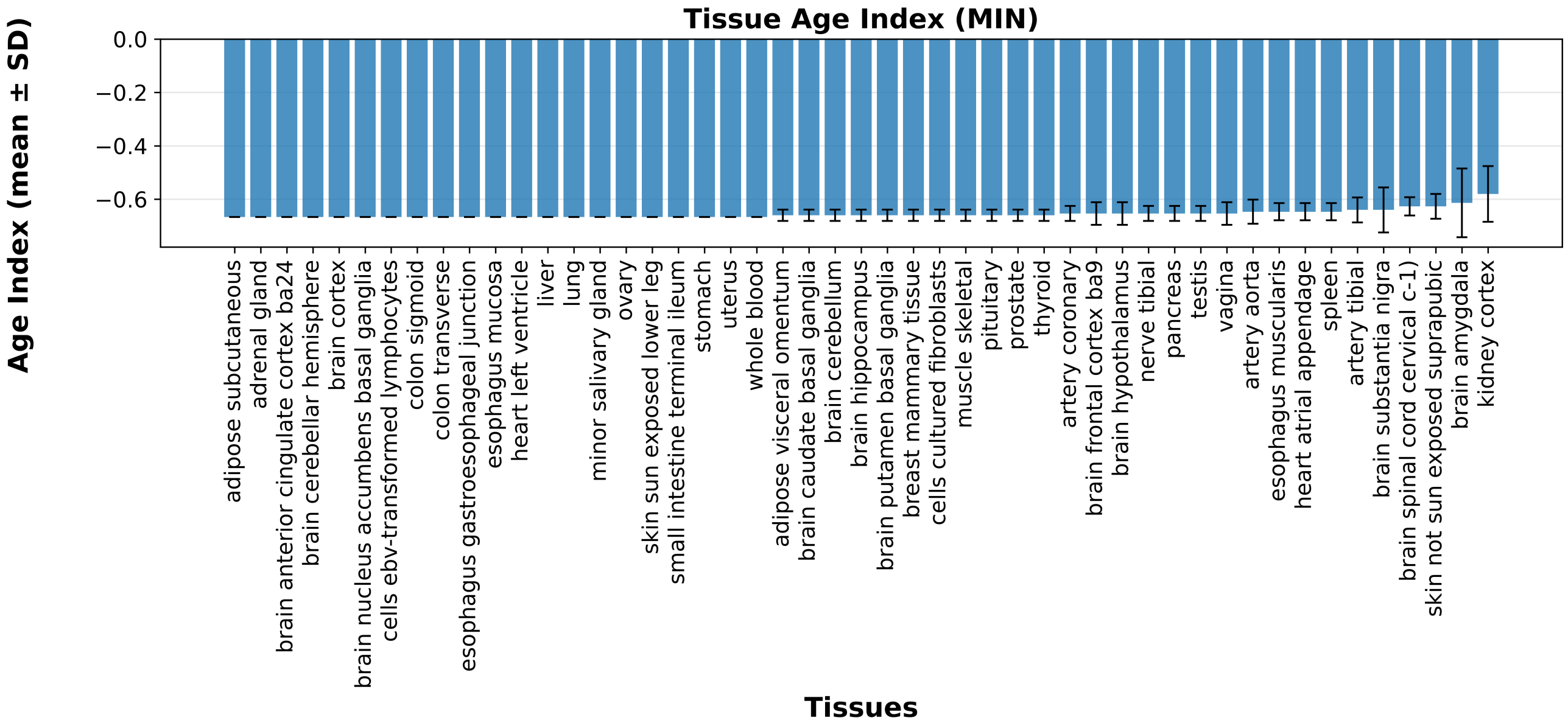

Fig. SM. 66. Tissue-wise Age Index (MIN) under REWIRE perturbation ($p = 10\%$).
